# Assessing the Risk of Bias in Randomized Clinical Trials With Large Language Models

**DOI:** 10.1001/jamanetworkopen.2024.12687

**Published:** 2024-05-22

**Authors:** Honghao Lai, Long Ge, Mingyao Sun, Bei Pan, Jiajie Huang, Liangying Hou, Qiuyu Yang, Jiayi Liu, Jianing Liu, Ziying Ye, Danni Xia, Weilong Zhao, Xiaoman Wang, Ming Liu, Jhalok Ronjan Talukdar, Jinhui Tian, Kehu Yang, Janne Estill

**Affiliations:** 1 Department of Health Policy and Management, School of Public Health, Lanzhou University, Lanzhou, China; 2 Evidence-Based Social Science Research Center, School of Public Health, Lanzhou University, Lanzhou, China; 3 Key Laboratory of Evidence Based Medicine and Knowledge Translation of Gansu Province, Lanzhou, China; 4 Evidence-Based Nursing Center, School of Nursing, Lanzhou University, Lanzhou, China; 5 Evidence-Based Medicine Center, School of Basic Medical Sciences, Lanzhou University, Lanzhou, China; 6 College of Nursing, Gansu University of Chinese Medicine, Lanzhou, China; 7 Department of Health Research Methods, Evidence, and Impact, McMaster University, Ontario, Canada; 8 Institute of Global Health, University of Geneva, Geneva, Switzerland

## Abstract

**Importance:**

Large language models (LLMs) may facilitate the labor-intensive process of systematic reviews. However, the exact methods and reliability remain uncertain.

**Objective:**

To explore the feasibility and reliability of using LLMs to assess risk of bias (ROB) in randomized clinical trials (RCTs).

**Design, Setting, and Participants:**

A survey study was conducted between August 10, 2023, and October 30, 2023. Thirty RCTs were selected from published systematic reviews.

**Main Outcomes and Measures:**

A structured prompt was developed to guide ChatGPT (LLM 1) and Claude (LLM 2) in assessing the ROB in these RCTs using a modified version of the Cochrane ROB tool developed by the CLARITY group at McMaster University. Each RCT was assessed twice by both models, and the results were documented. The results were compared with an assessment by 3 experts, which was considered a criterion standard. Correct assessment rates, sensitivity, specificity, and *F1* scores were calculated to reflect accuracy, both overall and for each domain of the Cochrane ROB tool; consistent assessment rates and Cohen κ were calculated to gauge consistency; and assessment time was calculated to measure efficiency. Performance between the 2 models was compared using risk differences.

**Results:**

Both models demonstrated high correct assessment rates. LLM 1 reached a mean correct assessment rate of 84.5% (95% CI, 81.5%-87.3%), and LLM 2 reached a significantly higher rate of 89.5% (95% CI, 87.0%-91.8%). The risk difference between the 2 models was 0.05 (95% CI, 0.01-0.09). In most domains, domain-specific correct rates were around 80% to 90%; however, sensitivity below 0.80 was observed in domains 1 (random sequence generation), 2 (allocation concealment), and 6 (other concerns). Domains 4 (missing outcome data), 5 (selective outcome reporting), and 6 had *F1* scores below 0.50. The consistent rates between the 2 assessments were 84.0% for LLM 1 and 87.3% for LLM 2. LLM 1’s κ exceeded 0.80 in 7 and LLM 2’s in 8 domains. The mean (SD) time needed for assessment was 77 (16) seconds for LLM 1 and 53 (12) seconds for LLM 2.

**Conclusions:**

In this survey study of applying LLMs for ROB assessment, LLM 1 and LLM 2 demonstrated substantial accuracy and consistency in evaluating RCTs, suggesting their potential as supportive tools in systematic review processes.

## Key Points

**Question:**

Are large language models reliable for assessing risk of bias (ROB) in randomized clinical trials (RCTs)?

**Findings:**

In this survey study with 2 large language models and 3 experts assessing 30 RCTs, a structured prompt was developed to guide the assessment of ROB, resulting in high accuracy rates for both large language models (>84.5%), compared with human reviewers, across 10 specific domains.

**Meaning:**

These findings suggest that 2 large language models have substantial accuracy in assessing ROB in RCTs, suggesting their potential as supportive tools in systematic review processes.

## Teaser

This survey study evaluates the feasibility and reliability of using large language models to assess risk of bias in randomized clinical trials.

## Introduction

Systematic reviews synthesize and evaluate existing research, guiding clinical decisions and informing health guidelines.^1,2^ The fast pace of medical progress and evidence turnover has heightened demand for timely evidence synthesis.^2^ While innovative approaches such as living systematic reviews have been proposed to enhance the efficiency of systematic review production,^3,4^ a substantial portion of clinical practice still lacks the support of up to date, high-quality evidence.^5^ The time and resources required to produce high-quality reviews contribute to this predicament, with the process of objectively assessing the methodological flaws in included studies, such as the risk of bias (ROB), being particularly resource intensive and time consuming.^6,7^

The assessment of ROB in the included RCTs is one of the key tasks undertaken by systematic review authors.^5,8,9^ The CLARITY group at McMaster University has created a modified version of the Cochrane ROB tool,^10^ which has been extensively applied in numerous high-quality systematic reviews. This tool facilitates the ROB assessment for the following 10 domains: random sequence generation; allocation concealment; blinding to patients, health care clinicians, data collectors, outcome assessors, and data analysts; and missing outcome data, selective outcome reporting, and other concerns. It classifies risk as definitely yes, probably yes, probably no, or definitely no, deliberately omitting the category of unclear.

LLMs^11,12^ have demonstrated exceptional capabilities in understanding and generating human-like text.^13^ Supported by advanced machine learning algorithms and vast datasets, these models have the potential to revolutionize the production of systematic reviews.^14^ By providing a dedicated prompt, LLMs can automatically conduct the assessment, which may reduce the time and resources required. To date, however, we know of no evidence confirming the capability of LLMs to undertake ROB assessments in systematic reviews. Therefore, this survey study aims to propose a structured prompt and evaluate the accuracy, consistency, and efficiency of using LLMs for assessing the ROB of RCTs.

## Methods

**Figure 1.**
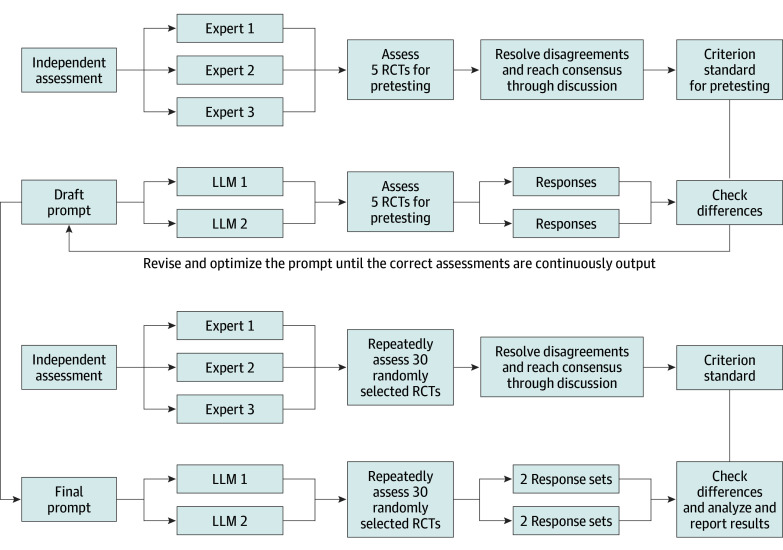
Flow Diagram of the Main Study Process LLM indicates large language model; RCT, randomized clinical trial.

This survey study was conducted between August 10, and October 30, 2023, adhering to the American Association for Public Opinion Research (AAPOR) reporting guideline.^15^ The medical ethics review committee of the School of Public Health at Lanzhou University deemed the study exempt from review and the requirement for informed consent, as all data originated from published research. We selected 2 highly representative LLMs for this study, ChatGPT (LLM 1) and Claude (LLM 2). Figure 1 shows the main study process.

### Formation of the Working Group

A multidisciplinary panel was assembled, including 3 senior experts in evidence-based medicine methodology (H.L., B.P., and L.G.), 2 computer scientists (H.L. and J.H.), and a research team of 5 investigators with backgrounds in using artificial intelligence in evidence-based medicine (H.L., M.S., Jiayi L., Jianing L., and W.Z.). The research team developed the prompt, conducted the study, recorded the results, and performed the statistical analysis. The 2 computer science experts refined and optimized the prompt. The 3 senior experts oversaw the assessment process and developed the criterion standard for assessment. All researchers completed a week-long training on systematic reviews to ensure a consistent understanding of the assessment process.

### Development of the Prompt

The assessment prompt development involved senior experts setting criteria based on guidelines (eAppendix 1 in Supplement 1),^10^ a researcher drafting a prompt for assessment, and piloting with 5 RCTs. Computer scientists reviewed the outputs, providing feedback for prompt refinement in iterations until it consistently matched the experts’ results. The finalized prompt (eAppendix 2 in Supplement 1) consisted of 3 parts: an introduction with setting the role,^16^ an instruction for the assessment, and a specification of the output format. The prompt provides clear instructions and typical examples for assessing each domain, thus guiding the LLMs to extract the corresponding information from the original text and make reasonable judgments, and then choose a rating from one of 4 options: definitely yes, probably yes, probably no, or definitely no. For example, when assessing random sequence generation, choose probably no if no details are provided. Opt for definitely yes for computer-generated randomness or traditional methods such as coin tossing. For sequences based on some rules, carefully choose between probably yes and probably no. If allocation relies on clinician judgment, participant preference, laboratory tests, or intervention availability, choose definitely no.

### Selection of Sample

First, we searched PubMed using the keywords *modified*, *Cochrane tool*, *risk of bias*, *CLARITY*, and *meta-analysis*. Records were screened in descending order of relevance until 3 meta-analyses using the modified Cochrane tool^10^ were selected. Subsequently, we sorted all the included RCTs in each systematic review in alphabetical order by the first author’s surname and publication year, assigning them numerical identifiers. Random numbers were generated using Excel version 2108 (Microsoft) to select 10 RCTs from each systematic review for our sample.

### Application of LLMs

For the assessment, the finalized prompt was applied to all RCTs using LLM 1 and LLM 2, with the access time spanning from September 30 to October 10, 2023. Whereas LLM 2 allows users to directly upload PDFs, uploading PDFs to LLM 1 required a separate plugin at the time the study was conducted, which we did not use to avoid bias. We first converted the PDF files into text documents to ensure the information fed to both models was identical. In assessing ROB for each RCT, the primary outcome was defined as either the outcome specified by the authors or, if it was not specified, the first reported outcome in the study. The outputs were accurately transcribed into a document (eAppendix 3 in Supplement 1 for LLM 1 and eAppendix 4 in Supplement 1 for LLM 2). Any assessment interrupted by technical issues was excluded and promptly redone. Each RCT was assessed twice with both LLMs, using the same prompt and ensuring consistent model versions. Throughout the process, strict adherence to the protocol was maintained to guarantee the fidelity of the assessment outcomes.

### Establishment of the Criterion Standard

The 3 senior experts independently assessed the RCTs using the criteria, then reconciling differences through consensus. This iterative discussion continued until consensus was achieved on each aspect of the ROB assessment for every RCT. These consensus-derived assessments formed the criterion standard (eTable 1 in Supplement 1), serving as a reference to gauge the precision of the ROB evaluations of the LLM tools.

### Statistical Analysis

Data analysis was conducted using R version 4.3.2 (R Project for Statistical Computing).^17^ All tests were 2-sided, with *P* < .05 considered statistically significant. For the ROB classification, responses indicating definitely yes or probably yes were categorized as low risk (negative outcome), while definitely no or probably no responses were categorized as high risk (positive outcome). True positives (TP) and true negatives (TN) were defined in accordance with the criterion standard, with deviations identified as false positives (FP) or false negatives (FN).

#### Accuracy

Accuracy of the LLMs in ROB assessment was quantified at the study-specific, domain-specific, and overall levels using the correct assessment rate, sensitivity, and specificity. For domain-specific accuracy, we further calculated the *F1* score, a harmonic mean of sensitivity and positive predictive value, and presented the proportion of high risk responses (TP plus FP) to assist the interpretability of the results.

*F1* = 2 × ([Positive Predictive Value × Sensitivity]/[Positive Predictive Value + Sensitivity])

Positive Predictive Value = TP/(TP + FP)

Risk differences (RD) with 95% CIs were calculated to provide a comparison of the correct assessment rate between LLM 1 and LLM 2 at each level.

#### Consistency

To evaluate the reliability of the LLMs’ repeated assessments, we used Cohen κ, which is derived from the proportion of observed agreement (*Po*) minus the actual concordance observed relative to the total number of observations (ie, the consistent assessment rate) as well as the proportion of expected agreement (*Pe*), which is the rate of agreement expected by chance based on the marginal probabilities.^18^ *Pe* is calculated from the positive (high risk) and negative assessments in the first (*P*_1_ and *N*_1_) and second (*P*_2_ and *N*_2_) evaluations. We used the thresholds presented in eTable 2 in Supplement 1 to interpret different values for κ.

*κ* = (*P_o_* − *P_e_*)/(1 − *P_e_*)

*P_o_* = (Number of Agreements on Positive + Number of Agreements on Negative)/Total Number of Assessments

*P_e_* = ([P_1_ × P_2_] + [N_1_ × N_2_])/(Total Number of Assessments)^2^

Given the potential homogeneity of risk assessments across domains, which could artificially elevate the expected agreement, we conducted a sensitivity analysis by calculating the prevalence-adjusted and bias-adjusted κ (PABAκ).^19^

*PABAκ* = 2*P_o_* = 1

Furthermore, RD was calculated to compare the difference in *Po* between LLM 1 and LLM 2.

#### Efficiency

The efficiency of the assessment process was measured by recording the time from text upload to completion of the full domain assessments. In this study, the network bandwidth supported upload and download speeds of approximately 100 megabits per second.

## Results

### Characteristics of the RCTs

We selected 1 meta-analysis published in 2021,^20^ 2 published in 2023,^21,22^ and 1 systematic review.^23^ The analyses focused on the associations between red meat intake and cardiometabolic and cancer outcomes, the efficacy and safety of type 2 diabetes treatments, and drug therapies for primary insomnia, respectively. We then randomly selected 30 RCTs^24,25,26,27,28,29,30,31,32,33,34,35,36,37,38,39,40,41,42,43,44,45,46,47,48,49,50,51,52,53^ included in these reviews. All selected trials were published in English. The trials were published between 1987 and 2022, with most (19 trials) published after 2013.

### Accuracy

**Figure 2.**
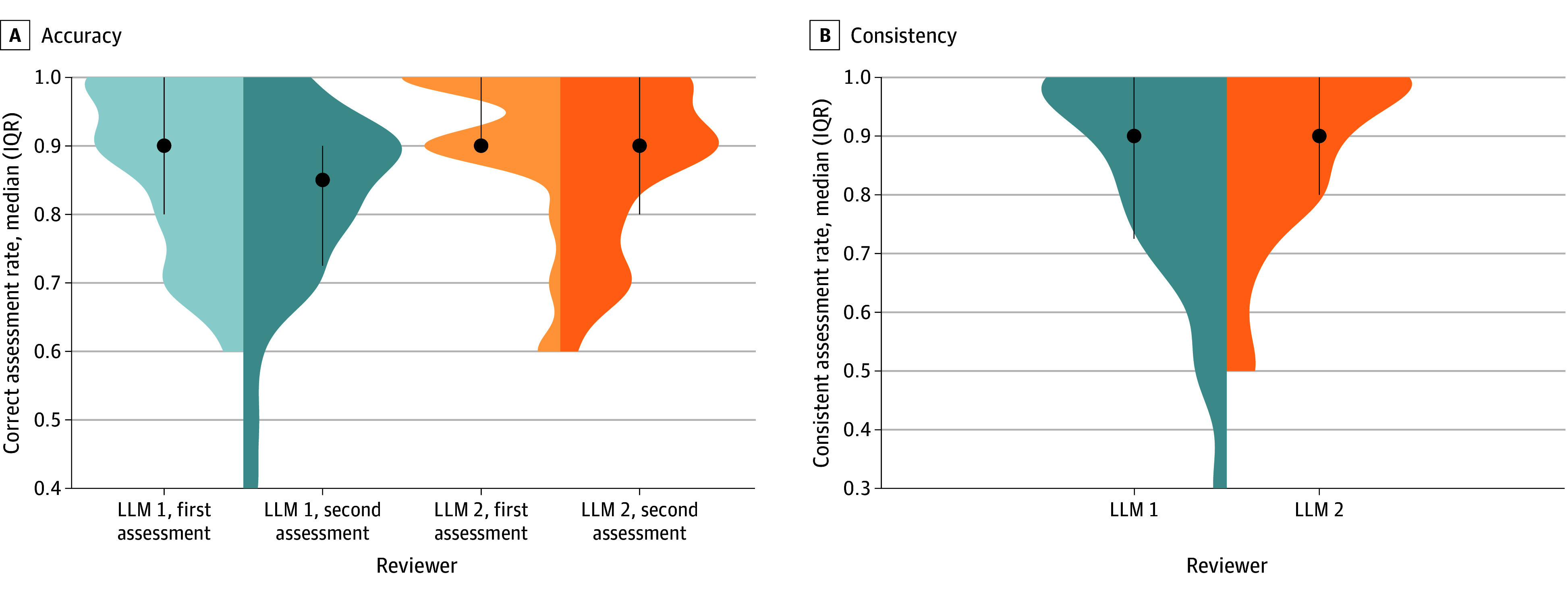
Comparison of the Overall Correct and Consistent Assessment Rates of Large Language Models (LLMs) 1 and 2 Across 2 Consecutive Assessments

The complete assessment results are summarized in eTable 3 and eTable 4 in Supplement 1. Both LLM 1 and LLM 2 demonstrated good accuracy (Table and Figure 2). LLM 1 achieved a mean correct assessment rate of 84.5% (95% CI, 81.5%-87.3%), and LLM 2 exhibited a marginally superior rate of 89.5% (95% CI, 87.0%-91.8%), with both displaying a median (IQR) correct overall assessment rate of 90.0% (80.0%-90.0% for LLM 1 and 90.0%-100.0% for LLM 2) across the 60 assessments of RCTs (2 assessments per each RCT) (Figure 2). LLM 2’s correct assessment rate was significantly higher compared with LLM 1 (RD, 0.05; 95% CI, 0.01-0.09; *P* = .01).

**Table.** Domain-Specific Accuracy and Consistency

Reviewer	Accuracy					Consistency	
	Correct assessment rate, %	Sensitivity	Specificity	*F1* score	Proportion of high risk responses, %	Cohen κ	Consistent assessment rate, %
LLM 1							
Domain 1	56.70	0.45	0.77	0.57	36.67	0.54	60.00
Domain 2	70.00	0.71	0.58	0.78	65.00	0.65	70.00
Domain 3.a	93.30	1.00	0.89	0.92	46.67	0.92	93.33
Domain 3.b	96.70	1.00	0.97	0.98	50.00	0.96	96.67
Domain 3.c	93.30	0.96	0.91	0.93	48.33	0.85	86.67
Domain 3.d	91.70	0.93	0.91	0.91	46.67	0.81	83.33
Domain 3.e	91.70	0.93	0.90	0.92	50.00	0.81	83.33
Domain 4	78.30	0.17	0.94	0.24	8.33	0.87	90.00
Domain 5	83.30	NA	0.83	NA	16.67	0.76	80.00
Domain 6	90.00	0.00	0.96	NA	3.33	0.91	93.33
LLM 2							
Domain 1	80.00	0.74	0.91	0.82	50.00	0.77	80.00
Domain 2	83.30	0.85	0.75	0.89	73.33	0.76	80.00
Domain 3.a	98.30	1.00	0.97	0.98	41.67	0.96	96.67
Domain 3.b	96.70	1.00	0.94	0.97	50.00	0.92	93.33
Domain 3.c	90.00	0.88	0.91	0.88	43.33	0.85	86.67
Domain 3.d	90.00	0.88	0.91	0.88	43.33	0.85	86.67
Domain 3.e	90.00	0.89	0.91	0.89	46.67	0.85	86.67
Domain 4	83.30	0.42	0.94	0.50	13.33	0.84	86.67
Domain 5	90.00	1.00	0.90	0.25	11.67	0.83	86.67
Domain 6	93.30	0.25	0.98	0.33	3.33	0.91	93.33

Abbreviations: LLM, large language model; NA, not available because the true positive number is equal to 0.

**Figure 3.**
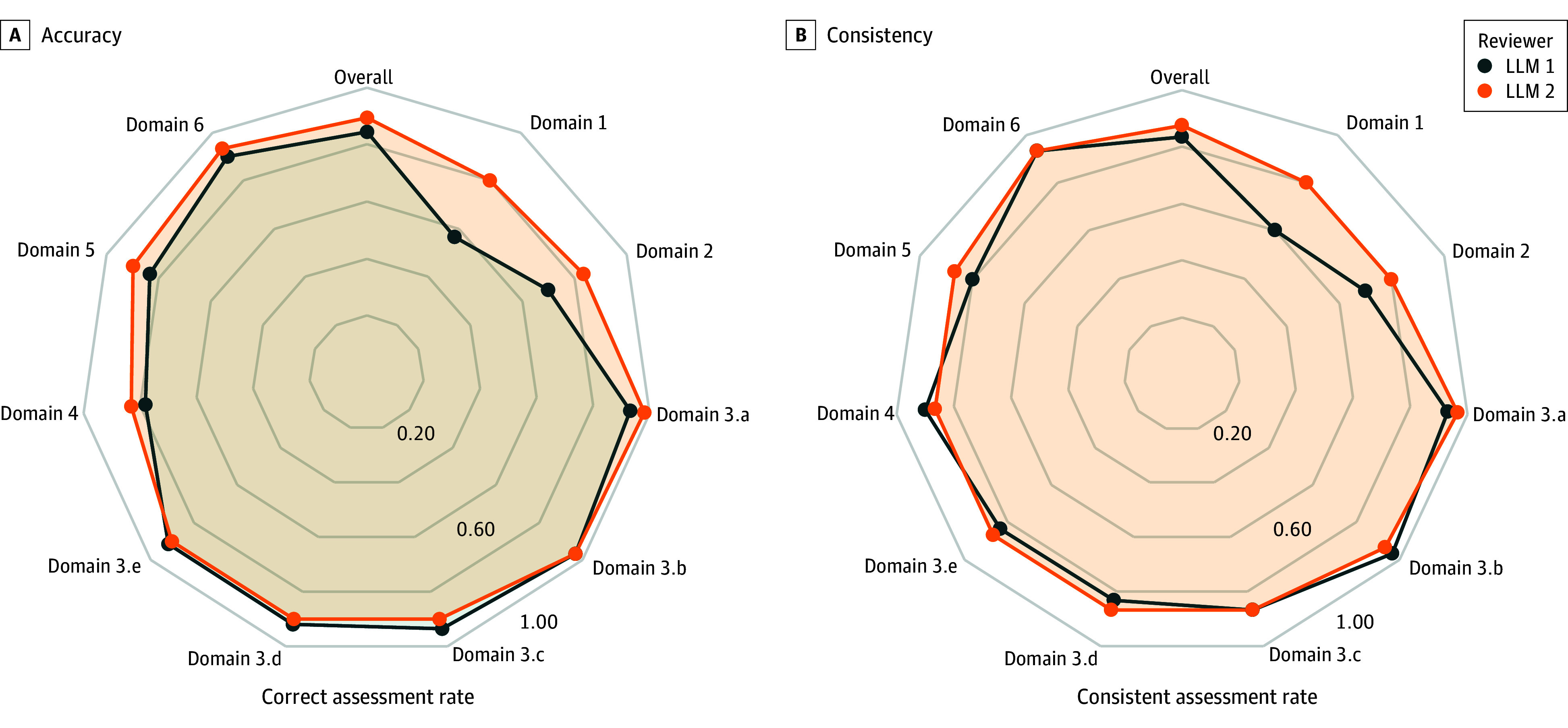
Correct and Consistent Assessment Rates of Large Language Models (LLMs) 1 and 2 for Each Domain

As depicted in Figure 3 and eTable 5 and eTable 6 in Supplement 1, the correct assessment rates were similar between the 2 LLMs across all 10 domains. LLM 1’s lowest correct assessment rate occurred in domain 1 (random sequence generation) at 56.7%, and was highest in domain 3.b (blinding to health care clinicians) at 96.7%. LLM 2’s correct assessment rate across the domains ranged from 80.0% in domain 1 to 98.3% in domain 3.a (blinding to patients). LLM 2 significantly outperformed LLM 1 in domain 1 (RD 0.23; 95% CI, 0.07-0.39; *P* = .01), with no significant difference in other domains. As presented in eTable 7 and eTable 8 in Supplement 1, of 60 assessments for each model, LLM 1 achieved 14 (23.3%) with full accuracy and 48 (80.0%) with 80% or higher accuracy, and LLM 2 had 24 perfect assessments (40.0%) and 48 (80.0%) with accuracy of 80% or more.

In analyzing the models’ outputs (eTable 9 in Supplement 1), of the overall 155 wrong assessments, 89 (57.4%) involved the models correctly identifying and articulating the appropriate rationale but making an erroneous judgment, while 66 (42.6%) were incorrect due to unrecognized or misidentified evidence. For 90 incorrect assessments in domains 1, 2 (allocation concealment), and 4 (missing outcome data), 68 of 90 (75.6%) were based on correct rationales but led to incorrect judgments. Of the 38 incorrect assessments in domain 1, 30 (78.9%) involved the models correctly stating the reason, such as “the study does not provide details on how the randomization sequence was generated.” Despite this, instead of selecting “probably no” as per the prompt’s guidance, they incorrectly judged it as “probably yes.” The presence of the keyword “random” in RCTs inclined the LLMs to conclude a low risk judgment. Domains 2 (allocation concealment) and 4 (missing outcome data) exhibit a similar cause for 72.4% and 73.9% of the wrong assessments, respectively.

LLM 1’s overall sensitivity was 0.82 (95% CI, 0.70-0.90) and specificity was 0.96 (95% CI, 0.89-0.99). For LLM 2, both the sensitivity (0.90; 95% CI, 0.81-0.95) and specificity (0.97; 95% CI, 0.91-0.95) were higher. The sensitivity of LLM 1 varied substantially across the domains, from only 0.17 and 0.00 in domains 4 and 6 (other concerns), respectively, to between 0.93 and 1.00 in domains 3.a to 3.e (blinding to allocated interventions). The specificity of LLM 1 was between 0.77 and 0.97 in all domains except domain 2, where it was considerably lower (0.58). The *F1* score was lowest in domains 1 and 4, at 0.57 and 0.24, respectively, which corresponded to the relatively low proportions of high risk responses (36.7% and 8.3%).

LLM 2’s sensitivity was lowest in domains 4 and 6 (0.42 and 0.25, respectively). Specificity ranged between 0.75 and 0.98. The *F1* score for LLM 2 was lowest in domains 4, 5, and 6, with values of 0.50, 0.25, and 0.33, respectively, which corresponded to the relatively low proportions of high risk responses (range, 3.3%-13.3%).

### Consistency

Both LLM 1 and LLM 2 showcased high overall consistent rates of 84.0% and 87.3%, respectively, without significant differences (RD, 0.03; 95% CI −0.02 to 0.08) (Figure 2). The κ was above 0.5 in all domains for both LLM 1 and LLM 2, indicating at least moderate agreement. For LLM 1, the κ surpassed 0.80 in 7 domains, while LLM 2 exceeded this threshold in 8 domains. The consistency in domains 1 and 2 was relatively lower for both models. eTable 10 in Supplement 1 lists the agreement across 4 assessments (twice per model) for each domain, of which only 13 studies (43.3%) had 4 consistent assessments in domain 1 (random sequence generation) and 14 studies (46.7%) in domain 2 (allocation concealment). The proportions of agreement in other domains were relatively high (range, 60%-90%).

On a study-specific level, both LLM 1 and LLM 2 produced identical results for 12 RCTs in repetitive assessments. As shown in eTable 11 and eTable 12 in Supplement 1, we found substantial or near perfect agreements in most assessments for both models, and the lowest consistent rate was 30% for LLM 1 and 50% for LLM 2. For all assessments (eTable 13 in Supplement 1), 15 of 30 studies achieved 80% or more proportions of agreement across all domains, with an average agreement of 70%.

### Efficiency

The duration of the conducted assessments for LLM 1 ranged between 52 and 127 seconds, with a mean of 77 seconds per assessment. For LLM 2, the mean duration was 53 seconds (range from 36 to 87 seconds).

## Discussion

In this survey study, we established a structured and feasible prompt that was capable of guiding LLMs in assessing the ROB in RCTs. The LLMs used in this study produced assessments that were very close to those of experienced human reviewers. Automated tools in systematic reviews exist but are underused due to difficult operation, poor user experience, and unreliable results.^20,54,55^ In contrast, both LLMs had high accessibility and user friendliness, demonstrating outstanding reliability and efficiency, thereby showing substantial potential for facilitating systematic review production.

Our study found that both LLMs demonstrated high accuracy and consistency, compared with human reviewers, in assessing the ROB of RCTs. LLM 2 had a significantly higher correct assessment rate compared with LLM 1. The potential causes for this discrepancy may stem from the different methods of submitting the articles. LLM 2 permits direct PDF file uploads, automatically converting them into text for analysis, while LLM 1 only allowed text (this was the case at the time of our study, but PDFs can be uploaded after the November 9, 2023, update). Thus, uploading PDFs requires additional plugins. Consequently, to maintain consistency, RCT articles were converted to text format before submission. However, the different text length constraints of the 2 LLMs required that multiple segments be uploaded sequentially for LLM 1, whereas LLM 2 processed the uploads in a single step. This could have potentially influenced LLM 1’s judgment and may explain the longer duration of assessment compared with LLM 2.

Both LLMs exhibited the lowest correct assessment rates in domain 1, concerning random sequence generation. Of the 38 incorrect assessments, 30 (78.95%) involved the models correctly stating the reason, such as “the study does not provide details on how the randomization sequence was generated.” Despite this, instead of selecting “probably no” as per the prompt’s guidance, they incorrectly judged it as “probably yes.” The presence of the keyword “random” in RCTs inclined the LLMs to conclude a low risk judgment. Domains 2 (allocation concealment) and 4 (missing outcome data) exhibit a similar cause for 72.4% and 73.9% of the wrong assessments, respectively. Due to explicit constraints set within the prompt, the reasons leading to these errors are not clear. However, since the models provided the correct rationale, researchers can easily identify the mistakes. Moreover, only 5 domains in 5 studies (from 2 studies in domain 1 and 3 studies in domain 4) were incorrectly rated by both models in 2 separate assessments, with the rationale being correct in each case. Given the convenience and speed of using LLMs, researchers are well equipped to perform ROB assessment on a broad range of studies. By conducting a series of assessments with 2 distinct LLMs and browsing the reasoning behind each, the majority of potential errors could be identified.

To our knowledge, this study is the first to transparently explore the feasibility of applying LLMs to the assessment of ROB in RCTs. The study addressed multiple aspects of the feasibility of LLM use, including accuracy, consistency, and efficiency. A detailed and structured prompt was proposed and performed commendably in practical application. Our findings preliminarily suggest that with an appropriate prompt, LLM 1 and LLM 2 can be used alongside the modified Cochrane tool to assess the ROB of RCTs accurately and efficiently.

### Limitations

This survey study has several limitations. First, given the low probability of positive assessments in certain domains, a large sample would be required to draw robust conclusions. However, due to usage restrictions pertaining to LLM 1 and LLM 2, our study was conducted with a constrained sample size. Second, all RCTs assessed were in English; thus, the efficacy of this method for literature in other languages remains unclear. Third, the criterion standard for this research was determined by consensus among 3 senior experts. The prompt provided to the LLMs for processing different RCTs were uniform, meaning that the criterion standard was established from the broadest and most generic perspective. Additionally, in maintaining consistency with the information uploaded to the LLMs, the determination of the criterion standard did not consider supplementary materials such as appendices and registration details. Because it is challenging for artificial intelligence to interpret lengthy appendices, LLMs may not be capable of completing the task independently when additional information is imperative for accurate assessment. However, this constraint could be mitigated in the future by permitting LLMs access links to external sources. Since this functionality was still available as a beta testing version only during our study, we did not use it. Fourth, the study conducted assessments only on the primary outcome; however, responses indicate that LLMs might have the capability to simultaneously assess all reported outcomes. In practice, the prompt could be tailored to guide the assessment toward specific outcomes.

## Conclusions

In this survey study of the application of LLMs to the assessment of ROB in RCTs, we found that LLM 1 and LLM 2 achieved commendable accuracy and consistency when directed by a structured prompt. By scrutinizing the rationale provided and comparing multiple assessments across different models, researchers were able to efficiently identify and correct nearly all errors.

## Notes

Supplement 1. **eAppendix 1.** Introduction for assessing risk of bias using the modified Cochrane tool **eAppendix 2.** Prompt for LLM 1 and LLM 2 to assess risk of bias in randomized clinical trials with the modified Cochrane tool **eAppendix 3.** Responses from LLM 1 **eAppendix 4.** Responses from LLM 2 **eTable 1.** The criterion standard response for each randomized clinical trial and domain **eTable 2.** Thresholds to interpret the values for Cohen's κ **eTable 3.** The results of assessment by LLM 1 for each domain and RCT **eTable 4.** The results of assessment by LLM 2 for each RCT and domain **eTable 5.** The domain-specific accuracy and consistency of assessments by LLM 1 **eTable 6.** The domain-specific accuracy and consistency of assessments by LLM 2 **eTable 7.** The study-specific accuracy of assessments by LLM 1 **eTable 8.** The study-specific accuracy of assessments by LLM 2 **eTable 9.** The reason and example of wrong assessments for each domain **eTable 10.** The domain-specific consistency between 4 assessments by both LLM 1 and LLM 2 **eTable 11.** The study-specific consistency between 2 assessments by LLM 1 **eTable 12.** The study-specific consistency between 2 assessments by LLM 2 **eTable 13.** The study-specific consistency between 4 assessments by both LLM 1 and LLM 2

Supplement 2.
Data Sharing Statement
