# Supplementary material for: Assessing the Risk of Bias in Randomized Clinical Trials With Large Language Models
Source: JAMA Netw Open. 2024 May 22;7(5):e2412687. doi: 10.1001/jamanetworkopen.2024.12687 (PMC11112444; doi:10.1001/jamanetworkopen.2024.12687)
Supplement: Supplement 1. — eAppendix 1. Introduction for assessing risk of bias using the modified Cochrane tool eAppendix 2. Prompt for LLM 1 and LLM 2 to assess risk of bias in randomized clinical trials with the modified Cochrane tool eAppendix 3. Responses from LLM 1 eAppendix 4. Responses from LLM 2 eTable 1. The criterion standard response for each randomized clinical trial and domain eTable 2. Thresholds to interpret the values for Cohen's κ eTable 3. The results of assessment by LLM 1 for each domain and RCT eTable 4. The results of assessment by LLM 2 for each RCT and domain eTable 5. The domain-specific accuracy and consistency of assessments by LLM 1 eTable 6. The domain-specific accuracy and consistency of assessments by LLM 2 eTable 7. The study-specific accuracy of assessments by LLM 1 eTable 8. The study-specific accuracy of assessments by LLM 2 eTable 9. The reason and example of wrong assessments for each domain eTable 10. The domain-specific consistency between 4 assessments by both LLM 1 and LLM 2 eTable 11. The study-specific consistency between 2 assessments by LLM 1 eTable 12. The study-specific consistency between 2 assessments by LLM 2 eTable 13. The study-specific consistency between 4 assessments by both LLM 1 and LLM 2 [file jamanetwopen-e2412687-s001.pdf]

Supplemental Online Content

Lai H, Ge L, Sun M, et al. Assessing the risk of bias in randomized clinical trials with large language models. *JAMA Netw Open*. 2024;7(5):e2412687. doi:10.1001/jamanetworkopen.2024.12687

eAppendix 1. Introduction for assessing risk of bias using the modified Cochrane tool
eAppendix 2. Prompt for LLM1 and LLM2 to assess risk of bias in randomized clinical trials with the modified Cochrane tool
eAppendix 3. Responses from LLM1
eAppendix 4. Responses from LLM2
eTable 1. The criterion standard response for each randomized clinical trial and domain
eTable 2. Thresholds to interpret the values for Cohen κ
eTable 3. The results of assessment by LLM1 for each domain and RCT
eTable 4. The results of assessment by LLM2for each RCT and domain
eTable 5. The domain-specific accuracy and consistency of assessments by LLM1
eTable 6. The domain-specific accuracy and consistency of assessments by LLM2
eTable 7. The study-specific accuracy of assessments by LLM1
eTable 8. The study-specific accuracy of assessments by LLM2
eTable 9. The reason and example of wrong assessments for each domain
eTable 10. The domain-specific consistency between 4 assessments by both LLM1 and LLM2
eTable 11. The study-specific consistency between 2 assessments by LLM1
eTable 12. The study-specific consistency between 2 assessments by LLM2
eTable 13. The study-specific consistency between 4 assessments by both LLM1 and LLM2

This supplemental material has been provided by the authors to give readers additional information about their work.

© 2024 Lai H et al. *JAMA Network Open.*

eAppendix 1. Introduction for Assessing Risk of Bias Using the Modified Cochrane Tool

The following instructions are based on guidance provided by the CLARITY group (https://www.distillersr.com/resources/methodological-resources/tool-to-assess-risk-of-bias-in-randomized-controlled-trials-distillersr):

The reviewer should answer each of the following question as "definitely yes", "probably yes", "probably no", or "definitely no".

1) Was the allocation sequence adequately generated?

a) Examples of low risk of bias:

i. Referring to a random number table

ii. Using a computer random number generator

iii. Coin tossing

iv. Shuffling cards or envelopes

v. Throwing dice

vi. Drawing of lots

vii. Minimization with or without a random element

b) Examples of high risk of bias:

i. Sequence generated by odd or even date of birth

ii. Sequence generated by some rule based on date (or day) of admission

iii. Sequence generated by some rule based on hospital or clinic record number

iv. Allocation by judgement of the clinician

v. Allocation by preference of the participant

vi. Allocation based on the results of a series laboratory test or series of tests

vii. Allocation by availability of the intervention

2) Was the allocation adequately concealed?

a) Examples of low risk of bias allocation concealment techniques:

i. Central allocation (including telephone, web-based, and pharmacy-controlled, randomization)

b) Examples of possible low risk of bias:

© 2024 Lai H et al. *JAMA Network Open.*

i. Sequentially numbered drug containers of identical appearance

ii. Sequentially numbered, opaque, sealed envelopes

c) Examples of high risk of bias allocation concealment techniques:

i. Using an open random allocation schedule (e.g. a list of random numbers)

ii. Assignment envelopes were used without appropriate safeguards (e.g. if envelopes were unsealed or non-opaque or not sequentially numbered)

iii. Alternation or rotation

iv. Date of birth

v. Case record number

vi. Any other explicitly unconcealed procedure

3) Blinding: Was knowledge of the allocated interventions adequately prevented?

3.a. Were patients blinded?

3.b. Were healthcare providers blinded?

3.c. Were data collectors blinded?

3.d. Were outcome assessors blinded?

3.e. Were data analysts blinded?

a) Examples of low risk of bias:

i. No blinding but the review authors judge that the outcome and the outcome measurement are not likely influenced by lack of blinding

ii. Blinding of participants and key study personnel ensured, and unlikely that blinding could have been broken

iii. Either participants or some key study personnel were not blinded, but outcome assessment was blinded and the nonblinding of others unlikely to introduce bias

b) Examples of high risk of bias:

i. No blinding but the review authors judge that the outcome and the outcome measurement are not likely influenced by lack of blinding

ii. Blinding of participants and key study personnel ensured, and unlikely that blinding could have been broken

iii. Either participants or some key study personnel were not blinded, but outcome assessment was blinded and the nonblinding of others unlikely to introduce bias

© 2024 Lai H et al. *JAMA Network Open.*

4) Was loss to follow-up (missing outcome data) infrequent?

a) Examples of low risk of bias:

i. No missing outcome data

ii. Reasons for missing outcome data unlikely to be related to outcome (for survival data, censoring unlikely to be introducing bias)

iii. Missing outcome data balanced in numbers across intervention groups, with similar reasons for missing data across groups

iv. For dichotomous outcome data, the proportion of missing outcomes compared with observed event risk not enough to have an important impact on the intervention effect estimate

v. For continuous outcome data, plausible effect size (difference in means or standardized difference in means) among missing outcomes not enough to have an important impact on observed effect size

vi. Missing data have been imputed using appropriate methods

b) Examples of high risk of bias:

i. Reason for missing outcome data likely to be related to true outcome, with either imbalance in numbers or reasons for missing data across intervention groups

ii. For dichotomous outcome data, the proportion of missing outcomes compared with observed event risk enough to induce important bias in intervention effect estimate

iii. For continuous outcome data, plausible effect size (difference in means or standardized difference in means) among missing outcomes enough to induce clinically relevant bias in observed effect size

iv. "As-treated" analysis done with substantial departure of the intervention received from that assigned at randomization

v. Potentially inappropriate application of simple imputation

5) Are reports of the study free of selective outcome reporting?

a) Examples of low risk of bias:

i. The study protocol is available and all of the study"s pre-specified (primary and secondary) outcomes that are of interest in the review have been reported in the pre-specified way

ii. The study protocol is not available but it is clear that the published reports include all expected outcomes, including those that were pre-specified (convincing text of this nature may be uncommon)

b) Examples of high risk of bias:

i. Not all of the study"s pre-specified primary outcomes have been reported

© 2024 Lai H et al. *JAMA Network Open.*

ii. One or more primary outcomes is reported using measurements, analysis methods or subsets of the data (e.g. subscales) that were not pre-specified

iii. One or more reported primary outcomes were not pre-specified (unless clear justification for their reporting is provided, such as an unexpected adverse effect)

iv. One or more outcomes of interest in the review are reported incompletely so that they cannot be entered in a meta-analysis

v. The study report fails to include results for a key outcome that would be expected to have been reported for such a study

6) Was the study apparently free of other problems that could put it at a risk of bias?

a) Examples of low risk of bias:

i. The study appears to be free of other sources of bias

b) Examples of high risk of bias:

i. Had a potential source of bias related to the specific study design used

ii. Stopped early due to some data-dependent process (including a formal-stopping rule)

iii. Had extreme baseline imbalance

iv. Has been claimed to have been fraudulent

v. Had some other problem

© 2024 Lai H et al. *JAMA Network Open.*

eAppendix 2. Prompt for LLM1 and LLM2 to Assess Risk of Bias in Randomized Clinical Trials With the Modified Cochrane Tool

Introduction and Role Setting:

You are a professional reviewer. You are particularly good at learning evaluation criteria, and closely following it to assess the risk of bias of Randomized Controlled Trials (RCTs). You can fully understand and follow the evaluation guidelines and evaluate the RCTs I have provided to you. Make sure all your judgments are based on the facts reported in the article and not on any extrapolation or speculation of your own. Finally, make sure your answers are completely correct.

Guidelines for Evaluation:

Note: The examples provided in the tool are illustrative and do not cover all possible scenarios in real-world applications. Use your expert judgment to evaluate each item based on the information provided in the RCT, and do not rely solely on the examples.

Important:

• The evaluation should be conducted only for one primary outcome.

• If there is too little information to support the judgment, do not speculate positively.

1. Was the allocation sequence adequately generated? Evaluate the adequacy of the allocation sequence generation based on the information provided in the RCT, considering the following criteria:

o The most important: If no statements are provided on how the randomization sequence was generated, select "Probably no", even if randomization is mentioned.

o If computer-generated random numbers, coin tossing, card or envelope shuffling, dice rolling, lot drawing, or minimization (with or without a random element) were used, select "Definitely yes."

o If the sequence was generated based on the odd or even date of birth, some rule based on the date (or day) of admission, or some rule based on hospital or clinic record number, carefully evaluate and choose between "Probably yes" and "Probably no."

o If allocation was based on clinician judgment, participant preference, results of a series of laboratory tests, or availability of the intervention, select "Definitely no."

2. Was the allocation adequately concealed? Evaluate the adequacy of allocation concealment based on the information provided in the RCT, considering the following criteria:

© 2024 Lai H et al. *JAMA Network Open.*

o If central allocation (including telephone, web-based, and pharmacy-controlled randomization), sequentially numbered drug containers of identical appearance, or sequentially numbered, opaque, sealed envelopes were used, select “Definitely yes.”

o If an open random allocation schedule was used or if assignment envelopes were used without appropriate safeguards (e.g., if envelopes were unsealed, non-opaque, or not sequentially numbered), select “Definitely no.”

o If no statements are provided on allocation concealment, select “Probably no.”

3. Blinding: Was knowledge of the allocated interventions adequately prevented? Evaluate the adequacy of blinding for each of the following, based on the information provided in the RCT:

3.a. Were patients blinded?

3.b. Were healthcare providers blinded?

3.c. Were data collectors blinded?

3.d. Were outcome assessors blinded?

3.e. Were data analysts blinded?

o For 3.a. to 3.e., follow these standards.

o If no blinding but you judge that the outcome and the outcome measurement are not likely influenced by lack of blinding, select “Probably yes.”

o If blinding of participants and key study personnel ensured, and unlikely that blinding could have been broken, select “Probably yes.”

o If either participants or some key study personnel were not blinded, but outcome assessment was blinded and the nonblinding of others unlikely to introduce bias, select “Probably yes.”

o If no blinding or incomplete blinding, and the outcome or outcome measurement is likely to be influenced by lack of blinding, select “Probably no.”

o If blinding of key study participants and personnel attempted, but likely that the blinding could have been broken, select “Probably no.”

o If either participants or some key study personnel were not blinded, and the nonblinding of others likely to introduce bias, select “Probably no.”

4. Was loss to follow-up (missing outcome data) infrequent? Evaluate the frequency of loss to follow-up based on the information provided in the RCT, considering the following criteria:

© 2024 Lai H et al. *JAMA Network Open.*

o If there are no missing outcome data, or the reasons for missing outcome data are unlikely to be related to the outcome, select “Definitely yes.”

o If missing outcome data are balanced across intervention groups, with similar reasons for missing data across groups, select “Probably yes.”

o If the proportion of missing outcomes is enough to have an important impact on the intervention effect estimate, select “Definitely no.”

o If the follow-up rate at the longest time point is greater than 90%, i.e. more than 90% of participants completed the trial, or the dropout rate at the longest time point is less than 10%, you can generally consider selecting “Definitely yes.”

o If the follow-up rate is between 80% and 90%, i.e. more than 80% but less than 90% of participants completed the trial, or the dropout rate at the longest time point is between 10% and 20%, consider selecting “Probably yes.”

o If the follow-up rate is below 80%, i.e. less than 80% of participants completed the trial, or the dropout rate is greater than 20%, consider selecting “Definitely no.”

o If no statements are available to support the assessment, select “Probably no.”

5. Are reports of the study free of selective outcome reporting? Evaluate the presence of selective outcome reporting based on the information provided in the RCT, considering the following criteria:

o If the study protocol is available and all of the study’s pre-specified (primary and secondary) outcomes of interest in the review have been reported in the pre-specified way, select “Definitely yes.”

o If the study protocol is not available but it is clear that the published reports include all expected outcomes, including those that were pre-specified, select “Probably yes.”

o If not all of the study’s pre-specified primary outcomes have been reported, or if one or more reported primary outcomes were not pre-specified, select “Definitely no.”

o Don't select “Probably no” just because the study protocol is not available

6. Was the study apparently free of other problems that could put it at a risk of bias? Evaluate the presence of other potential sources of bias based on the information provided in the RCT, considering the following criteria:

o If the study appears to be free of other sources of bias, select “Definitely yes.”

o If the study had a potential source of bias related to the specific study design used, or had some other problem that could put it at risk of bias, select “Probably no” or “Definitely no.”

© 2024 Lai H et al. *JAMA Network Open.*

Output Format:

For each reported outcome in the RCT, provide the evaluation results in the following format:

Article ID: [Insert First Author's Last Name], [Insert Year of Publication]

Outcome Name: [Insert Outcome Name]

1. Item name (e.g., "1. Was the allocation sequence adequately generated?")

o Response (e.g., "Definitely yes")

o Reason you summarized (e.g., "No statements are available on how the randomization sequence was generated.""258/316 (82%) participants completed 26 weeks of treatment. The dropout rate is less than 20% in all groups.")

2. [Next Item Name]

o Response

o Reason

(Continue in the same format for all items)

Ensure there is a clear separation between the sets of responses for different outcomes, and maintain consistency in the format for each outcome.

Before you output the answer, please make sure that any evaluation results are based on my request. Please re-check: In the first item, if no statements are available on how the randomization sequence was generated, select "Probably no", even if randomization is mentioned. In each item, if no statements are available to support the assessment, select “Probably no.” But be careful.

© 2024 Lai H et al. *JAMA Network Open.*

eAppendix 3. Responses From LLM1

ID: Benassi-Evans, 2009

First response:

Thank you for providing the detailed guidelines and examples for evaluating the risk of bias of randomized controlled trials (RCTs). I have carefully reviewed the criteria you outlined and will follow them closely when assessing the RCT article you attached. Please find my evaluation below:

Benassi-Evans et al., 2009

Outcome Name: Weight

1. Was the allocation sequence adequately generated?

    - Probably no

    - The method of randomization is not described.

2. Was the allocation adequately concealed?

    - Probably no

    - Details on allocation concealment are not provided.

3. Blinding: Was knowledge of the allocated interventions adequately prevented?

    3a. Were patients blinded?

        - Probably no

        - No details provided on blinding of patients

    3b. Were healthcare providers blinded?

        - Probably no

        - No details provided on blinding of healthcare providers

    3c. Were data collectors blinded?

        - Probably no

        - No details provided on blinding of data collectors

© 2024 Lai H et al. *JAMA Network Open.*

3d. Were outcome assessors blinded?

- Probably no

- No details provided on blinding of outcome assessors

3e. Were data analysts blinded?

- Probably no

- No details provided on blinding of data analysts

4. Was loss to follow-up (missing outcome data) infrequent?

- Probably yes

- Although follow-up rates are not explicitly reported, the number of subjects analyzed seems to be the same at baseline and endline based on the figures, suggesting low dropout.

5. Are reports of the study free of selective outcome reporting?

- Probably yes

- The outcome of micronucleus frequency seems to be reported as would be expected for the study purpose and design.

6. Was the study apparently free of other problems that could put it at a risk of bias?

- Probably yes

- No other apparent sources of bias.

Outcome Name: Frequency of nucleoplasmic bridges

1. Was the allocation sequence adequately generated?

- Probably no

- The method of randomization is not described.

2. Was the allocation adequately concealed?

- Probably no

- Details on allocation concealment are not provided.

© 2024 Lai H et al. *JAMA Network Open.*

3. Blinding: Was knowledge of the allocated interventions adequately prevented?

3a. Were patients blinded?

- Probably no
- No details provided on blinding of patients

3b. Were healthcare providers blinded?

- Probably no
- No details provided on blinding of healthcare providers

3c. Were data collectors blinded?

- Probably no
- No details provided on blinding of data collectors

3d. Were outcome assessors blinded?

- Probably no
- No details provided on blinding of outcome assessors

3e. Were data analysts blinded?

- Probably no
- No details provided on blinding of data analysts

4. Was loss to follow-up (missing outcome data) infrequent?

- Probably yes
- Although follow-up rates are not explicitly reported, the number of subjects analyzed seems to be the same at baseline and endline based on the figures, suggesting low dropout.

5. Are reports of the study free of selective outcome reporting?

- Probably yes
- The outcome of frequency of nucleoplasmic bridges seems to be reported as would be expected for the study purpose and design.

6. Was the study apparently free of other problems that could put it at a risk of bias?

- Probably yes

© 2024 Lai H et al. *JAMA Network Open.*

- No other apparent sources of bias.

Please let me know if I have followed the guidelines correctly and provided the appropriate level of detail in my assessment. I'm happy to clarify or expand on any part of my evaluation.

© 2024 Lai H et al. *JAMA Network Open.*

ID: Benassi-Evans, 2009

Second response:

Thank you for providing the detailed guidelines and examples for evaluating randomized controlled trials (RCTs). I have carefully reviewed the criteria and will follow them closely when evaluating the RCT article you have attached.

For the article "High protein–high red meat versus high carbohydrate weight loss diets do not differ in effect on genome stability and cell death in lymphocytes of overweight men" by Benassi-Evans et al. 2009, I will provide my assessment for the following outcome:

Article ID: Benassi-Evans, 2009

Outcome Name: Frequency of micronuclei in binucleated cells

1. Was the allocation sequence adequately generated?

   - Probably no

   - The article does not provide details on how the randomization sequence was generated.

2. Was the allocation adequately concealed?

   - Probably no

   - The article does not provide details on allocation concealment.

3. Blinding: Was knowledge of the allocated interventions adequately prevented?

   3a. Were patients blinded?

      - Probably no

      - The article does not provide details on blinding of patients.

   3b. Were healthcare providers blinded?

      - Probably no

      - The article does not provide details on blinding of healthcare providers.

   3c. Were data collectors blinded?

© 2024 Lai H et al. *JAMA Network Open.*

- Probably no

- The article does not provide details on blinding of data collectors.

3d. Were outcome assessors blinded?

- Probably no

- The article does not provide details on blinding of outcome assessors.

3e. Were data analysts blinded?

- Probably no

- The article does not provide details on blinding of data analysts.

4. Was loss to follow-up (missing outcome data) infrequent?

- Probably no

- The article does not provide details to assess frequency of missing outcome data.

5. Are reports of the study free of selective outcome reporting?

- Probably no

- The study protocol is not available to compare with the outcomes reported.

6. Was the study apparently free of other problems that could put it at a risk of bias?

- Probably no

- The lack of details on randomization, allocation concealment, blinding, and missing data could put the study at risk of bias.

Please let me know if you would like me to evaluate any other outcomes from this article. I will be happy to follow the guidelines closely for any additional assessments.

© 2024 Lai H et al. *JAMA Network Open.*

ID: Davis, 2017

First response:

Here are the risk of bias assessments for the article "A Mediterranean diet lowers blood pressure and improves endothelial function: results from the MedLey randomized intervention trial" by Davis et al. (2017):

Article ID: Davis, 2017

Outcome Name: Systolic blood pressure

1. Was the allocation sequence adequately generated?

    - Probably yes

    - The article states "Minimization was done by an investigator who was not involved in the data collection after enrollment." Minimization involves some random element, so this probably constitutes adequate sequence generation.

2. Was the allocation adequately concealed?

    - Probably no

    - No details are provided on allocation concealment.

3. Blinding: Was knowledge of the allocated interventions adequately prevented?

3a. Were patients blinded?

    - Probably no

    - No details provided on blinding of patients.

3b. Were healthcare providers blinded?

    - Probably no

    - No details provided on blinding of healthcare providers.

3c. Were data collectors blinded?

    - Probably no

    - No details provided on blinding of data collectors.

© 2024 Lai H et al. *JAMA Network Open.*

3d. Were outcome assessors blinded?

- Probably no

- No details provided on blinding of outcome assessors.

3e. Were data analysts blinded?

- Probably no

- No details provided on blinding of data analysts.

4. Was loss to follow-up (missing outcome data) infrequent?

- Probably yes

- 10% of participants withdrew after study commencement. Reasons appeared to be unrelated to outcomes.

5. Are reports of the study free of selective outcome reporting?

- Probably yes

- The trial was registered prospectively and reported on the pre-specified outcomes.

6. Was the study apparently free of other problems that could put it at a risk of bias?

- Probably yes

- No other apparent sources of bias.

Outcome Name: Flow-mediated dilatation

1. Was the allocation sequence adequately generated?

- Probably yes

- The article states "Minimization was done by an investigator who was not involved in the data collection after enrollment." Minimization involves some random element, so this probably constitutes adequate sequence generation.

2. Was the allocation adequately concealed?

© 2024 Lai H et al. *JAMA Network Open.*

- Probably no

- No details provided on allocation concealment.

3. Blinding: Was knowledge of the allocated interventions adequately prevented?

3a. Were patients blinded?

- Probably no

- No details provided on blinding of patients.

3b. Were healthcare providers blinded?

- Probably no

- No details provided on blinding of healthcare providers.

3c. Were data collectors blinded?

- Probably no

- No details provided on blinding of data collectors.

3d. Were outcome assessors blinded?

- Probably no

- The article states "It was not possible to blind the analysis because the image files contained participant identification information."

3e. Were data analysts blinded?

- Probably no

- The article states "It was not possible to blind the analysis because the image files contained participant identification information."

4. Was loss to follow-up (missing outcome data) infrequent?

- Probably yes

- Only 4 additional participants withdrawn from analysis of this outcome.

5. Are reports of the study free of selective outcome reporting?

- Probably yes

- The trial was registered prospectively and reported on the pre-specified outcomes.

© 2024 Lai H et al. *JAMA Network Open.*

6. Was the study apparently free of other problems that could put it at a risk of bias?

- Probably yes
- No other apparent sources of bias.

© 2024 Lai H et al. *JAMA Network Open.*

ID: Davis, 2017

Second response:

Here are the risk of bias assessments for the provided RCT article:

Article ID: Davis et al., 2017

Outcome Name: Systolic blood pressure

1. Was the allocation sequence adequately generated?

  - Probably no

  - The article does not provide details on how the randomization sequence was generated.

2. Was the allocation adequately concealed?

  - Probably no

  - The article does not provide details on allocation concealment.

3. Blinding: Was knowledge of the allocated interventions adequately prevented?

3a. Were patients blinded?

  - Probably no

  - The article does not provide details on blinding of patients.

3b. Were healthcare providers blinded?

  - Probably no

  - The article does not provide details on blinding of healthcare providers.

3c. Were data collectors blinded?

  - Probably no

  - The article does not provide details on blinding of data collectors.

© 2024 Lai H et al. *JAMA Network Open.*

3d. Were outcome assessors blinded?

- Probably no

- The article does not provide details on blinding of outcome assessors.

3e. Were data analysts blinded?

- Probably no

- The article does not provide details on blinding of data analysts.

4. Was loss to follow-up (missing outcome data) infrequent?

- Probably no

- The article does not provide details to assess frequency of missing outcome data.

5. Are reports of the study free of selective outcome reporting?

- Probably no

- The study protocol is not available to compare with the published report.

6. Was the study apparently free of other problems that could put it at a risk of bias?

- Probably no

- The article does not provide sufficient details to assess other potential sources of bias.

Outcome Name: Flow-mediated dilatation

1. Was the allocation sequence adequately generated?

- Probably no

- The article does not provide details on how the randomization sequence was generated.

2. Was the allocation adequately concealed?

- Probably no

- The article does not provide details on allocation concealment.

© 2024 Lai H et al. *JAMA Network Open.*

3. Blinding: Was knowledge of the allocated interventions adequately prevented?

3a. Were patients blinded?

- Probably no
- The article does not provide details on blinding of patients.

3b. Were healthcare providers blinded?

- Probably no
- The article does not provide details on blinding of healthcare providers.

3c. Were data collectors blinded?

- Probably no
- The article does not provide details on blinding of data collectors.

3d. Were outcome assessors blinded?

- Probably no
- The article does not provide details on blinding of outcome assessors.

3e. Were data analysts blinded?

- Probably no
- The article does not provide details on blinding of data analysts.

4. Was loss to follow-up (missing outcome data) infrequent?

- Probably no
- The article does not provide details to assess frequency of missing outcome data.

5. Are reports of the study free of selective outcome reporting?

- Probably no
- The study protocol is not available to compare with the published report.

© 2024 Lai H et al. *JAMA Network Open.*

6. Was the study apparently free of other problems that could put it at a risk of bias?

- Probably no
- The article does not provide sufficient details to assess other potential sources of bias.

© 2024 Lai H et al. *JAMA Network Open.*

ID: de Mello, 2008

First response:

Here is the risk of bias assessment for the blood pressure outcome reported in the provided RCT by de Mello et al. 2008:

Article ID: de Mello, 2008

Outcome Name: UAER

1. Was the allocation sequence adequately generated?

   - Probably yes

   - The method of allocation is described as: "The randomization process was blind and was performed in groups of four. In each group, patients were asked to draw one among four slips of paper, with two indicating inclusion in the enalapril group, and two indicating inclusion in the CD group." While this suggests some random component, drawing slips of paper may not be adequately random.

2. Was the allocation adequately concealed?

   - Probably no

   - The method of allocation concealment is not described.

3. Blinding: Was knowledge of the allocated interventions adequately prevented?

   3a. Were patients blinded?

      - Probably no

      - The study is described as open-label.

   3b. Were healthcare providers blinded?

      - Definitely no

      - The study is described as open-label.

© 2024 Lai H et al. *JAMA Network Open.*

3c. Were data collectors blinded?

- Probably no
- Blinding of data collectors is not described.

3d. Were outcome assessors blinded?

- Probably no
- Blinding of outcome assessors is not described.

3e. Were data analysts blinded?

- Probably no
- Blinding of data analysts is not described.

4. Was loss to follow-up (missing outcome data) infrequent?

- Probably yes
- 4 patients out of 32 randomized did not complete the study. This dropout rate appears relatively low.

5. Are reports of the study free of selective outcome reporting?

- Probably yes
- The trial was not registered, but it appears that the pre-specified outcomes are reported.

6. Was the study apparently free of other problems that could put it at risk of bias?

- Probably yes
- No other apparent sources of bias.

© 2024 Lai H et al. *JAMA Network Open.*

ID: de Mello, 2008

Second response:

Here is the risk of bias assessment for the blood pressure outcome reported in the provided RCT by de Mello et al. 2008:

Article ID: de Mello, 2008

Outcome Name: UAER

1. Was the allocation sequence adequately generated?

    - Probably yes

    - The method of allocation is described as: "The randomization process was blind and was performed in groups of four. In each group, patients were asked to draw one among four slips of paper, with two indicating inclusion in the enalapril group, and two indicating inclusion in the CD group." This suggests some form of randomization, though the exact process is not specified.

2. Was the allocation adequately concealed?

    - Probably no

    - The method of allocation concealment is not described. The fact that patients drew slips assigning them to study groups suggests allocation was likely not adequately concealed.

3. Blinding: Was knowledge of the allocated interventions adequately prevented?

    3a. Were patients blinded?

        - Definitely no

        - The study is described as an "open-label" trial, indicating that patients were aware of their treatment assignment.

    3b. Were healthcare providers blinded?

        - Definitely no

        - The open-label nature of the trial means healthcare providers were not blinded.

    3c. Were data collectors blinded?

        - Probably no

© 2024 Lai H et al. *JAMA Network Open.*

- Blinding of data collectors is not described. The open-label design suggests they were likely not blinded.

3d. Were outcome assessors blinded?

- Probably no

- Blinding of outcome assessors is not described. The open-label design suggests they were likely not blinded.

3e. Were data analysts blinded?

- Probably no

- Blinding of data analysts is not described. The open-label design suggests they were likely not blinded.

4. Was loss to follow-up infrequent?

- Probably yes

- 4 patients out of 32 randomized (12.5%) did not complete the study. Loss to follow-up appears reasonably balanced between groups.

5. Are reports of the study free of selective outcome reporting?

- Probably yes

- The protocol is not available, but the pre-specified outcomes appear to be reported.

6. Was the study apparently free of other problems that could put it at a risk of bias?

- Probably yes

- No other apparent issues. The study appears reasonably well conducted overall given the open-label design.

© 2024 Lai H et al. *JAMA Network Open.*

ID: Griffin, 2013

First response:

Based on the information provided in the RCT paper by Griffin et al. (2013) and the guidelines you provided, here is my assessment for the Weight outcome:

Article ID: Griffin, 2013

Outcome Name: Weight

1. Was the allocation sequence adequately generated?

    - Probably yes

    - The paper states "Participants were randomized using computer-generated random numbers" which suggests adequate sequence generation.

2. Was the allocation adequately concealed?

    - Probably yes

    - The paper states "allocation was performed by an independent researcher not involved in the trial via telephone". Central allocation by an independent researcher suggests adequate concealment.

3. Blinding: Was knowledge of the allocated interventions adequately prevented?

3a. Were patients blinded?

    - Probably no

    - The paper states "Participants were blind to diet macronutrient composition" but they were informed about differences in meat type between diets, so blinding of patients was likely inadequate.

3b. Were healthcare providers blinded?

    - Probably no

    - No information provided, so blinding of providers was likely inadequate.

3c. Were data collectors blinded?

    - Probably no

    - No information provided, so blinding of data collectors was likely inadequate.

3d. Were outcome assessors blinded?

    - Probably no

© 2024 Lai H et al. *JAMA Network Open.*

- No information provided, so blinding of outcome assessors was likely inadequate.

3e. Were data analysts blinded?

- Probably no

- No information provided, so blinding of data analysts was likely inadequate.

4. Was loss to follow-up infrequent?

- Probably yes

- 49% attrition rate overall. HP group had 42% attrition and HC group had 57% attrition at 12 months. Attrition rates were high but balanced between groups.

5. Are reports of the study free of selective outcome reporting?

- Probably yes

- Pre-specified outcomes of weight, body composition, and biochemical measures seem to be adequately reported.

6. Was the study apparently free of other problems that could put it at a risk of bias?

- Probably yes

- No other apparent biases noted.

© 2024 Lai H et al. *JAMA Network Open.*

ID: ID: Griffin, 2013

Second response:

Here is my evaluation of the randomized controlled trial by Griffin et al. 2013 for the outcome of weight:

Article ID: Griffin, 2013

Outcome Name: Weight

1. Was the allocation sequence adequately generated?

- Probably yes

- The article states "A computer-generated random number sequence was used to allocate participants to HP or HC" which indicates adequate random sequence generation.

2. Was the allocation adequately concealed?

- Probably yes

- The article does not provide details on allocation concealment, but states that participants were blinded to the diet macronutrient composition, indicating probable adequate concealment.

3. Blinding: Was knowledge of the allocated interventions adequately prevented?

3a. Were patients blinded?

- Probably yes

- The article states "Participants were blind to diet macronutrient composition" indicating patients were probably adequately blinded.

3b. Were healthcare providers blinded?

- Probably no

- The article does not mention blinding of healthcare providers.

3c. Were data collectors blinded?

© 2024 Lai H et al. *JAMA Network Open.*

- Probably no

- The article does not mention blinding of data collectors.

3d. Were outcome assessors blinded?

- Probably no

- The article does not mention blinding of outcome assessors.

3e. Were data analysts blinded?

- Probably no

- The article does not mention blinding of data analysts.

4. Was loss to follow-up (missing outcome data) infrequent?

- Probably yes

- 49% of participants did not complete the study, however the article states "Attrition was not significantly different between diets" indicating the missing data was likely balanced between groups.

5. Are reports of the study free of selective outcome reporting?

- Probably yes

- The article does not mention availability of a protocol. However, it appears that expected weight outcomes were pre-specified and reported.

6. Was the study apparently free of other problems that could put it at a risk of bias?

- Probably yes

- The study appears to be free of other sources of bias based on the information provided.

© 2024 Lai H et al. *JAMA Network Open.*

ID: Hunninghake, 2000

First response:

Thank you for providing the full text article and detailed instructions. Here is my assessment of the risk of bias for the primary outcome of LDL cholesterol in this RCT:

Article ID: Hunninghake et al., 2000

Outcome Name: LDL cholesterol

1. Was the allocation sequence adequately generated?

- Probably yes

- The article states "Subjects were randomized using a random number generator", which suggests an adequate method was used. However, no further details are provided.

2. Was the allocation adequately concealed?

- Probably no

- The article does not mention any methods used for allocation concealment.

3. Blinding: Was knowledge of the allocated interventions adequately prevented?

3a. Were patients blinded?

- Definitely no

- The article states "This was an unblinded trial", indicating patients were not blinded.

3b. Were healthcare providers blinded?

- Definitely no

- The article states "This was an unblinded trial", indicating healthcare providers were not blinded.

3c. Were data collectors blinded?

- Definitely no

- The article states "This was an unblinded trial", indicating data collectors were not blinded.

© 2024 Lai H et al. *JAMA Network Open.*

3d. Were outcome assessors blinded?

- Definitely no

- The article states "This was an unblinded trial", indicating outcome assessors were not blinded.

3e. Were data analysts blinded?

- Probably no

- The article does not mention blinding of data analysts. As this was an unblinded trial, it is unlikely the data analysts were blinded.

4. Was loss to follow-up (missing outcome data) infrequent?

- Probably yes

- 42/202 (21%) dropped out during Phase I and 29/160 (18%) during Phase II. Though the dropout rates are moderately high, they are balanced between groups and unlikely to affect the outcomes.

5. Are reports of the study free of selective outcome reporting?

- Probably yes

- The study protocol is not available, but it appears all expected outcomes are reported.

6. Was the study apparently free of other problems that could put it at a risk of bias?

- Probably yes

- No other apparent biases identified.

© 2024 Lai H et al. *JAMA Network Open.*

ID: Hunninghake, 2000

Second response:

Thank you for providing the detailed guidelines and for explaining the role setting. I will follow your instructions carefully in evaluating the methodological quality of the randomized controlled trial from the article:

Article ID: Hunninghake, 2000

Outcome Name: LDL Cholesterol

1. **Was the allocation sequence adequately generated?**

 - Probably yes

 - The article states "Two hundred two individuals were randomly assigned to either the lean red meat (n = 95) or lean white meat (n = 107) treatment group during Phase I." However, it does not provide details on how the random sequence was generated. Based on the limited information provided, the allocation sequence was probably adequately generated.

2. **Was the allocation adequately concealed?**

 - Probably no

 - The article does not mention any methods used for allocation concealment. Therefore, there is no evidence that the allocation was adequately concealed.

3. **Blinding: Was knowledge of the allocated interventions adequately prevented?**

3a. Were patients blinded?

 - Definitely no

 - The article states that subjects were instructed on their diet assignment, indicating that patients were aware of the intervention.

3b. Were healthcare providers blinded?

© 2024 Lai H et al. *JAMA Network Open.*

- Definitely no
- The article states "Subjects in the lean red meat group were instructed to consume at least 80% of their total meat in the form of lean beef, veal or pork. Subjects randomized to the lean white meat group were similarly instructed, except that at least 80% of their meat consumption was to be lean white meat defined as poultry or fish." This indicates that healthcare providers were aware of diet assignments.

3c. Were data collectors blinded?
- Probably no
- The article does not mention blinding of data collectors. There is no evidence that data collectors were blinded.

3d. Were outcome assessors blinded?
- Probably no
- The article does not mention blinding of outcome assessors. There is no evidence that outcome assessors were blinded.

3e. Were data analysts blinded?
- Probably no
- The article does not mention blinding of data analysts. There is no evidence that data analysts were blinded.

4. **Was loss to follow-up (missing outcome data) infrequent?**
- Probably yes
- 145 subjects completed at least one blood draw in Phase II and were included in the analyses. 29 subjects dropped out prior to study completion. The follow-up rate was approximately 83%, which can be considered probably infrequent loss to follow-up.

5. **Are reports of the study free of selective outcome reporting?**
- Probably yes

© 2024 Lai H et al. *JAMA Network Open.*

- The study appears to report on the pre-specified outcomes of interest, including LDL cholesterol. There is no clear evidence of selective outcome reporting.

6. **Was the study apparently free of other problems that could put it at a risk of bias?**
   - Probably yes
   - The study appears to be free of other obvious sources of bias based on the information provided.

Outcome Name: HDL Cholesterol

1. **Was the allocation sequence adequately generated?**
   - Probably yes
   - The article states "Two hundred two individuals were randomly assigned to either the lean red meat (n = 95) or lean white meat (n = 107) treatment group during Phase I." However, it does not provide details on how the random sequence was generated. Based on the limited information provided, the allocation sequence was probably adequately generated.

2. **Was the allocation adequately concealed?**
   - Probably no
   - The article does not mention any methods used for allocation concealment. Therefore, there is no evidence that the allocation was adequately concealed.

3. **Blinding: Was knowledge of the allocated interventions adequately prevented?**

3a. Were patients blinded?
   - Definitely no
   - The article states that subjects were instructed on their diet assignment, indicating that patients were aware of the intervention.

3b. Were healthcare providers blinded?
   - Definitely no

© 2024 Lai H et al. *JAMA Network Open.*

- The article states "Subjects in the lean red meat group were instructed to consume at least 80% of their total meat in the form of lean beef, veal or pork. Subjects randomized to the lean white meat group were similarly instructed, except that at least 80% of their meat consumption was to be lean white meat defined as poultry or fish." This indicates that healthcare providers were aware of diet assignments.

3c. Were data collectors blinded?

- Probably no
- The article does not mention blinding of data collectors. There is no evidence that data collectors were blinded.

3d. Were outcome assessors blinded?

- Probably no
- The article does not mention blinding of outcome assessors. There is no evidence that outcome assessors were blinded.

3e. Were data analysts blinded?

- Probably no
- The article does not mention blinding of data analysts. There is no evidence that data analysts were blinded.

4. **Was loss to follow-up (missing outcome data) infrequent?**

- Probably yes
- 145 subjects completed at least one blood draw in Phase II and were included in the analyses. 29 subjects dropped out prior to study completion. The follow-up rate was approximately 83%, which can be considered probably infrequent loss to follow-up.

5. **Are reports of the study free of selective outcome reporting?**

- Probably yes

© 2024 Lai H et al. *JAMA Network Open.*

- The study appears to report on the pre-specified outcomes of interest, including HDL cholesterol. There is no clear evidence of selective outcome reporting.

6. **Was the study apparently free of other problems that could put it at a risk of bias?**

   - Probably yes

   - The study appears to be free of other obvious sources of bias based on the information provided.

Please let me know if I have followed the instructions correctly and evaluated the RCT appropriately according to the guidelines. I am happy to provide any clarification or make any corrections.

© 2024 Lai H et al. *JAMA Network Open.*

ID: Lanza, 2007

First response:

Thank you for providing the detailed guidelines and background information. I will evaluate the methodological quality of the randomized controlled trial reported in the article by Lanza et al. (2007) for the primary outcome of adenoma recurrence. Here is my assessment:

Article ID: Lanza, 2007

Outcome Name: Adenoma recurrence

1. **Was the allocation sequence adequately generated?**

   - Probably yes

   - The article states that "Randomization took place at T0" but does not provide details on how the allocation sequence was generated. However, since this was a multicenter randomized clinical trial, it is likely that an adequate randomization method was used.

2. **Was the allocation adequately concealed?**

   - Probably yes

   - The article does not mention allocation concealment, but since this was a multicenter randomized trial, it is likely that allocation was adequately concealed, such as through central allocation.

3. Blinding: Was knowledge of the allocated interventions adequately prevented?

3.a. Were patients blinded?

   - Probably no

   - The article does not mention blinding of patients. Since this was a dietary intervention trial, it is unlikely that patients were blinded.

3.b. Were healthcare providers blinded?

   - Probably no

   - The article does not mention blinding of healthcare providers. Since this was a dietary intervention trial, it is unlikely that providers were blinded.

© 2024 Lai H et al. *JAMA Network Open.*

3.c. Were data collectors blinded?

- Probably no
- The article does not mention blinding of data collectors.

3.d. Were outcome assessors blinded?

- Probably no
- The article does not mention blinding of outcome assessors.

3.e. Were data analysts blinded?

- Probably no
- The article does not mention blinding of data analysts.

4. **Was loss to follow-up (missing outcome data) infrequent?**

- Probably yes
- Of the 1905 participants who completed the initial trial, 1192 (62.6%) consented to participate in the continued follow-up study. Outcome data was obtained for 801 participants who underwent colonoscopy in the follow-up period. The reasons for missing outcome data are likely unrelated to the outcome. A multiple imputation analysis was done to account for potential selection bias due to missing data.

5. **Are reports of the study free of selective outcome reporting?**

- Probably yes
- The study protocol is not available, but it appears that the main outcomes related to adenoma recurrence are reported as would be expected in this type of trial.

6. **Was the study apparently free of other problems that could put it at a risk of bias?**

- Probably yes
- The study appears to be free of other sources of bias.

© 2024 Lai H et al. *JAMA Network Open.*

ID: Lanza, 2007

Second response:

Thank you for providing the detailed article and evaluation guidelines. Here is my assessment of the methodological quality for the primary outcome of adenoma recurrence in the Polyp Prevention Trial Continued Follow-up Study:

Article ID: Lanza et al., 2007

Outcome Name: Adenoma recurrence

1. Was the allocation sequence adequately generated?

- Definitely yes

- "Participants were randomized to either the intervention or control group with equal probability using a stratified permuted block algorithm"

2. Was the allocation adequately concealed?

- Probably yes

- The method of concealment is not specified, but randomization was performed centrally by the coordinating center, suggesting adequate concealment.

3. Blinding: Was knowledge of the allocated interventions adequately prevented?

3a. Were patients blinded?

- Definitely no

- Patients were not blinded since this was a dietary intervention trial.

3b. Were healthcare providers blinded?

- Definitely no

- Healthcare providers were not blinded since they had to deliver the dietary intervention.

3c. Were data collectors blinded?

- Probably yes

- Blinding of data collectors is not specified but may have been feasible.

3d. Were outcome assessors blinded?

- Probably yes

© 2024 Lai H et al. *JAMA Network Open.*

- Blinding of outcome assessors is not specified but may have been feasible given this was an objective outcome (adenoma recurrence)

3e. Were data analysts blinded?

- Probably yes
- Blinding of data analysts is not specified but may have been feasible.

4. Was loss to follow-up (missing outcome data) infrequent?

- Probably yes
- 62.6% of participants consented to participate in the continued follow-up study. Missing outcome data is partially addressed through multiple imputation.

5. Are reports of the study free of selective outcome reporting?

- Probably yes
- The protocol is not available but the published report seems to include the expected outcomes.

6. Was the study apparently free of other problems that could put it at risk of bias?

- Probably yes
- The study appears to be free of other sources of bias.

© 2024 Lai H et al. *JAMA Network Open.*

ID: Murphy, 2012

First response:

Here is my evaluation of the randomized controlled trial by Murphy et al. (2012) based on the guidelines provided:

Article ID: Murphy, 2012

Outcome Name: Percent body fat

1. Was the allocation sequence adequately generated?

    - Probably yes

    - The authors state that "Eligible volunteers were stratified according to gender, BMI and age and randomly allocated to one of two groups. Randomization was by minimization." Minimization is an acceptable method of random sequence generation.

2. Was the allocation adequately concealed?

    - Probably no

    - No details are provided on allocation concealment.

3. Blinding: Was knowledge of the allocated interventions adequately prevented?

    3a. Were patients blinded?

        - Probably no

        - No details are provided on blinding of patients.

    3b. Were healthcare providers blinded?

        - Probably no

        - No details are provided on blinding of healthcare providers.

    3c. Were data collectors blinded?

        - Probably no

        - No details are provided on blinding of data collectors.

    3d. Were outcome assessors blinded?

© 2024 Lai H et al. *JAMA Network Open.*

- Probably no

- No details are provided on blinding of outcome assessors.

3e. Were data analysts blinded?

- Probably no

- No details are provided on blinding of data analysts.

4. Was loss to follow-up (missing outcome data) infrequent?

- Probably yes

- 20 of the 164 participants who commenced the study withdrew or were lost to follow up. The authors state "There were no statistical differences in baseline characteristics of volunteers who withdrew or completed the study."

5. Are reports of the study free of selective outcome reporting?

- Probably yes

- The study protocol is not available, but the published report appears to include the expected outcomes, including the pre-specified primary outcome of percent body fat.

6. Was the study apparently free of other problems that could put it at a risk of bias?

- Probably yes

- The study appears to be free of other sources of bias.

Outcome Name: Fat mass

1. Was the allocation sequence adequately generated?

- Probably yes

- The authors state that "Eligible volunteers were stratified according to gender, BMI and age and randomly allocated to one of two groups. Randomization was by minimization." Minimization is an acceptable method of random sequence generation.

2. Was the allocation adequately concealed?

© 2024 Lai H et al. *JAMA Network Open.*

- Probably no

- No details are provided on allocation concealment.

3. Blinding: Was knowledge of the allocated interventions adequately prevented?

3a. Were patients blinded?

- Probably no

- No details are provided on blinding of patients.

3b. Were healthcare providers blinded?

- Probably no

- No details are provided on blinding of healthcare providers.

3c. Were data collectors blinded?

- Probably no

- No details are provided on blinding of data collectors.

3d. Were outcome assessors blinded?

- Probably no

- No details are provided on blinding of outcome assessors.

3e. Were data analysts blinded?

- Probably no

- No details are provided on blinding of data analysts.

4. Was loss to follow-up (missing outcome data) infrequent?

- Probably yes

- 20 of the 164 participants who commenced the study withdrew or were lost to follow up. The authors state "There were no statistical differences in baseline characteristics of volunteers who withdrew or completed the study."

5. Are reports of the study free of selective outcome reporting?

- Probably yes

- The study protocol is not available, but the published report appears to include the expected outcomes, including fat mass.

© 2024 Lai H et al. *JAMA Network Open.*

6. Was the study apparently free of other problems that could put it at a risk of bias?

- Probably yes
- The study appears to be free of other sources of bias.

© 2024 Lai H et al. *JAMA Network Open.*

ID: Murphy, 2012

Second response:

Based on the guidelines provided and the information reported in the article "Effects of Eating Fresh Lean Pork on Cardiometabolic Health Parameters" by Murphy et al. (2012), here are the evaluation results for the primary outcome of body weight:

Article ID: Murphy, 2012

Outcome Name: Body weight

1. Was the allocation sequence adequately generated?

- Response: Probably no

- Reason: No details are provided on how the randomization sequence was generated. According to the guidelines, if no details are available to support the judgment, select "Probably no".

2. Was the allocation adequately concealed?

- Response: Probably no

- Reason: No details are provided on allocation concealment. According to the guidelines, if no details are available to support the judgment, select "Probably no".

3. Blinding: Was knowledge of the allocated interventions adequately prevented?

3a. Were patients blinded?

- Response: Probably no

- Reason: No detail is available to support the assessment. According to the guidelines, if no detail is available to support the assessment, select “Probably no.”

3b. Were healthcare providers blinded?

- Response: Probably no

- Reason: No detail is available to support the assessment. According to the guidelines, if no detail is available to support the assessment, select “Probably no.”

3c. Were data collectors blinded?

© 2024 Lai H et al. *JAMA Network Open.*

- Response: Probably no

- Reason: No detail is available to support the assessment. According to the guidelines, if no detail is available to support the assessment, select “Probably no.”

3d. Were outcome assessors blinded?

- Response: Probably no

- Reason: No detail is available to support the assessment. According to the guidelines, if no detail is available to support the assessment, select “Probably no.”

3e. Were data analysts blinded?

- Response: Probably no

- Reason: No detail is available to support the assessment. According to the guidelines, if no detail is available to support the assessment, select “Probably no.”

4. Was loss to follow-up (missing outcome data) infrequent?

- Response: Probably no

- Reason: No detail is available to support the assessment. According to the guidelines, if no details are available to support the assessment, select “Probably no”.

5. Are reports of the study free of selective outcome reporting?

- Response: Probably yes

- Reason: The study protocol is not available but it is clear that the published reports include the pre-specified primary outcome of body weight. According to the guidelines, if the study protocol is not available but it is clear that the published reports include all expected outcomes, including those that were pre-specified, select “Probably yes.”

6. Was the study apparently free of other problems that could put it at a risk of bias?

- Response: Probably yes

- Reason: The study appears to be free of other sources of bias. According to the guidelines, if the study appears to be free of other sources of bias, select “Definitely yes.”

© 2024 Lai H et al. *JAMA Network Open.*

Please let me know if I have followed the guidelines correctly and provided the evaluation in the requested format. I am happy to revise my response if needed.

ID: Poddar, 2013

First response:

Here are the risk of bias assessments for the Poddar et al. 2013 study:

Article ID: Poddar, 2013

Outcome Name: Body weight

1. Was the allocation sequence adequately generated?

- Probably yes

- The study states "Prior to Visit 1, eligible participants were randomized into one of two treatment groups" but does not provide details on how the randomization sequence was generated.

2. Was the allocation adequately concealed?

- Probably no

- No details are provided on allocation concealment.

3. Blinding: Was knowledge of the allocated interventions adequately prevented?

3a. Were patients blinded? - Probably no

    - No details provided on blinding of patients

3b. Were healthcare providers blinded? - Probably no

    - No details provided on blinding of providers

3c. Were data collectors blinded? - Probably no

    - No details provided on blinding of data collectors

3d. Were outcome assessors blinded? - Probably no

    - No details provided on blinding of outcome assessors

3e. Were data analysts blinded? - Probably no

    - No details provided on blinding of data analysts

4. Was loss to follow-up infrequent?

© 2024 Lai H et al. *JAMA Network Open.*

- Probably no

- 65% dropout rate, no details provided on reasons for dropout or whether it was related to outcomes.

5. Are reports of the study free of selective outcome reporting?

- Probably yes

- The study protocol is not available but it appears that the expected outcomes were reported.

6. Was the study apparently free of other problems that could put it at risk of bias?

- Probably yes

- The study appears free of other sources of bias based on the information provided.

Outcome Name: Blood pressure

1. Was the allocation sequence adequately generated?

- Probably yes

- The study states "Prior to Visit 1, eligible participants were randomized into one of two treatment groups" but does not provide details on how the randomization sequence was generated.

2. Was the allocation adequately concealed?

- Probably no

- No details provided on allocation concealment.

3. Blinding: Was knowledge of the allocated interventions adequately prevented?

3a. Were patients blinded? - Probably no

    - No details provided on blinding of patients

3b. Were healthcare providers blinded? - Probably no

    - No details provided on blinding of providers

3c. Were data collectors blinded? - Probably no

    - No details provided on blinding of data collectors

3d. Were outcome assessors blinded? - Probably no

© 2024 Lai H et al. *JAMA Network Open.*

- No details provided on blinding of outcome assessors

3e. Were data analysts blinded? - Probably no

- No details provided on blinding of data analysts

4. Was loss to follow-up infrequent?

- Probably no

- 65% dropout rate, no details provided on reasons for dropout or whether it was related to outcomes.

5. Are reports of the study free of selective outcome reporting?

- Probably yes

- The study protocol is not available but it appears that the expected outcomes were reported.

6. Was the study apparently free of other problems that could put it at risk of bias?

- Probably yes

- The study appears free of other sources of bias based on the information provided.

Article ID: Poddar, 2013

Outcome Name: Blood lipid profile

1. Was the allocation sequence adequately generated?

- Probably yes

- The study states "Prior to Visit 1, eligible participants were randomized into one of two treatment groups" but does not provide details on how the randomization sequence was generated.

2. Was the allocation adequately concealed?

- Probably no

- No details are provided on allocation concealment.

3. Blinding: Was knowledge of the allocated interventions adequately prevented?

© 2024 Lai H et al. *JAMA Network Open.*

3a. Were patients blinded? - Probably no

- No details provided on blinding of patients

3b. Were healthcare providers blinded? - Probably no

- No details provided on blinding of providers

3c. Were data collectors blinded? - Probably no

- No details provided on blinding of data collectors

3d. Were outcome assessors blinded? - Probably no

- No details provided on blinding of outcome assessors

3e. Were data analysts blinded? - Probably no

- No details provided on blinding of data analysts

4. Was loss to follow-up infrequent?

- Probably no

- 65% dropout rate, no details provided on reasons for dropout or whether it was related to outcomes.

5. Are reports of the study free of selective outcome reporting?

- Probably yes

- The study protocol is not available but it appears that the expected outcomes were reported.

6. Was the study apparently free of other problems that could put it at risk of bias?

- Probably yes

- The study appears free of other sources of bias based on the information provided.

© 2024 Lai H et al. *JAMA Network Open.*

ID: Poddar, 2013

Second response:

Here are the risk of bias assessments for the RCT reported in the provided article:

Article ID: Poddar et al., 2013

Outcome Name: Body weight

1. Was the allocation sequence adequately generated?

- Probably yes

- The article states "Prior to Visit 1, eligible participants were randomized into one of two treatment groups", but does not provide details on how the randomization sequence was generated.

2. Was allocation adequately concealed?

- Probably no

- No details are provided on allocation concealment.

3. Blinding: Was knowledge of the allocated interventions adequately prevented?

3a. Were patients blinded?

- Probably no

- No details are provided on blinding of patients.

3b. Were healthcare providers blinded?

- Probably no

- No details are provided on blinding of healthcare providers.

3c. Were data collectors blinded?

- Probably no

- No details are provided on blinding of data collectors.

3d. Were outcome assessors blinded?

- Probably no

- No details are provided on blinding of outcome assessors.

© 2024 Lai H et al. *JAMA Network Open.*

3e. Were data analysts blinded?

- Probably no
- No details are provided on blinding of data analysts.

4. Was loss to follow-up infrequent?

- Probably no
- The non-completion rate was 65% after 12 months. No details are provided on reasons for missing data.

5. Are reports of the study free of selective outcome reporting?

- Probably yes
- The study protocol is not available, but it appears that the expected outcomes are reported.

6. Was the study apparently free of other problems that could put it at risk of bias?

- Probably yes
- The study appears to be free of other sources of bias.

Outcome Name: BMI

1. Was the allocation sequence adequately generated?

- Probably yes
- The article states "Prior to Visit 1, eligible participants were randomized into one of two treatment groups", but does not provide details on how the randomization sequence was generated.

2. Was allocation adequately concealed?

- Probably no
- No details are provided on allocation concealment.

3. Blinding: Was knowledge of the allocated interventions adequately prevented?

3a. Were patients blinded?

© 2024 Lai H et al. *JAMA Network Open.*

- Probably no

- No details are provided on blinding of patients.

3b. Were healthcare providers blinded?

- Probably no

- No details are provided on blinding of healthcare providers.

3c. Were data collectors blinded?

- Probably no

- No details are provided on blinding of data collectors.

3d. Were outcome assessors blinded?

- Probably no

- No details are provided on blinding of outcome assessors.

3e. Were data analysts blinded?

- Probably no

- No details are provided on blinding of data analysts.

4. Was loss to follow-up infrequent?

- Probably no

- The non-completion rate was 65% after 12 months. No details are provided on reasons for missing data.

5. Are reports of the study free of selective outcome reporting?

- Probably yes

- The study protocol is not available, but it appears that the expected outcomes are reported.

6. Was the study apparently free of other problems that could put it at risk of bias?

- Probably yes

- The study appears to be free of other sources of bias.

Outcome Name: Waist circumference

© 2024 Lai H et al. *JAMA Network Open.*

1. Was the allocation sequence adequately generated?

- Probably yes
- The article states "Prior to Visit 1, eligible participants were randomized into one of two treatment groups", but does not provide details on how the randomization sequence was generated.

2. Was allocation adequately concealed?

- Probably no
- No details are provided on allocation concealment.

3. Blinding: Was knowledge of the allocated interventions adequately prevented?

3a. Were patients blinded?

- Probably no
- No details are provided on blinding of patients.

3b. Were healthcare providers blinded?

- Probably no
- No details are provided on blinding of healthcare providers.

3c. Were data collectors blinded?

- Probably no
- No details are provided on blinding of data collectors.

3d. Were outcome assessors blinded?

- Probably no
- No details are provided on blinding of outcome assessors.

3e. Were data analysts blinded?

- Probably no
- No details are provided on blinding of data analysts.

4. Was loss to follow-up infrequent?

- Probably no
- The non-completion rate was 65% after 12 months. No details are provided on reasons for missing data.

© 2024 Lai H et al. *JAMA Network Open.*

5. Are reports of the study free of selective outcome reporting?

- Probably yes
- The study protocol is not available, but it appears that the expected outcomes are reported.

6. Was the study apparently free of other problems that could put it at risk of bias?

- Probably yes
- The study appears to be free of other sources of bias.

Outcome Name: Percent total body fat

1. Was the allocation sequence adequately generated?

- Probably yes
- The article states "Prior to Visit 1, eligible participants were randomized into one of two treatment groups", but does not provide details on how the randomization sequence was generated.

2. Was allocation adequately concealed?

- Probably no
- No details are provided on allocation concealment.

3. Blinding: Was knowledge of the allocated interventions adequately prevented?

3a. Were patients blinded?

- Probably no
- No details are provided on blinding of patients.

3b. Were healthcare providers blinded?

- Probably no
- No details are provided on blinding of healthcare providers.

3c. Were data collectors blinded?

- Probably no
- No details are provided on blinding of data collectors.

© 2024 Lai H et al. *JAMA Network Open.*

3d. Were outcome assessors blinded?

- Probably no
- No details are provided on blinding of outcome assessors.

3e. Were data analysts blinded?

- Probably no
- No details are provided on blinding of data analysts.

4. Was loss to follow-up infrequent?

- Probably no
- The non-completion rate was 65% after 12 months. No details are provided on reasons for missing data.

5. Are reports of the study free of selective outcome reporting?

- Probably yes
- The study protocol is not available, but it appears that the expected outcomes are reported.

6. Was the study apparently free of other problems that could put it at risk of bias?

- Probably yes
- The study appears to be free of other sources of bias.

© 2024 Lai H et al. *JAMA Network Open.*

ID: Turner-McGrievy, 2015

First response:

Thank you for providing the detailed guidelines and examples for evaluating randomized controlled trials. I will follow the instructions closely to assess the risk of bias for the RCT provided in the Turner-McGrievy et al. (2014) article. Here are the evaluations:

Article ID: Turner-McGrievy et al., 2014

Outcome Name: Weight loss

1. Was the allocation sequence adequately generated?

- Probably yes. The article states "Participants were then randomized to one of the five diets using a computerized random-number generator."

- A computerized random number generator indicates the allocation sequence was likely adequately generated.

2. Was the allocation adequately concealed?

- Probably no. The article does not provide details on allocation concealment.

3. Blinding: Was knowledge of the allocated interventions adequately prevented?

3a. Were patients blinded?

- Probably no. The article does not provide details on blinding of patients.

3b. Were healthcare providers blinded?

- Probably no. The article does not provide details on blinding of healthcare providers.

3c. Were data collectors blinded?

- Probably no. The article does not provide details on blinding of data collectors.

3d. Were outcome assessors blinded?

© 2024 Lai H et al. *JAMA Network Open.*

- Probably no. The article does not provide details on blinding of outcome assessors.

3e. Were data analysts blinded?

- Probably no. The article does not provide details on blinding of data analysts.

4. Was loss to follow-up (missing outcome data) infrequent?

- Probably yes. The article states "At six months, 50 (79%) completed the study (i.e., provided a body-weight measurement at 6 months)". The follow-up rate was 79%, which is close to 80%.

5. Are reports of the study free of selective outcome reporting?

- Probably yes. The article does not have a published protocol, but it appears all expected outcomes are reported.

6. Was the study apparently free of other problems that could put it at a risk of bias?

- Probably yes. The study appears free of other apparent sources of bias based on the information provided.

Article ID: Turner-McGrievy et al., 2014

Outcome Name: Dietary intake

1. Was the allocation sequence adequately generated?

- Probably yes. The article states "Participants were then randomized to one of the five diets using a computerized random-number generator."

- A computerized random number generator indicates the allocation sequence was likely adequately generated.

2. Was the allocation adequately concealed?

- Probably no. The article does not provide details on allocation concealment.

© 2024 Lai H et al. *JAMA Network Open.*

3. Blinding: Was knowledge of the allocated interventions adequately prevented?

3a. Were patients blinded?

- Probably no. The article does not provide details on blinding of patients.

3b. Were healthcare providers blinded?

- Probably no. The article does not provide details on blinding of healthcare providers.

3c. Were data collectors blinded?

- Probably no. The article does not provide details on blinding of data collectors.

3d. Were outcome assessors blinded?

- Probably no. The article does not provide details on blinding of outcome assessors.

3e. Were data analysts blinded?

- Probably no. The article does not provide details on blinding of data analysts.

4. Was loss to follow-up (missing outcome data) infrequent?

- Probably yes. Dietary intake data was available for 78-89% of participants at each timepoint.

5. Are reports of the study free of selective outcome reporting?

- Probably yes. The article does not have a published protocol, but it appears all expected outcomes are reported.

6. Was the study apparently free of other problems that could put it at a risk of bias?

- Probably yes. The study appears free of other apparent sources of bias based on the information provided.

Please let me know if you would like me to clarify or expand on any part of my evaluation. I aimed to closely follow the instructions provided.

© 2024 Lai H et al. *JAMA Network Open.*

ID: Turner-McGrievy, 2015

Second response:

Thank you for providing the detailed guidelines and background information. Here is my evaluation of the randomized controlled trial by Turner-McGrievy et al. (2014):

Article ID: Turner-McGrievy et al., 2014

Outcome Name: Percent weight loss

1. Was the allocation sequence adequately generated?

- Definitely yes

- "Participants were then randomized to one of the five diets using a computerized random-number generator"

2. Was the allocation adequately concealed?

- Probably no

- The method of concealment is not described.

3. Blinding: Was knowledge of the allocated interventions adequately prevented?

3a. Were patients blinded?

- Definitely no

- The nature of the dietary interventions makes blinding of patients impossible.

3b. Were healthcare providers blinded?

- Definitely no

- The nature of the dietary interventions makes blinding of healthcare providers impossible.

3c. Were data collectors blinded?

- Probably no

- Blinding of data collectors is not described.

© 2024 Lai H et al. *JAMA Network Open.*

3d. Were outcome assessors blinded?

- Probably no

- Blinding of outcome assessors is not described.

3e. Were data analysts blinded?

- Probably no

- Blinding of data analysts is not described.

4. Was loss to follow-up (missing outcome data) infrequent?

- Probably yes

- 79% of participants completed the study. The dropout rate was 21% at 6 months.

5. Are reports of the study free of selective outcome reporting?

- Definitely yes

- All outcomes specified in the methods section are reported in the results.

6. Was the study apparently free of other problems that could put it at a risk of bias?

- Definitely yes

- The study appears to be free of other sources of bias.

© 2024 Lai H et al. *JAMA Network Open.*

ID: Yaskolka, 2019

First response:

Here are the risk of bias assessments for the randomized controlled trial by Yaskolka Meir et al. (2019):

Article ID: Yaskolka Meir, 2019

Outcome Name: Changes in serum iron

1. Was the allocation sequence adequately generated?

- Response: Probably yes

- Reason: "After baseline measurements, the intervention started in 1 phase, after participants' random assignment to 1 of the 3 following groups:" The method of randomization is not described, but it appears there was random assignment to groups.

2. Was the allocation adequately concealed?

- Response: Probably no

- Reason: No details are provided on allocation concealment.

3a. Were patients blinded?

- Response: Probably no

- Reason: No details are provided on blinding of patients.

3b. Were healthcare providers blinded?

- Response: Probably no

- Reason: No details are provided on blinding of healthcare providers.

3c. Were data collectors blinded?

- Response: Probably no

- Reason: No details are provided on blinding of data collectors.

© 2024 Lai H et al. *JAMA Network Open.*

3d. Were outcome assessors blinded?

- Response: Probably no

- Reason: No details are provided on blinding of outcome assessors.

3e. Were data analysts blinded?

- Response: Probably no

- Reason: No details are provided on blinding of data analysts.

4. Was loss to follow-up infrequent?

- Response: Probably yes

- Reason: For 6-mo serum iron changes, there were 96 participants in each group, close to the original 98 per group, suggesting low dropout.

5. Are reports of the study free of selective outcome reporting?

- Response: Probably yes

- Reason: The pre-specified outcomes appear to be reported.

6. Was the study apparently free of other problems that could put it at a risk of bias?

- Response: Probably yes

- Reason: The study appears to be free of other sources of bias.

Outcome Name: Changes in blood hemoglobin

1. Was the allocation sequence adequately generated?

- Response: Probably yes

- Reason: Same as for previous outcome.

2. Was the allocation adequately concealed?

- Response: Probably no

© 2024 Lai H et al. *JAMA Network Open.*

- Reason: Same as for previous outcome.

3a. Were patients blinded?

- Response: Probably no
- Reason: Same as for previous outcome.

3b. Were healthcare providers blinded?

- Response: Probably no
- Reason: Same as for previous outcome.

3c. Were data collectors blinded?

- Response: Probably no
- Reason: Same as for previous outcome.

3d. Were outcome assessors blinded?

- Response: Probably no
- Reason: Same as for previous outcome.

3e. Were data analysts blinded?

- Response: Probably no
- Reason: Same as for previous outcome.

4. Was loss to follow-up infrequent?

- Response: Probably yes
- Reason: For 6-mo blood hemoglobin changes, there were 87 in the PA group, 84 in the PA + MED group, and 88 in the PA + green-MED group, close to the original 98 per group, suggesting low dropout.

5. Are reports of the study free of selective outcome reporting?

- Response: Probably yes

© 2024 Lai H et al. *JAMA Network Open.*

- Reason: Same as for previous outcome.

6. Was the study apparently free of other problems that could put it at a risk of bias?

  - Response: Probably yes

  - Reason: Same as for previous outcome.

© 2024 Lai H et al. *JAMA Network Open.*

ID: Yaskolka, 2019

Second response:

Here are the risk of bias assessments for the Yaskolka Meir et al. 2019 study:

Article ID: Yaskolka Meir et al., 2019

Outcome Name: Changes in iron homeostasis parameters

1. Was the allocation sequence adequately generated?

- Definitely yes

- "Participants were randomly assigned to 1 of the 3 following groups: PA, PA + MED diet, or PA + green-MED diet (a flowchart of the DIRECT-PLUS trial is provided in Supplemental Figure 1)."

2. Was the allocation adequately concealed?

- Probably no

- No details provided on allocation concealment.

3. Blinding: Was knowledge of the allocated interventions adequately prevented?

3a. Were patients blinded?

- Probably no

- No details provided on patient blinding.

3b. Were healthcare providers blinded?

- Probably no

- No details provided on healthcare provider blinding.

3c. Were data collectors blinded?

- Probably no

- No details provided on data collector blinding.

3d. Were outcome assessors blinded?

- Probably no

- No details provided on outcome assessor blinding.

© 2024 Lai H et al. *JAMA Network Open.*

3e. Were data analysts blinded?

- Probably no

- No details provided on data analyst blinding.

4. Was loss to follow-up (missing outcome data) infrequent?

- Probably yes

- For 6-mo serum iron, folic acid, transferrin saturation, transferrin, and ferritin changes: n=96 for each group; for 6-mo blood hemoglobin changes: PA: n=87; PA + MED: n=84; PA + green-MED: n=88. Dropout rates appear <10% at 6 months.

5. Are reports of the study free of selective outcome reporting?

- Probably yes

- The study protocol is not available, but it appears that the published report includes the expected outcomes related to iron homeostasis parameters.

6. Was the study apparently free of other problems that could put it at a risk of bias?

- Probably yes

- The study appears to be free of other sources of bias.

Article ID: Yaskolka Meir et al., 2019

Outcome Name: Changes in hemoglobin concentrations

1. Was the allocation sequence adequately generated?

- Definitely yes

- "Participants were randomly assigned to 1 of the 3 following groups: PA, PA + MED diet, or PA + green-MED diet (a flowchart of the DIRECT-PLUS trial is provided in Supplemental Figure 1)."

2. Was the allocation adequately concealed?

- Probably no

© 2024 Lai H et al. *JAMA Network Open.*

- No details provided on allocation concealment.

3. Blinding: Was knowledge of the allocated interventions adequately prevented?

3a. Were patients blinded?

- Probably no
- No details provided on patient blinding.

3b. Were healthcare providers blinded?

- Probably no
- No details provided on healthcare provider blinding.

3c. Were data collectors blinded?

- Probably no
- No details provided on data collector blinding.

3d. Were outcome assessors blinded?

- Probably no
- No details provided on outcome assessor blinding.

3e. Were data analysts blinded?

- Probably no
- No details provided on data analyst blinding.

4. Was loss to follow-up (missing outcome data) infrequent?

- Probably yes
- For 6-mo blood hemoglobin changes: PA: n=87; PA + MED: n=84; PA + green-MED: n=88. Dropout rates appear <10% at 6 months.

5. Are reports of the study free of selective outcome reporting?

- Probably yes
- The study protocol is not available, but it appears that the published report includes the expected outcome related to hemoglobin.

6. Was the study apparently free of other problems that could put it at a risk of bias?

© 2024 Lai H et al. *JAMA Network Open.*

- Probably yes

- The study appears to be free of other sources of bias.

© 2024 Lai H et al. *JAMA Network Open.*

ID: Carlson, 2019

First response:

Based on your instructions, here is my assessment of the RCT by Carlson et al. (2019):

Article ID: Carlson et al., 2019

Outcome Name: Hemoglobin A1c

1. Was the allocation sequence adequately generated?

   - Probably no

   - The study states "Subjects were randomized to one of three study arms," but does not provide details on how the randomization sequence was generated.

2. Was the allocation adequately concealed?

   - Probably no

   - The study does not provide details on allocation concealment. Since the exact randomization method is unspecified, allocation concealment cannot be definitely assessed.

3. Blinding: Was knowledge of the allocated interventions adequately prevented?

3.a. Were patients blinded?

   - Definitely no

   - This was an open-label study, so patients were aware of the interventions.

3.b. Were healthcare providers blinded?

   - Definitely no

   - This was an open-label study, so providers were aware of the interventions.

3.c. Were data collectors blinded?

   - Probably no

   - The study does not state whether data collectors were blinded.

3.d. Were outcome assessors blinded?

   - Probably no

© 2024 Lai H et al. *JAMA Network Open.*

- The study does not state whether outcome assessors were blinded.

3.e. Were data analysts blinded?

- Probably no

- The study does not state whether data analysts were blinded.

4. Was loss to follow-up (missing outcome data) infrequent?

- Probably yes

- 6 participants out of 60 randomized dropped out. For participants with insufficient CGM data, previous CGM metrics were carried forward in the analysis.

5. Are reports of the study free of selective outcome reporting?

- Probably yes

- The pre-specified outcomes generally appear to be reported, but without access to the study protocol, selective reporting cannot be ruled out.

6. Was the study apparently free of other problems that could put it at a risk of bias?

- Probably yes

- No other apparent issues.

Since there is limited information provided on some aspects like randomization, blinding and selective outcome reporting, I have avoided making definitive judgments, in line with your instructions. Please let me know if I should modify my assessment in any way.

© 2024 Lai H et al. *JAMA Network Open.*

ID: Carlson, 2019

Second response:

Here are the evaluation results for the primary outcome reported in the Carlson et al. (2019) RCT:

Article ID: Carlson et al., 2019

Outcome Name: Hemoglobin A1c (HbA1c)

1. Item name: Was the allocation sequence adequately generated?

   - Response: Probably yes

   - Reason: The study states "After the metformin run-in and a 14-day CGM baseline, participants were randomized to one of three study arms (while continuing metformin): Iglar, GLP-1 given twice daily, or a combination of Iglar + GLP-1." The exact method of sequence generation is not described, but randomization is mentioned.

2. Item name: Was the allocation adequately concealed?

   - Response: Probably yes

   - Reason: The study does not provide details on allocation concealment methods. However, since centralized randomization methods are common in RCTs, it is probable that adequate allocation concealment was done.

3. Item name: Blinding - Were patients blinded?

   - Response: Definitely no

   - Reason: The study states "Open-label, randomized, parallel-arm study". Patients were aware of the treatment they received.

3b. Item name: Were healthcare providers blinded?

   - Response: Definitely no

   - Reason: The study states "Open-label, randomized, parallel-arm study". Healthcare providers were aware of the treatment patients received.

© 2024 Lai H et al. *JAMA Network Open.*

3c. Item name: Were data collectors blinded?

- Response: Definitely no

- Reason: The study states "Open-label, randomized, parallel-arm study". Data collectors were aware of the treatment patients received.

3d. Item name: Were outcome assessors blinded?

- Response: Definitely no

- Reason: The study states "Open-label, randomized, parallel-arm study". Outcome assessors were aware of the treatment patients received.

3e. Item name: Were data analysts blinded?

- Response: Definitely no

- Reason: The study states "Open-label, randomized, parallel-arm study". Data analysts were aware of the treatment patients received.

4. Item name: Was loss to follow-up (missing outcome data) infrequent?

- Response: Probably yes

- Reason: The study states that 3 participants did not have sufficient follow-up CGM data, so CGM metrics from the previous period were carried forward. Overall attrition rate was not high (8 out of 60 participants). Missing data was balanced across groups.

5. Item name: Are reports of the study free of selective outcome reporting?

- Response: Probably yes

- Reason: The study protocol is not available, but the pre-specified primary outcome (HbA1c) and other expected outcomes are reported. There is no indication of selective reporting.

6. Item name: Was the study apparently free of other problems that could put it at a risk of bias?

- Response: Probably yes

- Reason: The study appears to be free of other sources of bias.

© 2024 Lai H et al. *JAMA Network Open.*

In summary, this open-label randomized trial has a high risk of performance bias due to lack of blinding. However, there are no other apparent concerns that would put the study at a high risk of bias for the HbA1c outcome.

© 2024 Lai H et al. *JAMA Network Open.*

ID: Cherney, 2021

First response:

Here is my evaluation of the methodological quality for the primary outcome HbA1c reduction in the RCT by Cherney et al., 2021:

Article ID: Cherney, 2021

Outcome Name: HbA1c reduction

1. Was the allocation sequence adequately generated?

- Definitely yes

- "Patients were randomly assigned 1:1:1 to treatment with placebo, sotagliflozin 200 mg, or sotagliflozin 400 mg" indicates adequate random sequence generation.

2. Was the allocation adequately concealed?

- Probably yes

- The method of allocation concealment is not described, but the study is described as "double-blind" which implies attempts were likely made to conceal allocation.

3. Blinding: Was knowledge of the allocated interventions adequately prevented?

3a. Were patients blinded?

- Definitely yes

- The study is described as "double-blind".

3b. Were healthcare providers blinded?

- Definitely yes

- The study is described as "double-blind".

3c. Were data collectors blinded?

- Definitely yes

© 2024 Lai H et al. *JAMA Network Open.*

- "Laboratory values...were determined by a central laboratory and masked to study sites and patients"

3d. Were outcome assessors blinded?

- Definitely yes
- "double-blind" and "All observed data were used in all efficacy analyses, including data gathered after study drug discontinuation or introduction of rescue therapy." indicates outcome assessors were blinded.

3e. Were data analysts blinded?

- Definitely yes
- "double-blind" indicates data analysts were likely blinded.

4. Was loss to follow-up (missing outcome data) infrequent?

- Definitely yes
- "All observed data were used in all efficacy analyses, including data gathered after study drug discontinuation or introduction of rescue therapy." Indicates full ITT analysis with no missing outcome data.

5. Are reports of the study free of selective outcome reporting?

- Definitely yes
- The pre-specified primary outcome of HbA1c reduction at 26 weeks is reported as planned.

6. Was the study apparently free of other problems that could put it at a risk of bias?

- Definitely yes
- The study appears to be free of other sources of bias.

© 2024 Lai H et al. *JAMA Network Open.*

ID: Cherney, 2021

Second response:

Here is my evaluation of the methodological quality for the primary outcome in the given RCT:

Article ID: Cherney et al, 2021

Outcome Name: Change in HbA1c at Week 26 (primary outcome)

1. Was the allocation sequence adequately generated?

- Definitely yes

- The article states "Patients were randomly assigned in a 1:1:1 ratio to placebo, sotagliflozin 200 mg, or sotagliflozin 400 mg once daily by use of an interactive web-response system that implemented a computer-generated random sequence using randomly permuted blocks." This indicates adequate random sequence generation using computer-generated random numbers.

2. Was the allocation adequately concealed?

- Definitely yes

- The article states "Randomization was stratified by screening HbA1c (≤8.5% vs >8.5%) and mean SBP (<130 mm Hg vs ≥130 mm Hg). Laboratory values, including FPG, HbA1c, and UGE were determined by a central laboratory and masked to study sites and patients from randomization until study end." This indicates central allocation and adequate concealment of allocation.

3. Blinding: Was knowledge of the allocated interventions adequately prevented?

3a. Were patients blinded?

- Definitely yes

- The article states "This phase 3, multicenter, randomized, double-blind, placebo-controlled study...". This indicates patients were blinded.

3b. Were healthcare providers blinded?

- Definitely yes

© 2024 Lai H et al. *JAMA Network Open.*

- The article states "This phase 3, multicenter, randomized, double-blind, placebo-controlled study...". This indicates healthcare providers were blinded.

3c. Were data collectors blinded?

- Definitely yes

- The article states "Laboratory values, including FPG, HbA1c, and UGE were determined by a central laboratory and masked to study sites and patients from randomization until study end." This indicates blinding of data collectors.

3d. Were outcome assessors blinded?

- Definitely yes

- As the trial is described as double-blind and the central laboratory was blinded, the outcome assessors were also likely blinded.

3e. Were data analysts blinded?

- Definitely yes

- The article states "The primary multiple imputation algorithm applied to each endpoint was a mixture of the retrieved dropout and washout methods (see Supplementary Appendix). Each of the complete datasets following the multiple imputation step were analyzed using the ANCOVA model with treatment groups (sotagliflozin 400 mg, sotagliflozin 200 mg, placebo), randomization strata of HbA1c (≤8.5%, >8.5%) and SBP (<130 mmHg, ≥130 mmHg), and country as fixed effects, and the baseline endpoint value as a covariate." This indicates the data analysts were blinded during analysis.

4. Was loss to follow-up infrequent?

- Probably yes

- 93 patients were randomized to placebo and 75 completed 26 weeks. 94 patients were randomized to sotagliflozin 200mg and 78 completed 26 weeks. 92 patients were randomized to sotagliflozin 400mg and 77 completed 26 weeks. This indicates a follow-up rate of approximately 80-85% at 26 weeks. The reasons for discontinuation appear balanced across groups. Though the follow-up rate is

© 2024 Lai H et al. *JAMA Network Open.*

slightly below 90%, it is still reasonably high, so loss to follow-up can probably be considered infrequent.

5. Are reports of the study free of selective outcome reporting?

- Definitely yes
- The protocol does not appear to be available, but the pre-specified primary and secondary outcomes are reported as expected in the methods and results sections. There is no evidence of selective reporting.

6. Was the study apparently free of other problems that could put it at a risk of bias?

- Definitely yes
- The study appears to be free of other sources of bias.

© 2024 Lai H et al. *JAMA Network Open.*

ID: del Prato, 2014

First response:

Thank you for providing the detailed evaluation guidelines. I will follow them closely to assess the risk of bias for the randomized controlled trial by Del Prato et al. 2014. Here are the evaluation results:

Article ID: Del Prato, 2014

Outcome Name: Change in HbA1c from baseline to week 104

1. Was the allocation sequence adequately generated?

- Probably no

- The article states "patients were randomized in a 1:1:1 ratio" but does not provide details on how the randomization sequence was generated.

2. Was the allocation adequately concealed?

- Probably no

- The article does not provide details on allocation concealment.

3. Blinding: Was knowledge of the allocated interventions adequately prevented?

3a. Were patients blinded? - Probably yes

- The study is described as "double-blind" but details of blinding methods are not provided.

3b. Were healthcare providers blinded? - Probably yes

- Same reason as above.

3c. Were data collectors blinded? - Probably yes

- Same reason as above.

3d. Were outcome assessors blinded? - Probably yes

- Same reason as above.

3e. Were data analysts blinded? - Probably yes

- Same reason as above.

4. Was loss to follow-up (missing outcome data) infrequent?

© 2024 Lai H et al. *JAMA Network Open.*

- Probably yes

- 76-80% of patients completed the study or were rescued for hyperglycemia. The dropout rate at 104 weeks was 20-24%.

5. Are reports of the study free of selective outcome reporting?

- Probably yes

- The study protocol is not available but it appears that results for the pre-specified outcomes are fully reported.

6. Was the study apparently free of other problems that could put it at a risk of bias?

- Probably yes

- The study appears to be free of other sources of bias based on the information provided.

Outcome Name: Change in body weight from baseline to week 104

[Same responses and reasons for items 1-6]

Let me know if you would like me to evaluate any other outcomes from this study. As requested, when details were not available to support an assessment, I selected "Probably no". Please confirm if my evaluation followed the guidelines appropriately. I'm happy to clarify or revise my assessment as needed.

© 2024 Lai H et al. *JAMA Network Open.*

ID: del Prato, 2014

Second response:

Thank you for providing the detailed trial information and evaluation guidelines. Here is my assessment of the methodological quality for the primary outcome of change in HbA1c from baseline to week 104:

Article ID: Del Prato et al., 2014

Outcome Name: Change in HbA1c from baseline to week 104

1. Was the allocation sequence adequately generated?

- Definitely yes
- The article states "This double-blind, randomized, active-controlled, three-arm, parallel-group study was conducted" and "All patients were randomized in a 1:1:1 ratio to receive 104 weeks of double-blind treatment with either alogliptin 12.5 mg once daily, alogliptin 25 mg once daily or glipizide 5 mg once daily." This indicates that an adequate randomization method was used.

2. Was the allocation adequately concealed?

- Probably yes
- The study is described as double-blind, which implies that allocation was adequately concealed, however no specific details are provided on the concealment method.

3. Blinding: Was knowledge of the allocated interventions adequately prevented?

3a. Were patients blinded?

- Probably yes
- The study is described as double-blind, implying patients were blinded, however no specific details are provided.

3b. Were healthcare providers blinded?

- Probably yes

© 2024 Lai H et al. *JAMA Network Open.*

- The study is described as double-blind, implying providers were blinded, however no specific details are provided.

3c. Were data collectors blinded?

- Probably yes
- The study is described as double-blind, implying data collectors were blinded, however no specific details are provided.

3d. Were outcome assessors blinded?

- Probably yes
- The study is described as double-blind, implying outcome assessors were blinded, however no specific details are provided.

3e. Were data analysts blinded?

- Probably yes
- The study is described as double-blind, implying data analysts were blinded, however no specific details are provided.

4. Was loss to follow-up (missing outcome data) infrequent?

- Probably yes
- 76-80% of patients completed the study or were rescued for hyperglycemia across the three groups. Reasons for discontinuation appear balanced across groups. The dropout rate is less than 20% in all groups.

5. Are reports of the study free of selective outcome reporting?

- Probably yes
- The study protocol is not available, but the pre-specified primary outcome of change in HbA1c from baseline to week 104 seems to be fully reported.

6. Was the study apparently free of other problems that could put it at a risk of bias?

- Probably yes
- No other apparent biases noted based on the information provided. The study appears to be a well-designed RCT.

© 2024 Lai H et al. *JAMA Network Open.*

Please let me know if you would like me to evaluate any other outcomes from this trial. I am happy to provide additional assessments as needed.

© 2024 Lai H et al. *JAMA Network Open.*

ID: Frias, 2018

First response:

Thank you for specifying the outcome. Here is the risk of bias assessment for HbA1c in the Frias et al. 2018 study:

Article ID: Frias, 2018

Outcome Name: HbA1c

1. Was the allocation sequence adequately generated?

- Probably no

- The article states "participants...were randomly allocated", but does not provide details on how the randomization sequence was generated.

2. Was the allocation adequately concealed?

- Probably no

- The article does not provide details on allocation concealment.

3. Blinding: Was knowledge of the allocated interventions adequately prevented?

3a. Were patients blinded? Probably yes

3b. Were healthcare providers blinded? Probably yes

3c. Were data collectors blinded? Probably yes

3d. Were outcome assessors blinded? Probably yes

3e. Were data analysts blinded? Probably yes

- The article states this was a "double-blind" study, suggesting key personnel and participants were blinded, but details not provided.

4. Was loss to follow-up (missing outcome data) infrequent?

- Probably yes

- 258/316 (82%) participants completed 26 weeks of treatment. Loss to follow-up appears balanced across groups.

© 2024 Lai H et al. *JAMA Network Open.*

5. Are reports of the study free of selective outcome reporting?

- Probably yes

- The study protocol is not available, but it appears all expected outcomes are reported.

6. Was the study apparently free of other problems that could put it at a risk of bias?

- Probably yes

- The study appears free of other sources of bias.

© 2024 Lai H et al. *JAMA Network Open.*

ID: Frias, 2018

Second response:

Here is my evaluation of the methodological quality for the primary outcome of change in HbA1c from baseline to 26 weeks in the Frias et al. 2018 randomized controlled trial:

Article ID: Frias et al., 2018

Outcome Name: Change in HbA1c from baseline to 26 weeks

1. Was the allocation sequence adequately generated?

- Definitely yes

- The study states "Participants who met the enrolment criteria were randomly allocated (1:1:1:1:1:1) to one of the six parallel treatment groups by use of an interactive web response system with three stratification variables: baseline HbA1c (<8·5% or ≥8·5%), metformin use (yes or no), and BMI (<30 kg/m2 or ≥30 kg/m2)." Using an interactive web response system indicates adequate generation of the allocation sequence.

2. Was the allocation adequately concealed?

- Definitely yes

- The study states "To ensure masking of patients and investigators from treatment identification due to the differences in volumes and titration regimes across the treatment groups, the patients in the placebo and dulaglutide groups were further randomised to the four LY3298176 dosing groups (1, 5, 10, and 15 mg)." Further randomization to different dosing groups indicates adequate concealment of the allocation.

3. Blinding: Was knowledge of the allocated interventions adequately prevented?

3a. Were patients blinded?

- Definitely yes

- The study states it was a "double-blind" study. Further randomization was done to conceal treatment allocation from patients.

© 2024 Lai H et al. *JAMA Network Open.*

3b. Were healthcare providers blinded?

- Definitely yes

- The study states it was a "double-blind" study. Further randomization was done to conceal treatment allocation from investigators.

3c. Were data collectors blinded?

- Probably yes

- The study does not explicitly state whether data collectors were blinded, but the double-blind design implies they likely were.

3d. Were outcome assessors blinded?

- Probably yes

- The study does not explicitly state whether outcome assessors were blinded, but the double-blind design implies they likely were.

3e. Were data analysts blinded?

- Probably yes

- The study does not explicitly state whether data analysts were blinded, but the double-blind design implies they likely were.

4. Was loss to follow-up infrequent?

- Probably yes

- 258/316 (82%) participants completed 26 weeks of treatment. The study states "The proportion of participants completing treatment was similar across the groups (range 82–86%), except for the 15 mg LY3298176 group, which had the lowest number of patients (66%) completing therapy." Although the 15 mg group had more dropouts, overall the follow-up rate was high.

5. Are reports of the study free of selective outcome reporting?

- Probably yes

- The study protocol is not available, but the pre-specified primary and secondary outcomes are reported as expected in the methods section. There is no indication of selective reporting.

6. Was the study apparently free of other problems that could put it at a risk of bias?

© 2024 Lai H et al. *JAMA Network Open.*

- Definitely yes

- The study appears to be free of other sources of bias.

© 2024 Lai H et al. *JAMA Network Open.*

ID: Gao, 2020

First response:

Here is my evaluation of the risk of bias for the RCT by Gao et al., 2020 for the outcome of change in HbA1c at 24 weeks:

Article ID: Gao, 2020

Outcome Name: Change in HbA1c at 24 weeks

1. Was the allocation sequence adequately generated?

- Probably no

- No details are provided on how the randomization sequence was generated. The article only states "Eligible patients were equally randomized to the following three groups: placebo, PEX168 100 μg and PEX168 200 μg."

2. Was the allocation adequately concealed?

- Probably no

- No details are provided on allocation concealment.

3. Blinding: Was knowledge of the allocated interventions adequately prevented?

3a. Were patients blinded?

- Probably yes

- The study is described as double-blind and placebo-controlled. Placebo injections were used to match the PEX168 injections. This suggests attempts were made to blind patients.

3b. Were healthcare providers blinded?

- Probably yes

- The study is described as double-blind, suggesting healthcare providers were blinded.

3c. Were data collectors blinded?

- Probably yes

© 2024 Lai H et al. *JAMA Network Open.*

- The study is described as double-blind, suggesting data collectors were blinded.

3d. Were outcome assessors blinded?

- Probably yes

- The study is described as double-blind, suggesting outcome assessors were blinded.

3e. Were data analysts blinded?

- Probably yes

- The study is described as double-blind, suggesting data analysts were blinded.

4. Was loss to follow-up infrequent?

- Probably yes

- 573 patients were randomized and 533 were included in the full analysis set. The dropout rate was around 7%, which suggests a low risk of bias.

5. Are reports of the study free of selective outcome reporting?

- Probably no

- The study protocol is not available to compare reported outcomes against pre-specified outcomes.

6. Was the study apparently free of other problems that could put it at a risk of bias?

- Probably yes

- The study appears to be free of other sources of bias based on the information provided.

© 2024 Lai H et al. *JAMA Network Open.*

ID: Gao, 2020

Second response:

Thank you for providing the detailed guidelines and instructions on evaluating the methodological quality of the randomized controlled trial (RCT) from Gao et al., 2020. Based on the information provided in the article, here is my assessment for the primary outcome of change in HbA1c at 24 weeks:

Article ID: Gao et al., 2020

Outcome Name: Change in HbA1c at 24 weeks

1. Was the allocation sequence adequately generated?

- Probably yes

- The article states "Eligible patients were equally randomized to the following three groups: placebo, PEX168 100 μg and PEX168 200 μg. Patients who were randomized to the placebo group were further randomized to receive 100 and 200 μg of PEX168 for 28 weeks after receiving placebo for the first 24 weeks." This suggests some form of randomization was used, though the exact method is not specified.

2. Was the allocation adequately concealed?

- Probably yes

- The article states "Randomization and drug container assignment were managed using a central randomization system." Central allocation generally implies adequate concealment.

3. Blinding: Was knowledge of the allocated interventions adequately prevented?

3a. Were patients blinded?

- Probably yes

- The study is described as double-blind. The article states "PEX168 and placebo injections were provided by the same manufacturer (Jiangsu Hansoh Pharmaceutical Group Co., Ltd., Jiangsu,

© 2024 Lai H et al. *JAMA Network Open.*

China) with exactly the same packaging, labelling, appearance, taste, smell and time of administration." This suggests efforts were made to blind patients.

3b. Were healthcare providers blinded?

- Probably yes

- The study is described as double-blind, which implies blinding of healthcare providers.

3c. Were data collectors blinded?

- Probably yes

- The study is described as double-blind, which implies blinding of data collectors.

3d. Were outcome assessors blinded?

- Probably yes

- The study is described as double-blind, which implies blinding of outcome assessors.

3e. Were data analysts blinded?

- Probably yes

- The article states "From the beginning of randomization to the locking of the database, patients, investigators, evaluators and data analysts were blinded to the treatment."

4. Was loss to follow-up (missing outcome data) infrequent?

- Probably yes

- 174/179 (97.2%) in the PEX168 100 μg group, 173/175 (98.9%) in the PEX168 200 μg group, and 176/179 (98.3%) in the placebo group completed the study. The dropout rate is low and balanced across groups.

5. Are reports of the study free of selective outcome reporting?

- Probably yes

- The pre-specified primary and secondary outcomes are reported as described in the methods.

© 2024 Lai H et al. *JAMA Network Open.*

6. Was the study apparently free of other problems that could put it at a risk of bias?

- Probably yes
- No other apparent biases noted.

© 2024 Lai H et al. *JAMA Network Open.*

ID: Ikonomidis, 2020

First response:

Here are the evaluation results for the Ikonomidis et al. 2020 RCT:

Article ID: Ikonomidis, 2020

Outcome Name: Changes in endothelial glycocalyx thickness

1. Was the allocation sequence adequately generated?

- Definitely yes

- The study states "Patients were recruited...and they were randomized to receive..." indicating that randomization was used to generate the allocation sequence.

2. Was the allocation adequately concealed?

- Probably yes

- The study does not provide details on allocation concealment, but states that patients were randomized, which implies some level of concealment.

3. Blinding: Was knowledge of the allocated interventions adequately prevented?

3a. Were patients blinded?

- Definitely no

- The study states "Patients and clinicians prescribing the treatment were blinded to the results of the vascular and cardiac function tests" but it is not feasible to blind patients to the treatments being compared.

3b. Were healthcare providers blinded?

- Definitely no

- The study states "Patients and clinicians prescribing the treatment were blinded..." but clinicians could not be blinded to the treatment they were prescribing.

© 2024 Lai H et al. *JAMA Network Open.*

3c. Were data collectors blinded?

- Definitely yes
- The study states "All studies were analyzed by 2 observers...blinded to clinical and laboratory data."

3d. Were outcome assessors blinded?

- Definitely yes
- The study states "All studies were analyzed by 2 observers...blinded to clinical and laboratory data."

3e. Were data analysts blinded?

- Definitely yes
- The study states "All studies were analyzed by 2 observers...blinded to clinical and laboratory data."

4. Was loss to follow-up (missing outcome data) infrequent?

- Probably yes
- 20 out of 180 participants did not complete the study. This dropout rate is 11%, which is acceptable. The study does not provide reasons for dropout in each group.

5. Are reports of the study free of selective outcome reporting?

- Probably yes
- The study protocol is not available but the published report includes results for the pre-specified outcomes related to endothelial function. There is no clear evidence of selective reporting.

6. Was the study apparently free of other problems that could put it at a risk of bias?

- Probably yes
- The study appears to be free of other sources of bias.

ID: Ikonomidis, 2020

Second response:

Article ID: Ikonomidis et al., 2020

Outcome Name: Left ventricularglobal longitudinal strain

1. Was the allocation sequence adequately generated?

- Response: Probably no

- Reason: The study states that patients were randomized to the treatment groups, but details of sequence generation are not provided.

2. Was the allocation adequately concealed?

- Response: Probably no

- Reason: Details of allocation concealment are not provided.

3. Blinding: Was knowledge of the allocated interventions adequately prevented?

3a. Were patients blinded?

- Response: Probably no

- Reason: The study does not report on blinding of patients.

3b. Were healthcare providers blinded?

- Response: Probably no

- Reason: The study does not report on blinding of healthcare providers.

3c. Were data collectors blinded?

- Response: Probably no

- Reason: The study does not report on blinding of data collectors.

3d. Were outcome assessors blinded?

- Response: Probably no

- Reason: The study does not report on blinding of outcome assessors.

3e. Were data analysts blinded?

- Response: Probably no

© 2024 Lai H et al. *JAMA Network Open.*

- Reason: The study does not report on blinding of data analysts.

4. Was loss to follow-up (missing outcome data) infrequent?

- Response: Probably yes

- Reason: 20 out of 180 participants (11%) did not complete the study. Reasons for dropout are provided and appear balanced between groups.

5. Are reports of the study free of selective outcome reporting?

- Response: Probably yes

- Reason: The study protocol is not available, but the published report includes the expected outcomes.

6. Was the study apparently free of other problems that could put it at a risk of bias?

- Response: Probably yes

- Reason: The study appears to be free of other sources of bias based on the information provided.

Please let me know if you would like me to clarify or expand on any part of this assessment. I'm happy to revise my evaluation as needed.

© 2024 Lai H et al. *JAMA Network Open.*

ID: Nahra, 2021

First response:

Article ID: Nahra, 2021

Outcome Name: Change in HbA1c at week 14

1. **Randomization Sequence Generation**

- Assessment: Definitely yes.

- Rationale: The article explicitly mentions that "Participants were randomly assigned... computer-generated randomized sequence." This indicates proper random sequence generation using computer-generated random numbers.

2. **Allocation Concealment**

- Assessment: Probably yes.

- Rationale: While the article does not provide specific details about allocation concealment, the use of computer-generated randomization suggests that allocation was likely adequately concealed.

3. **Blinding of Interventions**

3a. **Blinding of Patients**

- Assessment: Definitely yes.

- Rationale: The article states, "Administration of cotadutide or placebo... was double-blind," confirming that patients receiving cotadutide or placebo were blinded.

3b. **Blinding of Healthcare Providers**

- Assessment: Probably yes for cotadutide and placebo groups, Definitely no for liraglutide group.

- Rationale: The article indicates that cotadutide and placebo administration was double-blinded, while liraglutide was open-label. This suggests healthcare providers were likely blinded for cotadutide and placebo groups but not for the liraglutide group.

© 2024 Lai H et al. *JAMA Network Open.*

3c. **Blinding of Data Collectors**

- Assessment: Probably yes.

- Rationale: The article does not explicitly mention blinding of data collectors. However, since it was a double-blind trial for cotadutide and placebo groups, it is likely that data collectors were also blinded.

3d. **Blinding of Outcome Assessors**

- Assessment: Probably yes.

- Rationale: The article does not provide information about the blinding of outcome assessors. However, since this was a double-blind trial for cotadutide and placebo groups, it is probable that outcome assessors were also blinded.

3e. **Blinding of Data Analysts**

- Assessment: Probably yes.

- Rationale: The article does not provide information about the blinding of data analysts. However, since this was a double-blind trial for cotadutide and placebo groups, it is likely that data analysts were also blinded.

4. **Loss to Follow-up**

- Assessment: Probably yes.

- Rationale: The article does not report specific numbers for loss to follow-up at 14 weeks. However, Fig 1 illustrates that 4-5% of participants in each group discontinued the study by week 14. This dropout rate appears low and unlikely to substantially affect the results.

5. **Selective Outcome Reporting**

- Assessment: Probably yes.

- Rationale: The study protocol is not available for verification, but the pre-specified primary outcome of change in HbA1c at week 14 appears to be fully reported as planned. There is no indication of selective reporting in the presented data.

© 2024 Lai H et al. *JAMA Network Open.*

6. **Risk of Bias from Other Sources**

- Assessment: Definitely yes.
- Rationale: The study appears to be free of other sources of bias as reported in the article.

In summary, this randomized controlled trial appears to have a low risk of bias for the outcome of change in HbA1c at week 14 based on the information provided. The use of computer-generated randomization, double-blinding for cotadutide and placebo groups, and a low dropout rate contribute to the overall low risk of bias.

© 2024 Lai H et al. *JAMA Network Open.*

ID: Nahra, 2021

Second response:

Based on the criteria outlined, here is my evaluation of the randomized controlled trial by Nahra et al. (2021) for the outcome of change in HbA1c at 14 weeks:

Nahra et al., 2021

Outcome Name: Change in HbA1c at 14 weeks

1. Was the allocation sequence adequately generated?

   - Probably yes

   - "Participants were randomly assigned (overall ratio 4:9:9:4:4) to receive once-daily subcutaneous injections of cotadutide at 100 µg, 200 µg, or 300 µg; liraglutide 1.8 mg; or placebo." This suggests adequate random sequence generation, but details are not provided to definitively assess the method.

2. Was the allocation adequately concealed?

   - Probably yes

   - The methods state "Assignment to treatment groups was determined by a computer-generated randomized sequence with use of interactive web response systems and interactive voice-response systems." The use of centralized allocation makes adequate concealment probable but details are not provided to definitively assess the method.

3. Blinding: Was knowledge of the allocated interventions adequately prevented?

   - 3a. Were patients blinded? Probably yes. Patients received injections from blinded prefilled syringes or open-label pens, making blinding of cotadutide groups probable but blinding of liraglutide group unlikely.

   - 3b. Were healthcare providers blinded? Probably yes. The article indicates that cotadutide and placebo administration was double-blinded.

   - 3c. Were data collectors blinded? Probably yes. No details, but blinding of data collectors is typical in double-blind trials.

   - 3d. Were outcome assessors blinded? Probably yes. No details, but blinding of outcome assessors is typical in double-blind trials.

- 3e. Were data analysts blinded? Probably yes. No details, but blinding of data analysts is typical in double-blind trials.

4. Was loss to follow-up (missing outcome data) infrequent?

- Probably yes
- Dropout rates were 4-11% across groups at 14 weeks. This moderate dropout rate across groups suggests missing data were probably not a major issue for this outcome at this time point.

5. Are reports of the study free of selective outcome reporting?

- Probably yes
- The pre-specified outcomes appear to be reported, but the full study protocol is not available to verify this definitively.

6. Was the study apparently free of other problems that could put it at a risk of bias?

- Probably yes
- No other apparent biases noted.

© 2024 Lai H et al. *JAMA Network Open.*

ID: Seino, 2015

First response:

Here are the evaluation results for Seino et al. 2015:

Article ID: Seino, 2015

Outcome Name: Change in HbA1c from baseline to Week 24

1. Was the allocation sequence adequately generated?

- Response: Probably no
- Reason: The article states "patients were randomly assigned to receive luseogliflozin 2.5 mg or placebo". However, no details are provided on how the random sequence was generated.

2. Was allocation adequately concealed?

- Response: Probably no
- Reason: The article does not mention any details about allocation concealment.

3. Blinding: Was knowledge of the allocated interventions adequately prevented?

3a. Were patients blinded?

- Response: Probably no
- Reason: The article only states this was a "double-blind" study, but does not provide any details about who specifically was blinded. Therefore, there is insufficient information to determine if patients were actually blinded.

3b. Were healthcare providers blinded?

- Response: Probably no
- Reason: The article only states this was a "double-blind" study, but does not provide any details about who specifically was blinded. Therefore, there is insufficient information to determine if healthcare providers were actually blinded.

© 2024 Lai H et al. *JAMA Network Open.*

3c. Were data collectors blinded?

- Response: Probably no

- Reason: The article only states this was a "double-blind" study, but does not provide any details about who specifically was blinded. Therefore, there is insufficient information to determine if data collectors were actually blinded.

3d. Were outcome assessors blinded?

- Response: Probably no

- Reason: The article only states this was a "double-blind" study, but does not provide any details about who specifically was blinded. Therefore, there is insufficient information to determine if outcome assessors were actually blinded.

3e. Were data analysts blinded?

- Response: Probably no

- Reason: The article only states this was a "double-blind" study, but does not provide any details about who specifically was blinded. Therefore, there is insufficient information to determine if data analysts were actually blinded.

4. Was loss to follow-up infrequent?

- Response: Definitely yes

- Reason: The article states "Efficacy and safety assessments were performed in all subjects who received the study drug at least once and underwent examination/observation for the post-administration assessment." This suggests there was no loss to follow-up.

5. Are reports of the study free of selective outcome reporting?

- Response: Definitely yes

- Reason: The pre-specified outcomes listed in the methods section (changes in HbA1c, FPG, body weight) are reported in the results.

6. Was the study apparently free of other problems that could put it at a risk of bias?

© 2024 Lai H et al. *JAMA Network Open.*

- Response: Definitely yes

- Reason: No other apparent sources of bias were identified.

© 2024 Lai H et al. *JAMA Network Open.*

ID: Seino, 2015

Second response:

Here is the evaluation for the Seino et al. 2015 RCT:

Article ID: Seino et al., 2015

Outcome Name: Change in HbA1c

1. Was the allocation sequence adequately generated?

- Definitely yes

- "Patients were randomized in a ratio of 2:1 to receive either luseogliflozin 2.5 mg or placebo (i.e. glimepiride alone) before breakfast once daily." This indicates that an adequate random sequence generation method was used.

2. Was the allocation adequately concealed?

- Probably yes

- The paper does not provide details on allocation concealment methods. However, it mentions that this was a "randomized, double-blind, parallel-group comparative study", suggesting attempts were likely made to conceal allocation.

3. Blinding: Was knowledge of the allocated interventions adequately prevented?

3a. Were patients blinded?

- Probably yes

- The study is described as double-blind, suggesting patients were likely blinded, although details are not provided.

3b. Were healthcare providers blinded?

- Probably yes

© 2024 Lai H et al. *JAMA Network Open.*

- The study is described as double-blind, suggesting healthcare providers were likely blinded, although details are not provided.

3c. Were data collectors blinded?

- Probably yes

- The study is described as double-blind, suggesting data collectors were likely blinded, although details are not provided.

3d. Were outcome assessors blinded?

- Probably yes

- The study is described as double-blind, suggesting outcome assessors were likely blinded, although details are not provided.

3e. Were data analysts blinded?

- Probably yes

- The study is described as double-blind, suggesting data analysts were likely blinded, although details are not provided.

4. Was loss to follow-up (missing outcome data) infrequent?

- Definitely yes

- In Fig. 1, 222 patients were randomized, 150 in the luseogliflozin group and 72 in the placebo group. At week 24, data was available for 147 patients in the luseogliflozin group and 71 in the placebo group. The dropout rate is less than 10% and balanced between groups.

5. Are reports of the study free of selective outcome reporting?

- Probably yes

- The study protocol is not available, but the pre-specified outcomes of HbA1c, FPG, and body weight are reported as expected.

6. Was the study apparently free of other problems that could put it at a risk of bias?

© 2024 Lai H et al. *JAMA Network Open.*

- Definitely yes

- The study appears to be free of other sources of bias.

© 2024 Lai H et al. *JAMA Network Open.*

ID: Taskinen, 2011

First response:

Here are the risk of bias assessments for the Taskinen et al. 2011 randomized controlled trial:

Outcome Name: Change in HbA1c from baseline after 24 weeks of treatment

1. Was the allocation sequence adequately generated?

- Definitely yes

- "Patients were randomized in a 3:1 ratio to linagliptin or placebo. This allocation was stratified by the level of glycemic control at the start of the placebo run-in (HbA1c <8.5% or ≥8.5%) and according to the use of monotherapy vs. combination therapy at enrollment." This indicates adequate generation of the allocation sequence using stratification.

2. Was the allocation adequately concealed?

- Probably no

- The method of allocation concealment is not described. However, since allocation was performed using stratification, it was likely adequately concealed.

3. Blinding: Was knowledge of the allocated interventions adequately prevented?

3a. Were patients blinded?

- Probably yes

- The study is described as double-blind, but details of patient blinding are not provided.

3b. Were healthcare providers blinded?

- Probably yes

- The study is described as double-blind, but details of healthcare provider blinding are not provided.

3c. Were data collectors blinded?

- Probably yes

- The study is described as double-blind, but details of data collector blinding are not provided.

© 2024 Lai H et al. *JAMA Network Open.*

3d. Were outcome assessors blinded?

- Probably yes

- The study is described as double-blind, but details of outcome assessor blinding are not provided.

3e. Were data analysts blinded?

- Probably yes

- The study is described as double-blind, but details of data analyst blinding are not provided.

4. Was loss to follow-up infrequent?

- Definitely yes

- 624 of the 688 patients (91%) completed the 24 week study. The dropout rate was less than 10%.

5. Are reports of the study free of selective outcome reporting?

- Probably yes

- The study protocol is not available, but the published report includes the pre-specified primary and secondary outcomes.

6. Was the study apparently free of other problems that could put it at a risk of bias?

- Definitely yes

- The study appears to be free of other sources of bias.

© 2024 Lai H et al. *JAMA Network Open.*

ID: Taskinen, 2011

Second response:

Here are the evaluation results for the Taskinen et al. 2011 study:

Article ID: Taskinen et al., 2011

Outcome Name: Change in HbA1c

1. Was the allocation sequence adequately generated?

- Definitely yes

- "Patients were randomized to linagliptin 5 mg once daily or placebo add-on to metformin in a 3:1 ratio. This allocation was stratified by the level of glycaemic control at the start of the placebo run-in..." (page 69). Randomization with stratification indicates adequate sequence generation.

2. Was allocation adequately concealed?

- Probably yes

- No details provided on how allocation concealment was ensured. However, this was described as a "double-blind, placebo-controlled" study, suggesting attempts were probably made to conceal allocation.

3. Blinding: Was knowledge of the allocated interventions adequately prevented?

- 3a. Were patients blinded?

    - Probably yes

    - No details provided but described as "double-blind", suggesting patients were likely blinded.

- 3b. Were healthcare providers blinded?

    - Probably yes

    - No details provided but described as "double-blind", suggesting healthcare providers were likely blinded.

- 3c. Were data collectors blinded?

    - Probably yes

© 2024 Lai H et al. *JAMA Network Open.*

- No details provided but described as "double-blind", suggesting data collectors were likely blinded.

- 3d. Were outcome assessors blinded?

  - Probably yes

  - No details provided but described as "double-blind", suggesting outcome assessors were likely blinded.

- 3e. Were data analysts blinded?

  - Probably yes

  - No details provided but described as "double-blind", suggesting data analysts were likely blinded.

4. Was loss to follow-up infrequent?

- Probably yes

- 624/688 (91%) of patients in the full analysis set completed the study. Follow-up rate was >90%, suggesting infrequent loss to follow-up.

5. Are reports of the study free of selective outcome reporting?

- Probably yes

- The study protocol is not available, but the pre-specified primary outcome (change in HbA1c) and other expected outcomes are reported. There is no evidence of selective reporting.

6. Was the study apparently free of other problems that could put it at a risk of bias?

- Probably yes

- The study appears to be free of other sources of bias.

© 2024 Lai H et al. *JAMA Network Open.*

ID: Yabiku, 2017

First response:

Here are the risk of bias evaluations for the outcome "Change in HbA1c from baseline after 24 weeks of treatment" reported in the RCT by Taskinen et al., 2011:

Article ID: Taskinen, 2011

Outcome Name: Change in HbA1c from baseline after 24 weeks of treatment

1. Was the allocation sequence adequately generated?

- Probably no

- The method of sequence generation is not described.

2. Was allocation adequately concealed?

- Probably no

- The method of allocation concealment is not described. With no information provided, it is difficult to make a positive judgement about adequate concealment.

3. Blinding: Was knowledge of the allocated interventions adequately prevented?

3a. Were patients blinded?

- Probably no

- The study states it was double-blind, but does not provide details on blinding of patients. With insufficient information, it is difficult to make a positive judgement.

3b. Were healthcare providers blinded?

- Probably no

- The study states it was double-blind, but does not provide details on blinding of healthcare providers. With insufficient information, it is difficult to make a positive judgement.

© 2024 Lai H et al. *JAMA Network Open.*

3c. Were data collectors blinded?

- Probably no

- The study does not mention blinding of data collectors. With no information provided, it is difficult to make a positive judgement.

3d. Were outcome assessors blinded?

- Probably yes

- The study states that HbA1c measurements were carried out by a central laboratory, which implies outcome assessors were likely blinded.

3e. Were data analysts blinded?

- Probably no

- The study does not mention blinding of data analysts. With no information provided, it is difficult to make a positive judgement.

4. Was loss to follow-up infrequent?

- Probably yes

- 89% of patients in the placebo group and 91% in the linagliptin group completed the 24 week study. The dropout rate appears low enough to not likely impact results.

5. Are reports free of selective outcome reporting?

- Probably yes

- The outcomes reported match those listed in the methods section as being pre-specified. Protocol is not available to verify further. But selective reporting seems unlikely.

6. Was the study apparently free of other problems that could put it at risk of bias?

- Probably yes

- The study appears free of other sources of bias based on the information provided.

© 2024 Lai H et al. *JAMA Network Open.*

ID: Yabiku, 2017

Second response:

Based on the information provided in the RCT report, here is my evaluation of the methodological quality for the outcome "Changes in the liver-to-spleen ratio on computed tomography":

Article ID: Yabiku et al., 2017

Outcome Name: Change in HbA1c from baseline after 24 weeks of treatment

1. Was the allocation sequence adequately generated?

- Probably yes

- The paper states "Patients with NAFLD were block randomized, with the block size set at 4 patients, using random-number generation software, into 4 groups." Using random number generation indicates adequate sequence generation, although more details on the specific software and process used would be needed to definitively judge this as adequate.

2. Was the allocation adequately concealed?

- Probably no

- The paper does not mention any methods used to conceal the allocation sequence. Without this information, allocation concealment cannot be confirmed.

3. Blinding: Was knowledge of the allocated interventions adequately prevented?

3a. Were patients blinded?

- Probably no

- The paper states "All treatments were open label (no single or double blind)." With open-label treatment, patients were aware of the interventions.

3b. Were healthcare providers blinded?

- Probably no

- As an open-label study, healthcare providers were aware of the interventions.

3c. Were data collectors blinded?

© 2024 Lai H et al. *JAMA Network Open.*

- Probably no

- The paper does not mention blinding of data collectors.

3d. Were outcome assessors blinded?

- Probably no

- The paper does not mention blinding of outcome assessors.

3e. Were data analysts blinded?

- Probably no

- The paper does not mention blinding of data analysts.

4. Was loss to follow-up (missing outcome data) infrequent?

- Probably yes

- The paper states "Three patients in the metformin group, 4 patients in the sitagliptin group, 1 patient in the pioglitazone group, and 7 patients in the control group (no OAD) were lost to follow-up." The follow-up rates were over 90% in each group, indicating infrequent missing outcome data.

5. Are reports of the study free of selective outcome reporting?

- Probably yes

- The pre-specified outcomes listed in the trial registration appear to be reported. However, without access to the full study protocol, selective outcome reporting cannot be definitively ruled out.

6. Was the study apparently free of other problems that could put it at a risk of bias?

- Probably yes

- No other apparent biases were identified based on the information provided.

© 2024 Lai H et al. *JAMA Network Open.*

ID: Allen, 1987

First response:

Based on the information provided in the Allen 1987 study, here is my assessment of the risk of bias for the outcome of sleep latency:

Article ID: Allen 1987

Outcome Name: Sleep Latency

1. Was the allocation sequence adequately generated?

- Probably no

- The method of sequence generation is not described, but patients were "randomized" to treatment groups. However, no details are provided on how randomization was carried out.

2. Was the allocation adequately concealed?

- Probably no

- The method of allocation concealment is not described.

3. Blinding: Was knowledge of the allocated interventions adequately prevented?

3a. Were patients blinded?

- Probably yes

- The study is described as "double-blind", suggesting patients were blinded. However, no details on blinding methods are provided.

3b. Were healthcare providers blinded?

- Probably yes

- The study is described as "double-blind", suggesting healthcare providers were blinded. However, no details on blinding methods are provided.

© 2024 Lai H et al. *JAMA Network Open.*

3c. Were data collectors blinded?

- Probably no
- Blinding of data collectors is not described.

3d. Were outcome assessors blinded?

- Probably no
- Blinding of outcome assessors is not described.

3e. Were data analysts blinded?

- Probably no
- Blinding of data analysts is not described.

4. Was loss to follow-up (missing outcome data) infrequent?

- Probably yes
- 17 patients out of 175 (9.7%) did not complete phase 1 of the study. Reasons for dropout appear evenly distributed between groups. This proportion of missing data may not have an important impact on effect estimates.

5. Are reports of the study free of selective outcome reporting?

- Probably yes
- The study protocol is not available, but the pre-specified outcomes appear to be reported as expected.

6. Was the study apparently free of other problems that could put it at risk of bias?

- Probably yes
- The study appears to be free of other sources of bias based on the information provided.

© 2024 Lai H et al. *JAMA Network Open.*

ID: Allen, 1987

Second response:

Here is the risk of bias assessment for the outcome of hypnotic efficacy (increased total sleep time) in the RCT by Allen et al. 1987:

Article ID: Allen, 1987

Outcome Name: Hypnotic efficacy (increased total sleep time)

1. Was the allocation sequence adequately generated?

- Probably yes

- The article states that patients were "randomized" to receive midazolam or temazepam but does not provide details on how the allocation sequence was generated. However, this was a double-blind, parallel group study conducted at multiple centers, suggesting that some adequate randomization method was likely used.

2. Was the allocation adequately concealed?

- Probably no

- The article does not mention any methods used to conceal the allocation sequence, so there is insufficient information to judge this positively.

3. Blinding: Was knowledge of the allocated interventions adequately prevented?

3a. Were patients blinded?

- Probably yes

- The study is described as double-blind, suggesting patients were likely blinded, but details are not provided.

3b. Were healthcare providers blinded?

- Probably yes

© 2024 Lai H et al. *JAMA Network Open.*

- The study is described as double-blind, suggesting healthcare providers were likely blinded, but details are not provided.

3c. Were data collectors blinded?

- Probably no
- The article does not mention blinding of data collectors.

3d. Were outcome assessors blinded?

- Probably no
- The article does not mention blinding of outcome assessors.

3e. Were data analysts blinded?

- Probably no
- The article does not mention blinding of data analysts.

4. Was loss to follow-up (missing outcome data) infrequent?

- Probably yes
- 34 patients out of 175 (19.4%) withdrew from the study, with reasonably balanced reasons for withdrawal between groups. This rate of missing data is unlikely to impact results.

5. Are reports of the study free of selective outcome reporting?

- Probably yes
- The study protocol is not available, but the pre-specified efficacy outcomes appear to be fully reported.

6. Was the study apparently free of other problems that could put it at a risk of bias?

- Probably yes
- No other apparent sources of bias. The study appears to be a well-conducted double-blind RCT.

© 2024 Lai H et al. *JAMA Network Open.*

Please let me know if you would like me to assess the risk of bias for any other outcomes reported in this RCT. I can provide evaluations for multiple outcomes using the same format.

© 2024 Lai H et al. *JAMA Network Open.*

ID: Black, 2017

First response:

Here is the evaluation for the primary outcome of wake time after sleep onset (WASO) reported in the Black et al. 2017 RCT:

Article ID: Black et al., 2017

Outcome Name: Wake time after sleep onset (WASO)

1. **Was the allocation sequence adequately generated?**

- Response: Probably no

- Reason: No details were provided on how the allocation sequence was generated. The paper only states "Patients meeting study entry criteria were randomized 1:1:1:1 to one of four study oral treatments."

2. **Was the allocation adequately concealed?**

- Response: Probably no

- Reason: No details were provided on how allocation was concealed.

3. **Blinding: Was knowledge of the allocated interventions adequately prevented?**

3a. **Were patients blinded?**

- Response: Probably yes

- Reason: The paper states "To maintain blinding, almorexant and its matching placebo were indistinguishable and identically packaged. So too were the reference drug (zolpidem) and its matching placebo." This suggests patients were likely blinded, but details are not provided to confirm.

3b. **Were healthcare providers blinded?**

- Response: Probably no

- Reason: No details provided.

© 2024 Lai H et al. *JAMA Network Open.*

3c. **Were data collectors blinded?**

- Response: Probably no

- Reason: No details provided.

3d. **Were outcome assessors blinded?**

- Response: Probably no

- Reason: No details provided.

3e. **Were data analysts blinded?**

- Response: Probably no

- Reason: No details provided.

4. **Was loss to follow-up (missing outcome data) infrequent?**

- Response: Probably yes

- Reason: The paper states "In addition, 44 patients (16 on placebo, 13 on almorexant 100 mg, 6 on almorexant 200 mg, and 9 on zolpidem) did not complete the study." This represents a loss to follow-up rate of around 6%, which suggests infrequent missing outcome data.

5. **Are reports of the study free of selective outcome reporting?**

- Response: Probably no

- Reason: The study protocol is not available to verify that all pre-specified outcomes were reported. Based on the information provided, it is unclear if selective reporting occurred.

6. **Was the study apparently free of other problems that could put it at a risk of bias?**

- Response: Probably yes

- Reason: Based on the information provided, there do not appear to be other sources of bias. The study design and methodology seem reasonable.

© 2024 Lai H et al. *JAMA Network Open.*

Please let me know if you would like me to evaluate any other outcomes reported in this RCT. I'm happy to provide assessments for additional outcomes using the same format.

© 2024 Lai H et al. *JAMA Network Open.*

ID: Black, 2017

Second response:

Article ID: Black, 2017

Outcome Name: Objective WASO

1. Was the allocation sequence adequately generated?

- Response: Probably no

- Reason: The article states "Patients meeting study entry criteria were randomized 1:1:1:1 to one of four study oral treatments (placebo, almorexant 100 mg, almorexant 200 mg, or zolpidem 10 mg)." However, no details are provided on how the randomization sequence was generated.

2. Was allocation adequately concealed?

- Response: Probably no

- Reason: The article does not provide details on allocation concealment.

3. Blinding: Was knowledge of the allocated interventions adequately prevented?

3a. Were patients blinded?

- Response: Probably yes

- Reason: The article states this was a "double-blind" study, and blinding of participants is likely as medications were matched placebos.

3b. Were healthcare providers blinded?

- Response: Probably yes

- Reason: The article states this was a "double-blind" study, so blinding of providers is likely.

3c. Were data collectors blinded?

- Response: Probably yes

- Reason: The article states polysomnography data was "scored centrally" suggesting blinding of data collectors.

© 2024 Lai H et al. *JAMA Network Open.*

3d. Were outcome assessors blinded?

- Response: Probably yes

- Reason: The article states this was a "double-blind" study, so blinding of outcome assessors is likely.

3e. Were data analysts blinded?

- Response: Probably yes

- Reason: The article states this was a "double-blind" study, so blinding of data analysts is likely.

4. Was loss to follow-up infrequent?

- Response: Probably yes

- Reason: 93.8% of patients completed the study. The dropout rate is less than 20% in all groups.

5. Are reports of the study free of selective outcome reporting?

- Response: Probably yes

- Reason: The protocol is not available but the pre-specified primary and secondary outcomes appear to be reported as expected.

6. Was the study apparently free of other problems that could put it at a risk of bias?

- Response: Probably yes

- Reason: The study appears free of other sources of bias based on the information provided.

© 2024 Lai H et al. *JAMA Network Open.*

ID: Fan, 2017

First response:

Thank you for providing the detailed guidelines and examples for evaluating the risk of bias in randomized controlled trials (RCTs). Based on the instructions, here is my assessment of the RCT by Fan et al. (2017):

Article ID: Fan, 2017

Outcome Name: Total sleep time (sTST)

1. Was the allocation sequence adequately generated?

- Response: Probably yes

- Reason: The article states "Eligible subjects were randomly allocated to either the suvorexant or the placebo group using a computerized number generator in the stratified block randomization method in SAS (version 8.2; SAS Institute, Inc., Cary, NC, USA)." Using a computerized number generator indicates adequate sequence generation.

2. Was the allocation adequately concealed?

- Response: Probably yes

- Reason: The article states "The randomization was performed by a statistician from whom treatment allocation was masked. The treatment allocation was concealed in opaque sequentially numbered sealed envelopes containing the randomization assignments." Using opaque, sealed, sequentially numbered envelopes indicates adequate concealment.

3. Blinding:

3a. Were patients blinded?

- Response: Probably yes

- Reason: The article states "The patients, investigators, and study site personnel were blinded to the treatment allocation." This indicates attempts were made to blind patients.

3b. Were healthcare providers blinded?

© 2024 Lai H et al. *JAMA Network Open.*

- Response: Probably yes

- Reason: The article states "The patients, investigators, and study site personnel were blinded to the treatment allocation." This indicates attempts were made to blind healthcare providers.

3c. Were data collectors blinded?

- Response: Probably yes

- Reason: The article states "The patients, investigators, and study site personnel were blinded to the treatment allocation." This indicates attempts were made to blind data collectors.

3d. Were outcome assessors blinded?

- Response: Probably yes

- Reason: The article states "Individuals who were directly involved in the study (including the trial conduction and data analysis) did not have access to the randomization schedule until the trial was completed." This indicates outcome assessors were blinded.

3e. Were data analysts blinded?

- Response: Probably yes

- Reason: The article states "Individuals who were directly involved in the study (including the trial conduction and data analysis) did not have access to the randomization schedule until the trial was completed." This indicates data analysts were blinded.

4. Was loss to follow-up infrequent?

- Response: Probably yes

- Reason: 120 patients were randomized and 111 completed the study. The follow-up rate was >90%, indicating infrequent loss to follow-up.

5. Are reports of the study free of selective outcome reporting?

- Response: Probably no

- Reason: The study protocol is not available to verify there is no selective outcome reporting, but the pre-specified primary and secondary outcomes appear to be reported as expected.

© 2024 Lai H et al. *JAMA Network Open.*

6. Was the study apparently free of other problems that could put it at risk of bias?

- Response: Probably yes

- Reason: The study appears to be free of other sources of bias based on the information provided.

Does this assessment follow the provided guidelines correctly? Please let me know if I should modify or clarify any responses. I am happy to re-evaluate the study according to your specifications.

© 2024 Lai H et al. *JAMA Network Open.*

ID: Fan, 2017

Second response:

Article ID: Fan, 2017

Outcome Name: Total sleep time (sTST)

1. Was the allocation sequence adequately generated?

- Response: Probably yes

- Reason: The article states "Eligible subjects were randomly allocated to either the suvorexant or the placebo group using a computerized number generator in the stratified block randomization method in SAS (version 8.2; SAS Institute, Inc., Cary, NC, USA)."

2. Was the allocation adequately concealed?

- Response: Probably yes

- Reason: The article states "The randomization was performed by a statistician from whom treatment allocation was masked. The treatment allocation was concealed in opaque sequentially numbered sealed envelopes containing the randomization assignments." This suggests adequate allocation concealment.

3. Blinding: Was knowledge of the allocated interventions adequately prevented?

3a. Were patients blinded?

- Response: Probably yes

- Reason: The study is described as double-blind, suggesting patients were blinded.

3b. Were healthcare providers blinded?

- Response: Probably yes

- Reason: The study is described as double-blind, suggesting healthcare providers were blinded.

3c. Were data collectors blinded?

- Response: Probably yes

- Reason: The study is described as double-blind, suggesting data collectors were blinded.

© 2024 Lai H et al. *JAMA Network Open.*

3d. Were outcome assessors blinded?

- Response: Probably yes

- Reason: The study is described as double-blind, suggesting outcome assessors were blinded.

3e. Were data analysts blinded?

- Response: Probably yes

- Reason: The article states "Data analysis was performed by a study statistician who was blind to the treatment allocation."

4. Was loss to follow-up infrequent?

- Response: Probably yes

- Reason: 114/120 participants completed the study. The follow-up rate is greater than 90%, so loss to follow-up can be considered infrequent.

5. Are reports of the study free of selective outcome reporting?

- Response: Probably yes

- Reason: The study protocol is not available to verify there is no selective outcome reporting, but the pre-specified primary and secondary outcomes appear to be reported as expected.

6. Was the study apparently free of other problems that could put it at a risk of bias?

- Response: Probably yes

- Reason: The study appears to be free of other sources of bias based on the information provided.

© 2024 Lai H et al. *JAMA Network Open.*

ID: Lankford, 2012

First response:

Here are the risk of bias assessments for the article "Efficacy and safety of doxepin 6 mg in a four-week outpatient trial of elderly adults with chronic primary insomnia" by Lankford et al. 2012:

Article ID: Lankford, 2012

Outcome Name: Subjective total sleep time (sTST) at Week 1

1. Was the allocation sequence adequately generated?

- Probably yes

- The article states "Randomization numbers were issued to each study center on the treatment kits, which were shipped to each study center in blocks of four." Although the exact method of sequence generation is not described, the use of blocked randomization suggests adequate generation.

2. Was allocation adequately concealed?

- Probably yes

- The article states kits were shipped to study centers with randomization numbers. This suggests central allocation which would allow for adequate concealment.

3.a. Were patients blinded?

- Probably yes

- The study is described as double-blind, suggesting attempts were made to blind patients.

3.b. Were healthcare providers blinded?

- Probably yes

- The study is described as double-blind, suggesting attempts were made to blind providers.

3.c. Were data collectors blinded?

- Probably yes

- The study is described as double-blind, suggesting attempts were made to blind data collectors.

© 2024 Lai H et al. *JAMA Network Open.*

3.d. Were outcome assessors blinded?

- Probably yes

- The study is described as double-blind, suggesting attempts were made to blind outcome assessors.

3.e. Were data analysts blinded?

- Probably yes

- The study is described as double-blind, suggesting attempts were made to blind data analysts.

4. Was loss to follow-up infrequent?

- Probably yes

- 113/124 (91%) of placebo group and 124/130 (95%) of doxepin group completed the study. Dropout rates were less than 10% in both groups.

5. Are reports of the study free of selective outcome reporting?

- Probably yes

- The pre-specified primary outcome of sTST at Week 1 is reported. Other pre-specified secondary outcomes are also reported.

6. Was the study apparently free of other problems that could put it at a risk of bias?

- Probably yes

- No other apparent sources of bias. The study appears to be free of other problems that could put it at risk of bias.

Outcome Name: Latency to sleep onset (LSO) at Week 1

1. Was the allocation sequence adequately generated?

- Probably yes

© 2024 Lai H et al. *JAMA Network Open.*

- The article states "Randomization numbers were issued to each study center on the treatment kits, which were shipped to each study center in blocks of four." Although the exact method of sequence generation is not described, the use of blocked randomization suggests adequate generation.

2. Was allocation adequately concealed?

- Probably yes

- The article states kits were shipped to study centers with randomization numbers. This suggests central allocation which would allow for adequate concealment.

3.a. Were patients blinded?

- Probably yes

- The study is described as double-blind, suggesting attempts were made to blind patients.

3.b. Were healthcare providers blinded?

- Probably yes

- The study is described as double-blind, suggesting attempts were made to blind providers.

3.c. Were data collectors blinded?

- Probably yes

- The study is described as double-blind, suggesting attempts were made to blind data collectors.

3.d. Were outcome assessors blinded?

- Probably yes

- The study is described as double-blind, suggesting attempts were made to blind outcome assessors.

3.e. Were data analysts blinded?

- Probably yes

- The study is described as double-blind, suggesting attempts were made to blind data analysts.

4. Was loss to follow-up infrequent?

© 2024 Lai H et al. *JAMA Network Open.*

- Probably yes

- 113/124 (91%) of placebo group and 124/130 (95%) of doxepin group completed the study. Dropout rates were less than 10% in both groups.

5. Are reports of the study free of selective outcome reporting?

- Probably yes

- The pre-specified key secondary outcome of LSO at Week 1 is reported.

6. Was the study apparently free of other problems that could put it at a risk of bias?

- Probably yes

- No other apparent sources of bias. The study appears to be free of other problems that could put it at risk of bias.

© 2024 Lai H et al. *JAMA Network Open.*

ID: Lankford, 2012

Second response:

Based on the guidelines provided and the information in the article by Lankford et al. (2012), here are the risk of bias assessments for the outcome of wake after sleep onset (WASO):

Article ID: Lankford et al., 2012

Outcome Name: Wake after sleep onset (WASO)

1. Was the allocation sequence adequately generated?

- Response: Probably yes

- Reason: The article states "Eligible patients were assigned to one of two treatment groups (DXP 6 mg or placebo) in a 1:1 ratio according to a computer-generated randomization scheme." Computer-generated randomization is usually considered adequate.

2. Was allocation adequately concealed?

- Response: Probably no

- Reason: No statements are provided on allocation concealment.

3. Blinding: Was knowledge of the allocated interventions adequately prevented during the study?

3a. Were patients blinded?

- Response: Probably yes

- Reason: The study is described as double-blind, suggesting attempts were made to blind patients, though details are not provided. The outcome is unlikely to be influenced by lack of blinding.

3b. Were healthcare providers blinded?

- Response: Probably yes

- Reason: The study is described as double-blind, suggesting attempts were made to blind healthcare providers, though details are not provided. The outcome is unlikely to be influenced by lack of blinding.

3c. Were data collectors blinded?

© 2024 Lai H et al. *JAMA Network Open.*

- Response: Probably no

- Reason: No statements are provided on blinding of data collectors.

3d. Were outcome assessors blinded?

- Response: Probably no

- Reason: No statements are provided on blinding of outcome assessors.

3e. Were data analysts blinded?

- Response: Probably no

- Reason: No statements are provided on blinding of data analysts.

4. Was loss to follow-up infrequent?

- Response: Probably yes

- Reason: 93% of patients completed the study. Loss to follow-up appears to be infrequent.

5. Are reports of the study free of suggestion of selective outcome reporting?

- Response: Probably no

- Reason: The study protocol is not available to compare with the outcomes reported.

6. Was the study apparently free of other problems that could put it at a risk of bias?

- Response: Probably yes

- Reason: The study appears to be free of other sources of bias based on the information provided.

Does this summary accurately reflect the risk of bias assessments based on the guidelines and information provided? Please let me know if you would like me to modify or expand my explanations for any item.

© 2024 Lai H et al. *JAMA Network Open.*

ID: Mignot, 2022

First response:

Based on the information provided, here are the risk of bias assessments for the RCT "Safety and efficacy of daridorexant in patients with insomnia disorder: results from two multicentre, randomised, double-blind, placebo-controlled, phase 3 trials" by Mignot et al. (2022):

Outcome Name: Wake time after sleep onset (WASO)

1. Was the allocation sequence adequately generated?

- Response: Probably yes

- Reason: The article states "Randomisation was stratified by age (<65 years and ≥65 years), and treatment was allocated using an interactive response technology system. A randomisation list was generated for each study by Almac Clinical Technologies (Souderton, PA, USA) and remained confidential until after database lock." The use of an interactive response technology system suggests adequate generation of the allocation sequence.

2. Was allocation adequately concealed?

- Response: Probably yes

- Reason: The article states "Participants, investigators, site personnel, and sponsor-authorised personnel were unaware of treatment allocation." This suggests adequate concealment of allocation.

3. Blinding: Was knowledge of the allocated interventions adequately prevented during the study?

3a. Were participants blinded?

- Response: Probably yes

- Reason: The article states "Participants were masked to treatment during placebo run-in and run-out periods."

3b. Were healthcare providers blinded?

- Response: Probably yes

© 2024 Lai H et al. *JAMA Network Open.*

- Reason: The article states "Investigators, site personnel, and sponsor-authorised personnel were unaware of treatment allocation."

3c. Were data collectors blinded?

- Response: Probably yes
- Reason: The article states "Investigators, site personnel, and sponsor-authorised personnel were unaware of treatment allocation." Data collectors would be included in site personnel.

3d. Were outcome assessors blinded?

- Response: Probably yes
- Reason: The article states "Investigators, site personnel, and sponsor-authorised personnel were unaware of treatment allocation." Outcome assessors would be included in site personnel.

3e. Were data analysts blinded?

- Response: Probably yes
- Reason: The article states "Participants, investigators, site personnel, and sponsor-authorised personnel were unaware of treatment allocation." Data analysts would be included in sponsor-authorized personnel.

4. Was loss to follow-up infrequent?

- Response: Probably yes
- Reason: In study 1, 299/310 participants in the placebo group completed the month 1 assessment, and 283/310 completed the month 3 assessment. In the daridorexant 25 mg group 298/310 completed month 1 and 289/310 completed month 3. In the daridorexant 50 mg group 305/310 completed month 1 and 287/310 completed month 3. This indicates a low rate of loss to follow-up (less than 10% loss across groups and timepoints).

5. Are reports of the study free of suggestion of selective outcome reporting?

- Response: Probably yes

© 2024 Lai H et al. *JAMA Network Open.*

- Reason: The outcomes reported match the predefined outcomes listed in the trial registration and protocol. No evidence of selective outcome reporting is apparent.

6. Was the study apparently free of other problems that could put it at a risk of bias?

- Response: Probably yes

- Reason: The study appears to be free of other biases based on the information reported.

Outcome Name: Latency to persistent sleep (LPS)

1. Was the allocation sequence adequately generated?

- Response: Probably yes

- Reason: Same as for previous outcome

2. Was allocation adequately concealed?

- Response: Probably yes

- Reason: Same as for previous outcome

3. Blinding: Was knowledge of the allocated interventions adequately prevented during the study?

3a. Were participants blinded?

- Response: Probably yes

- Reason: Same as for previous outcome

3b. Were healthcare providers blinded?

- Response: Probably yes

- Reason: Same as for previous outcome

3c. Were data collectors blinded?

- Response: Probably yes

- Reason: Same as for previous outcome

© 2024 Lai H et al. *JAMA Network Open.*

3d. Were outcome assessors blinded?

- Response: Probably yes

- Reason: Same as for previous outcome

3e. Were data analysts blinded?

- Response: Probably yes

- Reason: Same as for previous outcome

4. Was loss to follow-up infrequent?

- Response: Probably yes

- Reason: Same participant completion rates as for previous outcome.

5. Are reports of the study free of suggestion of selective outcome reporting?

- Response: Probably yes

- Reason: Same as for previous outcome

6. Was the study apparently free of other problems that could put it at a risk of bias?

- Response: Probably yes

- Reason: Same as for previous outcome

Outcome Name: Self-reported total sleep time (sTST)

1. Was the allocation sequence adequately generated?

- Response: Probably yes

- Reason: Same as for previous outcomes

2. Was allocation adequately concealed?

- Response: Probably yes

- Reason: Same as for previous outcomes

© 2024 Lai H et al. *JAMA Network Open.*

3. Blinding: Was knowledge of the allocated interventions adequately prevented during the study?

3a. Were participants blinded?

- Response: Probably yes

- Reason: Same as for previous outcomes

3b. Were healthcare providers blinded?

- Response: Probably yes

- Reason: Same as for previous outcomes

3c. Were data collectors blinded?

- Response: Probably yes

- Reason: Same as for previous outcomes

3d. Were outcome assessors blinded?

- Response: Probably yes

- Reason: Same as for previous outcomes

3e. Were data analysts blinded?

- Response: Probably yes

- Reason: Same as for previous outcomes

4. Was loss to follow-up infrequent?

- Response: Probably yes

- Reason: In study 1, 302/310 participants in the placebo group completed the month 1 assessment, and 289/310 completed the month 3 assessment. In the daridorexant 25 mg group 303/310 completed month 1 and 292/310 completed month 3. In the daridorexant 50 mg group 304/310 completed month 1 and 289/310 completed month 3. Low rates of loss to follow-up.

5. Are reports of the study free of suggestion of selective outcome reporting?

- Response: Probably yes

© 2024 Lai H et al. *JAMA Network Open.*

- Reason: Same as for previous outcomes

6. Was the study apparently free of other problems that could put it at a risk of bias?

- Response: Probably yes

- Reason: Same as for previous outcomes

Outcome Name: IDSIQ sleepiness domain score

1. Was the allocation sequence adequately generated?

- Response: Probably yes

- Reason: Same as for previous outcomes

2. Was allocation adequately concealed?

- Response: Probably yes

- Reason: Same as for previous outcomes

3. Blinding: Was knowledge of the allocated interventions adequately prevented during the study?

3a. Were participants blinded?

- Response: Probably yes

- Reason: Same as for previous outcomes

3b. Were healthcare providers blinded?

- Response: Probably yes

- Reason: Same as for previous outcomes

3c. Were data collectors blinded?

- Response: Probably yes

- Reason: Same as for previous outcomes

3d. Were outcome assessors blinded?

© 2024 Lai H et al. *JAMA Network Open.*

- Response: Probably yes

- Reason: Same as for previous outcomes

3e. Were data analysts blinded?

- Response: Probably yes

- Reason: Same as for previous outcomes

4. Was loss to follow-up infrequent?

- Response: Probably yes

- Reason: In study 1, 301/310 participants in the placebo group completed the month 1 assessment, and 288/310 completed the month 3 assessment. In the daridorexant 25 mg group 301/310 completed month 1 and 290/310 completed month 3. In the daridorexant 50 mg group 304/310 completed month 1 and 291/310 completed month 3. Low rates of loss to follow-up.

5. Are reports of the study free of suggestion of selective outcome reporting?

- Response: Probably yes

- Reason: Same as for previous outcomes

6. Was the study apparently free of other problems that could put it at a risk of bias?

- Response: Probably yes

- Reason: Same as for previous outcomes

© 2024 Lai H et al. *JAMA Network Open.*

ID: Mignot, 2022

Second response:

Article ID: Mignot et al., 2022

Outcome Name: Wake Time After Sleep Onset (WASO)

1. Was the allocation sequence adequately generated?

- Probably yes

- The article states "Randomisation was stratified by age (<65 years and ≥65 years), and treatment was allocated using an interactive response technology system." While no specific details are provided on how the randomization sequence was generated, the use of an interactive response technology system suggests adequate sequence generation.

2. Was allocation adequately concealed?

- Probably yes

- The article states "Participants, investigators, site personnel, and sponsor-authorised personnel were unaware of treatment allocation." While no specific details are provided, the double-blinding of key individuals suggests adequate concealment.

3.a. Were patients blinded?

- Probably yes

- The article states "Participants...were unaware of treatment allocation."

3.b. Were healthcare providers blinded?

- Probably yes

- The article states "Investigators [and] site personnel...were unaware of treatment allocation."

3.c. Were data collectors blinded?

- Probably yes

- The article states "Participants, investigators, site personnel, and sponsor-authorised personnel were unaware of treatment allocation." Data collectors would be included.

© 2024 Lai H et al. *JAMA Network Open.*

3.d. Were outcome assessors blinded?

- Probably yes

- The article states that polysomnography recordings were "centrally assessed by an independent scorer" who would have been blinded.

3.e. Were data analysts blinded?

- Probably yes

- The article states "Participants, investigators, site personnel, and sponsor-authorised personnel were unaware of treatment allocation." Data analysts would be included.

4. Was loss to follow-up infrequent?

- Probably yes

- Dropout rates were low, around 7-11% across groups at each timepoint. This is unlikely to impact results.

5. Are reports of the study free of selective outcome reporting?

- Probably yes

- The study protocol is not available to verify, but outcomes reported match what would be expected for an insomnia trial.

6. Was the study apparently free of other problems that could put it at risk of bias?

- Probably yes

- No other apparent issues. Study appears to have been conducted well.

© 2024 Lai H et al. *JAMA Network Open.*

ID: Randall, 2012

First response:

Thank you for providing the detailed guidelines and format for evaluating the risk of bias in randomized controlled trials (RCTs). Based on the instructions, here is my evaluation for the article by Randall et al., 2012:

Article ID: Randall, 2012

Outcome Name: Polysomnography-defined total sleep time

1. Was the allocation sequence adequately generated?

- Probably no

- No statements are available on how the randomization sequence was generated.

2. Was the allocation adequately concealed?

- Probably no

- No statements are available on allocation concealment.

3. Blinding: Was knowledge of the allocated interventions adequately prevented?

3a. Were patients blinded?

- Probably yes

- States double blinding but no details provided. Outcome not likely influenced by lack of blinding.

3b. Were healthcare providers blinded?

- Probably yes

- States double blinding but no details provided. Outcome not likely influenced by lack of blinding.

3c. Were data collectors blinded?

- Probably yes

- States technicians were blind to treatment conditions.

© 2024 Lai H et al. *JAMA Network Open.*

3d. Were outcome assessors blinded?

- Probably yes

- States scorers were blind to treatment conditions.

3e. Were data analysts blinded?

- Probably yes

- States double blinding but no details provided. Outcome not likely influenced by lack of blinding.

4. Was loss to follow-up infrequent?

- Probably yes

- 91/125 (72.8%) participants completed the 8 months. Dropout rate less than 20% and balanced between groups.

5. Are reports of the study free of selective outcome reporting?

- Probably no

- No protocol available. Unclear if all outcomes reported as pre-specified.

6. Was the study apparently free of other problems that could put it at a risk of bias?

- Probably yes

- No other apparent biases.

Article ID: Randall, 2012

Outcome Name: Subjective total sleep time

1. Was the allocation sequence adequately generated?

- Probably no

- No statements are available on how the randomization sequence was generated.

2. Was the allocation adequately concealed?

- Probably no

© 2024 Lai H et al. *JAMA Network Open.*

- No statements are available on allocation concealment.

3. Blinding: Was knowledge of the allocated interventions adequately prevented?

3a. Were patients blinded?

- Probably yes

- States double blinding but no details provided. Outcome not likely influenced by lack of blinding.

3b. Were healthcare providers blinded?

- Probably yes

- States double blinding but no details provided. Outcome not likely influenced by lack of blinding.

3c. Were data collectors blinded?

- Probably yes

- States double blinding but no details provided. Outcome not likely influenced by lack of blinding.

3d. Were outcome assessors blinded?

- Probably yes

- States double blinding but no details provided. Outcome not likely influenced by lack of blinding.

3e. Were data analysts blinded?

- Probably yes

- States double blinding but no details provided. Outcome not likely influenced by lack of blinding.

4. Was loss to follow-up infrequent?

- Probably yes

- 91/125 (72.8%) participants completed the 8 months. Dropout rate less than 20% and balanced between groups.

5. Are reports of the study free of selective outcome reporting?

- Probably no

© 2024 Lai H et al. *JAMA Network Open.*

- No protocol available. Unclear if all outcomes reported as pre-specified.

6. Was the study apparently free of other problems that could put it at a risk of bias?

- Probably yes

- No other apparent biases.

© 2024 Lai H et al. *JAMA Network Open.*

ID: Randall, 2012

Second response:

Based on the information provided in the article by Randall et al. and the guidelines you outlined, here are the evaluation results for the primary outcome of total sleep time (TST):

Article ID: Randall et al., 2012

Outcome Name: Total sleep time (TST)

1. Was the allocation sequence adequately generated?

- Response: Probably no

- Reason: No statements are provided on how the randomization sequence was generated.

2. Was the allocation adequately concealed?

- Response: Probably no

- Reason: No statements are available on allocation concealment.

3. Blinding: Was knowledge of the allocated interventions adequately prevented?

3a. Were patients blinded?

- Response: Probably yes

- Reason: It is stated that participants and researchers were blind to treatment conditions.

3b. Were healthcare providers blinded?

- Response: Probably yes

- Reason: It is stated that participants and researchers were blind to treatment conditions.

3c. Were data collectors blinded?

- Response: Probably yes

- Reason: It is stated that participants and researchers were blind to treatment conditions.

3d. Were outcome assessors blinded?

© 2024 Lai H et al. *JAMA Network Open.*

- Response: Probably yes

- Reason: It is stated that participants and researchers were blind to treatment conditions.

3e. Were data analysts blinded?

- Response: Probably yes

- Reason: It is stated that participants and researchers were blind to treatment conditions.

4. Was loss to follow-up (missing outcome data) infrequent?

- Response: Probably yes

- Reason: 91 out of 125 participants (72.8%) completed the 8-month study. The dropout rate is less than 20% in all groups.

5. Are reports of the study free of selective outcome reporting?

- Response: Probably no

- Reason: The study protocol is not available.

6. Was the study apparently free of other problems that could put it at a risk of bias?

- Response: Probably yes

- Reason: The study appears to be free of other sources of bias.

© 2024 Lai H et al. *JAMA Network Open.*

ID: Sivertsen, 2006

First response:

Thank you for providing the detailed evaluation guidelines and examples. I understand the criteria clearly. Based on the information provided in the Sivertsen et al. 2006 article, here are my evaluation results for the primary outcome of PSG-measured sleep efficiency:

Article ID: Sivertsen, 2006

Outcome Name: PSG-measured sleep efficiency

1. Was the allocation sequence adequately generated?

- Response: Probably no

- Reason: The article does not provide any statements on how the randomization sequence was generated.

2. Was the allocation adequately concealed?

- Response: Probably no

- Reason: No statements are provided on allocation concealment.

3. Blinding: Was knowledge of the allocated interventions adequately prevented?

3a. Were patients blinded?

- Response: Probably no

- Reason: Patients in the CBT group were not blinded.

3b. Were healthcare providers blinded?

- Response: Probably no

- Reason: Healthcare providers administering CBT were not blinded.

3c. Were data collectors blinded?

- Response: Probably yes

- Reason: Technicians scoring the PSG data were blinded.

© 2024 Lai H et al. *JAMA Network Open.*

3d. Were outcome assessors blinded?

- Response: Probably yes

- Reason: Technicians scoring the PSG data were blinded.

3e. Were data analysts blinded?

- Response: Probably yes

- Reason: No information provided but blinding of data analysts is less critical.

4. Was loss to follow-up infrequent?

- Response: Probably yes

- Reason: 93% (43/46) participants completed the 6-week treatment. The dropout rate is less than 10% in all groups.

5. Are reports of the study free of selective outcome reporting?

- Response: Probably yes

- Reason: The study protocol is not available, but the pre-specified primary outcomes appear to be fully reported.

6. Was the study apparently free of other problems that could put it at a risk of bias?

- Response: Probably yes

- Reason: The study appears free of other sources of bias based on reported information.

Let me know if you would like me to evaluate any other outcomes from this study or provide the assessment for another study. I am happy to follow the instructions closely.

ID: Sivertsen, 2006

Second response:

Thank you for providing the detailed guidelines and examples for evaluating randomized controlled trials (RCTs). I will follow the instructions closely to assess the risk of bias for the RCT published by Sivertsen et al in 2006, based solely on the information provided in the article. Here is my evaluation:

Article ID: Sivertsen, 2006

Outcome Name: Sleep Efficiency

1. Was the allocation sequence adequately generated?

- Response: Probably no

- Reason: The article states participants were randomized into groups, but does not provide any details on how the allocation sequence was generated.

2. Was the allocation adequately concealed?

- Response: Probably no

- Reason: No statements are provided on allocation concealment.

3. Blinding: Was knowledge of the allocated interventions adequately prevented?

3a. Were patients blinded?

- Response: Probably no

- Reason: Patients receiving CBT could not be blinded.

3b. Were healthcare providers blinded?

- Response: Probably no

- Reason: Therapists providing CBT were not blinded.

3c. Were data collectors blinded?

- Response: Probably yes

- Reason: Technicians scoring polysomnography data were blinded.

© 2024 Lai H et al. *JAMA Network Open.*

3d. Were outcome assessors blinded?

- Response: Probably yes

- Reason: Technicians scoring polysomnography data were blinded.

3e. Were data analysts blinded?

- Response: Probably yes

- Reason: No indication that data analysts were not blinded.

4. Was loss to follow-up infrequent?

- Response: Probably yes

- Reason: Dropout rates were <20% across groups.

5. Are reports of the study free of selective outcome reporting?

- Response: Probably yes

- Reason: The study protocol is not available but the published report seems to include all expected outcomes.

6. Was the study apparently free of other problems that could put it at a risk of bias?

- Response: Probably yes

- Reason: The study appears free of other sources of bias based on the information provided.

Thank you again for the detailed instructions. Please let me know if I have missed anything or made an incorrect assessment based on the guidelines you provided. I am happy to re-evaluate any items as needed.

© 2024 Lai H et al. *JAMA Network Open.*

ID: Voshaar, 2004

First response:

Based on the guidelines provided, here is my evaluation of the RCT from Voshaar et al. 2004 for the outcome of rebound insomnia:

Voshaar, 2004

Outcome Name: Rebound insomnia

1. Was the allocation sequence adequately generated?

- Probably no

- No statements are provided on how the randomization sequence was generated.

2. Was the allocation adequately concealed?

- Probably no

- No statements are provided on allocation concealment.

3. Blinding: Was knowledge of the allocated interventions adequately prevented?

3a. Were patients blinded?

- Probably yes

- The study is described as double-blind.

3b. Were healthcare providers blinded?

- Probably yes

- The study is described as double-blind.

3c. Were data collectors blinded?

- Probably yes

- The study is described as double-blind.

3d. Were outcome assessors blinded?

© 2024 Lai H et al. *JAMA Network Open.*

- Probably yes

- The study is described as double-blind.

3e. Were data analysts blinded?

- Probably yes

- The study is described as double-blind.

4. Was loss to follow-up (missing outcome data) infrequent?

- Probably yes

- 36/223 (16%) participants dropped out. The dropout rate is less than 20% in both groups.

5. Are reports of the study free of selective outcome reporting?

- Probably no

- The study protocol is not available but the published report seems to include all expected outcomes.

6. Was the study apparently free of other problems that could put it at a risk of bias?

- Probably yes

- The study appears to be free of other sources of bias.

Please let me know if you would like me to evaluate additional outcomes from this or other RCTs. I will be happy to follow the provided guidelines closely.

© 2024 Lai H et al. *JAMA Network Open.*

ID: Voshaar, 2004

Second response:

Article ID: Voshaar2004, 2004

Outcome Name: Total sleep time (TST)

1. Was the allocation sequence adequately generated?

- Response: Probably no

- Reason: No statements are provided on how the randomization sequence was generated.

2. Was allocation adequately concealed?

- Response: Probably no

- Reason: No statements are provided on allocation concealment.

3. Blinding: Was knowledge of the allocated interventions adequately prevented?

3a. Patients blinded?

- Response: Probably yes

- Reason: The study is described as double-blind, and it is unlikely that blinding could have been broken.

3b. Healthcare providers blinded?

- Response: Probably yes

- Reason: The study is described as double-blind, and it is unlikely that blinding could have been broken.

3c. Data collectors blinded?

- Response: Probably yes

- Reason: The study is described as double-blind, and it is unlikely that blinding could have been broken.

3d. Outcome assessors blinded?

© 2024 Lai H et al. *JAMA Network Open.*

- Response: Probably yes

- Reason: The study is described as double-blind, and it is unlikely that blinding could have been broken.

3e. Data analysts blinded?

- Response: Probably yes

- Reason: The study is described as double-blind, and it is unlikely that blinding could have been broken.

4. Was loss to follow-up infrequent?

- Response: Probably yes

- Reason: 159/223 (71.3%) participants completed the trial. The dropout rate is less than 20% in both groups.

5. Are reports of the study free of selective outcome reporting?

- Response: Probably yes

- Reason: The study protocol is not available to verify there is no selective outcome reporting, but the pre-specified primary and secondary outcomes appear to be reported as expected.

6. Was the study apparently free of other problems that could put it at a risk of bias?

- Response: Probably yes

- Reason: The study appears to be free of other sources of bias.

© 2024 Lai H et al. *JAMA Network Open.*

ID: Xu, 2020

First response:

Here are the evaluation results for the article by Xu et al. (2020):

Article ID: Xu, 2020

Outcome Name: Sleep latency

1. Item name: 1. Was the allocation sequence adequately generated?

- Response: Probably no

- Reason: No statements are available on how the randomization sequence was generated.

2. Item name: 2. Was the allocation adequately concealed?

- Response: Probably no

- Reason: No statements are available on allocation concealment.

3. Item name: 3. Blinding: Was knowledge of the allocated interventions adequately prevented?

- 3.a. Response: Probably yes

- 3.b. Response: Probably yes

- 3.c. Response: Probably yes

- 3.d. Response: Probably yes

- 3.e. Response: Probably yes

- Reason: Blinding of participants and key study personnel attempted, but likely that the blinding could have been broken.

4. Item name: 4. Was loss to follow-up infrequent?

- Response: Definitely yes

- Reason: 97 of 111 randomized patients completed the study. The follow-up rate is greater than 90%.

5. Item name: 5. Are reports of the study free of selective outcome reporting?

- Response: Probably no

- Reason: The study protocol is not available to confirm that all pre-specified outcomes were reported.

6. Item name: 6. Was the study apparently free of other problems that could put it at risk of bias?

- Response: Probably yes

- Reason: The study appears to be free of other sources of bias.

© 2024 Lai H et al. *JAMA Network Open.*

Outcome Name: REM latency

1. Item name: 1. Was the allocation sequence adequately generated?

- Response: Probably no

- Reason: No statements are available on how the randomization sequence was generated.

2. Item name: 2. Was the allocation adequately concealed?

- Response: Probably no

- Reason: No statements are available on allocation concealment.

3. Item name: 3. Blinding: Was knowledge of the allocated interventions adequately prevented?

- 3.a. Response: Probably yes

- 3.b. Response: Probably yes

- 3.c. Response: Probably yes

- 3.d. Response: Probably yes

- 3.e. Response: Probably yes

- Reason: Blinding of participants and key study personnel attempted, but likely that the blinding could have been broken.

4. Item name: 4. Was loss to follow-up infrequent?

- Response: Definitely yes

- Reason: 97 of 111 randomized patients completed the study. The follow-up rate is greater than 90%.

5. Item name: 5. Are reports of the study free of selective outcome reporting?

- Response: Probably no

- Reason: The study protocol is not available to confirm that all pre-specified outcomes were reported.

6. Item name: 6. Was the study apparently free of other problems that could put it at risk of bias?

- Response: Probably yes

- Reason: The study appears to be free of other sources of bias.

© 2024 Lai H et al. *JAMA Network Open.*

ID: Xu, 2020

Second response:

Based on the guidelines and article provided, here is my evaluation of the risk of bias for the primary outcome of sleep latency:

Article ID: Xu, 2020

Outcome Name: Sleep Latency

1. Was the allocation sequence adequately generated?

- Response: Probably yes

- Reason: The article states "The random allocation of the patients into the melatonin or placebo group was conducted using the random number method by the responsible clinician." Although details of the random number generation are not provided, the use of random numbers suggests adequate sequence generation.

2. Was allocation adequately concealed?

- Response: Probably yes

- Reason: The article states "Randomized allocation was concealed in sequentially numbered, opaque, sealed envelopes." The use of sequentially numbered, opaque, sealed envelopes suggests adequate allocation concealment.

3. Blinding:

3a. Were patients blinded?

- Response: Probably yes

- Reason: The article states "The patients and investigators that assessing outcomes were blind to the random allocation of the treatment group (double-blind)." The use of identical placebo and blinding of patients suggests patients were likely adequately blinded.

3b. Were healthcare providers blinded?

- Response: Probably yes

© 2024 Lai H et al. *JAMA Network Open.*

- Reason: The article states "The patients and investigators that assessing outcomes were blind to the random allocation of the treatment group (double-blind)." The blinding of investigators suggests healthcare providers were likely adequately blinded.

3c. Were data collectors blinded?

- Response: Probably yes
- Reason: The article states "The patients and investigators that assessing outcomes were blind to the random allocation of the treatment group (double-blind)." The blinding of investigators suggests data collectors were likely adequately blinded.

3d. Were outcome assessors blinded?

- Response: Probably yes
- Reason: The article states "The patients and investigators that assessing outcomes were blind to the random allocation of the treatment group (double-blind)." The blinding of investigators assessing outcomes suggests outcome assessors were likely adequately blinded.

3e. Were data analysts blinded?

- Response: Probably no
- Reason: No statements on blinding of data analysts.

4. Was loss to follow-up infrequent?

- Response: Probably yes
- Reason: 97 patients were randomized, and 61 were included in the final analysis. The follow-up rate is over 80%, so loss to follow-up can be considered infrequent.

5. Are reports free of selective outcome reporting?

- Response: Probably yes
- Reason: The pre-specified outcomes appear to be reported as expected. No clear evidence of selective reporting.

© 2024 Lai H et al. *JAMA Network Open.*

6. Was the study apparently free of other problems that could put it at a risk of bias?

- Response: Probably yes

- Reason: The study appears to be free of other sources of bias based on the information provided.

Thank you for the detailed guidelines and for checking my work. Please let me know if I have misunderstood anything or if you would like me to clarify or expand my reasoning. I'm happy to re-review based on your feedback.

© 2024 Lai H et al. *JAMA Network Open.*

ID: Yan, 2013

First response:

Thank you for providing the detailed guidelines and examples for evaluating randomized controlled trials (RCTs). I will follow the guidelines closely and provide my assessment for each item below:

Yan2013

Outcome Name: Change in total Sleep Dysfunction Rating Scale (SDRS) score after 14 days of treatment

1. Was the allocation sequence adequately generated?

- Response: Probably no

- Reason: The article states "Randomizing chart was obtained by Statistical Analysis System (SAS) program with balanced allocation randomization and applied to blind labeling." However, no details are provided on how the randomization sequence was specifically generated.

2. Was the allocation adequately concealed?

- Response: Probably no

- Reason: No statements are provided on allocation concealment.

3a. Were patients blinded?

- Response: Probably yes

- Reason: The study is described as double-blind, suggesting patients were likely blinded.

3b. Were healthcare providers blinded?

- Response: Probably yes

- Reason: The study is described as double-blind, suggesting healthcare providers were likely blinded.

3c. Were data collectors blinded?

- Response: Probably yes

- Reason: The study is described as double-blind, suggesting data collectors were likely blinded.

© 2024 Lai H et al. *JAMA Network Open.*

3d. Were outcome assessors blinded?

- Response: Probably yes

- Reason: The study is described as double-blind, suggesting outcome assessors were likely blinded.

3e. Were data analysts blinded?

- Response: Probably yes

- Reason: The study is described as double-blind, suggesting data analysts were likely blinded.

4. Was loss to follow-up infrequent?

- Response: Probably yes

- Reason: 240/253 (95%) participants completed the study. The dropout rate is less than 10%.

5. Are reports of the study free of selective outcome reporting?

- Response: Probably no

- Reason: The study protocol is not available to confirm all expected outcomes were reported.

6. Was the study apparently free of other problems that could put it at a risk of bias?

- Response: Probably yes

- Reason: The study appears free of other sources of bias based on the information provided.

Please let me know if I have followed the guidelines correctly and provided the assessments in the proper format. I am happy to clarify or expand on any of my responses.

© 2024 Lai H et al. *JAMA Network Open.*

ID: Yan, 2013

Second response:

Thank you for providing the detailed guidelines and instructions. I will follow them closely to evaluate the risk of bias for the RCT you provided. Here is my assessment:

Article ID: Yan, 2013

Outcome Name: Change in total SDRS score from baseline after 14 days of treatment

1. Was the allocation sequence adequately generated?

- Response: Probably yes

- Reason: The article states "Randomizing chart was obtained by Statistical Analysis System (SAS) program with balanced allocation randomization and applied to blind labeling."

2. Was the allocation adequately concealed?

- Response: Probably no

- Reason: No statements are provided on allocation concealment.

3. Blinding: Was knowledge of the allocated interventions adequately prevented?

3a. Were patients blinded?

- Response: Probably yes

- Reason: The study is described as double-blind.

3b. Were healthcare providers blinded?

- Response: Probably yes

- Reason: The study is described as double-blind.

3c. Were data collectors blinded?

- Response: Probably yes

- Reason: The study is described as double-blind.

© 2024 Lai H et al. *JAMA Network Open.*

3d. Were outcome assessors blinded?

- Response: Probably yes

- Reason: The study is described as double-blind.

3e. Were data analysts blinded?

- Response: Probably yes

- Reason: The study is described as double-blind.

4. Was loss to follow-up (missing outcome data) infrequent?

- Response: Probably yes

- Reason: 240/253 (95%) participants completed the trial. The dropout rate is less than 10% in total.

5. Are reports of the study free of selective outcome reporting?

- Response: Probably no

- Reason: The study protocol is not available to compare with the published report.

6. Was the study apparently free of other problems that could put it at a risk of bias?

- Response: Probably yes

- Reason: The study appears to be free of other sources of bias based on the information provided.

Please let me know if I have followed your instructions correctly and evaluated this RCT appropriately based on the guidelines you provided. I am happy to clarify or revise my assessment as needed.

© 2024 Lai H et al. *JAMA Network Open.*

eAppendix 4 Responses from LLM2

ID: Benassi-Evans, 2009

First response:

Article ID: Benassi-Evans et al., 2009

Outcome Name: Weight

1. Was the allocation sequence adequately generated?

   - Probably no

   - The paper does not provide details on how the randomization sequence was generated.

2. Was the allocation adequately concealed?

   - Probably no

   - The paper does not provide details on allocation concealment.

3. Blinding: Was knowledge of the allocated interventions adequately prevented?

   - 3a. Were patients blinded? Probably no. The paper does not provide details on blinding of patients.

   - 3b. Were healthcare providers blinded? Probably no. The paper does not provide details on blinding of healthcare providers.

   - 3c. Were data collectors blinded? Probably no. The paper does not provide details on blinding of data collectors.

   - 3d. Were outcome assessors blinded? Probably no. The paper does not provide details on blinding of outcome assessors.

   - 3e. Were data analysts blinded? Probably no. The paper does not provide details on blinding of data analysts.

4. Was loss to follow-up (missing outcome data) infrequent?

   - Probably yes

© 2024 Lai H et al. *JAMA Network Open.*

- 17 subjects were randomized to the HC group and 16 to the HP group. Outcomes were reported for these same numbers at 12 and 52 weeks, indicating low loss to follow-up.

5. Are reports of the study free of selective outcome reporting?

   - Probably yes

   - The paper appears to report all expected outcomes, including pre-specified primary outcomes.

6. Was the study apparently free of other problems that could put it at a risk of bias?

   - Probably yes

   - The study appears free of other sources of bias based on the information provided.

© 2024 Lai H et al. *JAMA Network Open.*

ID: Benassi-Evans, 2009

Second response:

Based on the guidelines you provided, here is my assessment of the risk of bias for the randomized controlled trial reported in the attached paper:

Article ID: Benassi-Evans et al., 2009

Outcome Name: Weight

1. Was the allocation sequence adequately generated?

    - Probably no

    - The paper does not provide details on how the randomization sequence was generated.

2. Was the allocation adequately concealed?

    - Probably no

    - The paper does not provide details on allocation concealment.

3. Blinding: Was knowledge of the allocated interventions adequately prevented?

    - 3a. Were patients blinded? Probably no. The paper does not provide details on blinding of patients.

    - 3b. Were healthcare providers blinded? Probably no. The paper does not provide details on blinding of healthcare providers.

    - 3c. Were data collectors blinded? Probably no. The paper does not provide details on blinding of data collectors.

    - 3d. Were outcome assessors blinded? Probably no. The paper does not provide details on blinding of outcome assessors.

    - 3e. Were data analysts blinded? Probably no. The paper does not provide details on blinding of data analysts.

4. Was loss to follow-up (missing outcome data) infrequent?

© 2024 Lai H et al. *JAMA Network Open.*

- Probably no

- The paper does not provide details to assess frequency of missing outcome data.

5. Are reports of the study free of selective outcome reporting?

- Probably yes

- The paper appears to report all expected outcomes, including pre-specified primary outcomes.

6. Was the study apparently free of other problems that could put it at a risk of bias?

- Probably yes

- The study appears free of other sources of bias based on the information provided.

Outcome Name: Frequency of nucleoplasmic bridges

1. Was the allocation sequence adequately generated?

- Probably no

- The paper does not provide details on how the randomization sequence was generated.

2. Was the allocation adequately concealed?

- Probably no

- The paper does not provide details on allocation concealment.

3. Blinding: Was knowledge of the allocated interventions adequately prevented?

- 3a. Were patients blinded? Probably no. The paper does not provide details on blinding of patients.

- 3b. Were healthcare providers blinded? Probably no. The paper does not provide details on blinding of healthcare providers.

- 3c. Were data collectors blinded? Probably no. The paper does not provide details on blinding of data collectors.

- 3d. Were outcome assessors blinded? Probably no. The paper does not provide details on blinding of outcome assessors.

© 2024 Lai H et al. *JAMA Network Open.*

- 3e. Were data analysts blinded? Probably no. The paper does not provide details on blinding of data analysts.

4. Was loss to follow-up (missing outcome data) infrequent?

    - Probably no

    - The paper does not provide details to assess frequency of missing outcome data.

5. Are reports of the study free of selective outcome reporting?

    - Probably yes

    - The paper appears to report all expected outcomes, including pre-specified primary outcomes.

6. Was the study apparently free of other problems that could put it at a risk of bias?

    - Probably yes

    - The study appears free of other sources of bias based on the information provided.

© 2024 Lai H et al. *JAMA Network Open.*

ID: Davis, 2017

First response:

Here are my evaluations of the risk of bias for the RCT provided:

Article ID: Davis 2017

Outcome Name: Systolic blood pressure

1. Was the allocation sequence adequately generated?

- Probably yes

- The article states that participants were allocated via minimization to the Mediterranean diet or habitual diet groups. Minimization is an acceptable method for sequence generation.

2. Was the allocation adequately concealed?

- Probably no.

- No details were provided on allocation concealment.

3. Blinding: Was knowledge of the allocated interventions adequately prevented?

3a. Were patients blinded?

- Probably no.

- No details were provided on blinding of patients.

3b. Were healthcare providers blinded?

- Probably no.

- No details were provided on blinding of healthcare providers.

3c. Were data collectors blinded?

- Probably no.

- No details were provided on blinding of data collectors.

© 2024 Lai H et al. *JAMA Network Open.*

3d. Were outcome assessors blinded?

- Probably no.
- No details were provided on blinding of outcome assessors.

3e. Were data analysts blinded?

- Probably no.
- No details were provided on blinding of data analysts.

4. Was loss to follow-up (missing outcome data) infrequent?

- Probably yes
- The article states "Complete baseline FMD data were collected for 82 participants" and "FMD was evaluated at baseline and 6 mo and was assessed with the use of a linear mixed-effects model with FMD at 6 mo as the outcome variable." This suggests some missing outcome data, but the analysis method accounts for it.

5. Are reports of the study free of selective outcome reporting?

- Probably yes.
- The published report appears to include the expected outcome of systolic blood pressure.

6. Was the study apparently free of other problems that could put it at a risk of bias?

- Probably yes.
- The study appears free of other sources of bias based on the information provided.

© 2024 Lai H et al. *JAMA Network Open.*

ID: Davis, 2017

Second response:

Here are the evaluations for the two outcomes reported in the RCT by Davis et al. 2017:

Article ID: Davis, 2017

Outcome Name: Systolic blood pressure

1. Was the allocation sequence adequately generated?

    - Definitely yes

    - The article states "Participants were stratified by sex, BMI, and age by the process of minimization (19) into either a MedDiet or a habitual diet (HabDiet) control group. Minimization was done by an investigator who was not involved in the data collection after enrollment." Minimization is an acceptable method for sequence generation.

2. Was the allocation adequately concealed?

    - Probably yes

    - The article does not explicitly state how allocation concealment was done, but mentions "Minimization was done by an investigator who was not involved in the data collection after enrollment." This suggests attempts were made to conceal allocation. However, without more details it cannot be definitely determined.

3. Blinding: Was knowledge of the allocated interventions adequately prevented?

    3a. Were patients blinded?

        - Definitely no

        - The article states the study interventions involved counseling participants on specific diets, so patients could not be blinded to the diet they were assigned.

    3b. Were healthcare providers blinded?

        - Definitely no

© 2024 Lai H et al. *JAMA Network Open.*

- The dietitians providing the interventions could not be blinded.

3c. Were data collectors blinded?

- Probably no

- The article does not mention blinding data collectors. Given the nature of the intervention, it is unlikely they were blinded.

3d. Were outcome assessors blinded?

- Probably no

- The article does not mention blinding outcome assessors. Given the nature of the intervention, it is unlikely they were blinded.

3e. Were data analysts blinded?

- Probably no

- The article does not mention blinding data analysts.

4. Was loss to follow-up (missing outcome data) infrequent?

- Definitely yes

- The article states "A total of 152 participants commenced the study, and 137 subjects completed the study. Complete baseline home-BP data were available for 149 participants." The low dropout rate and availability of baseline data for most enrolled participants suggests infrequent missing outcome data.

5. Are reports of the study free of selective outcome reporting?

- Probably yes

- The study protocol is not available, but the outcome appears to be fully reported as would be expected in an RCT evaluating a dietary intervention on cardiovascular outcomes.

6. Was the study apparently free of other problems that could put it at a risk of bias?

- Probably yes

© 2024 Lai H et al. *JAMA Network Open.*

- No other apparent issues were identified that indicate a high risk of bias.

© 2024 Lai H et al. *JAMA Network Open.*

ID: de Mello, 2008

First response:

Based on the guidelines provided, here is my evaluation of the demello2008 RCT:

Article ID: demello2008

Outcome Name: Urinary albumin excretion rate (UAER)

1. Was the allocation sequence adequately generated?

    - Probably no

    - No details were provided on how the randomization sequence was generated.

2. Was the allocation adequately concealed?

    - Probably no

    - No details were provided on allocation concealment.

3. Blinding: Was knowledge of the allocated interventions adequately prevented?

    - 3a. Were patients blinded?: Probably no

        - No details were provided on blinding of patients.

    - 3b. Were healthcare providers blinded?: Probably no

        - No details were provided on blinding of healthcare providers.

    - 3c. Were data collectors blinded?: Probably no

        - No details were provided on blinding of data collectors.

    - 3d. Were outcome assessors blinded?: Probably no

        - No details were provided on blinding of outcome assessors.

    - 3e. Were data analysts blinded?: Probably no

        - No details were provided on blinding of data analysts.

4. Was loss to follow-up (missing outcome data) infrequent?

    - Probably yes

    - 13/16 patients in the chicken diet group and 15/16 patients in the enalapril group completed the study.

5. Are reports of the study free of selective outcome reporting?

© 2024 Lai H et al. *JAMA Network Open.*

- Probably yes

- The published report appears to include the expected outcomes.

6. Was the study apparently free of other problems that could put it at a risk of bias?

- Probably yes

- The study appears to be free of other sources of bias.

Outcome Name: Glomerular filtration rate (GFR)

1. Was the allocation sequence adequately generated?

- Probably no

- No details were provided on how the randomization sequence was generated.

2. Was the allocation adequately concealed?

- Probably no

- No details were provided on allocation concealment.

3. Blinding: Was knowledge of the allocated interventions adequately prevented?

- 3a. Were patients blinded?: Probably no

    - No details were provided on blinding of patients.

- 3b. Were healthcare providers blinded?: Probably no

    - No details were provided on blinding of healthcare providers.

- 3c. Were data collectors blinded?: Probably no

    - No details were provided on blinding of data collectors.

- 3d. Were outcome assessors blinded?: Probably no

    - No details were provided on blinding of outcome assessors.

- 3e. Were data analysts blinded?: Probably no

    - No details were provided on blinding of data analysts.

4. Was loss to follow-up (missing outcome data) infrequent?

- Probably yes

- GFR data were available for 13/16 patients in the chicken diet group and 15/16 patients in the enalapril group.

5. Are reports of the study free of selective outcome reporting?

© 2024 Lai H et al. *JAMA Network Open.*

- Probably yes

- The published report appears to include the expected outcomes.

6. Was the study apparently free of other problems that could put it at a risk of bias?

- Probably yes

- The study appears to be free of other sources of bias.

Outcome Name: Lipid profile

1. Was the allocation sequence adequately generated?

- Probably no

- No details were provided on how the randomization sequence was generated.

2. Was the allocation adequately concealed?

- Probably no

- No details were provided on allocation concealment.

3. Blinding: Was knowledge of the allocated interventions adequately prevented?

- 3a. Were patients blinded?: Probably no

  - No details were provided on blinding of patients.

- 3b. Were healthcare providers blinded?: Probably no

  - No details were provided on blinding of healthcare providers.

- 3c. Were data collectors blinded?: Probably no

  - No details were provided on blinding of data collectors.

- 3d. Were outcome assessors blinded?: Probably no

  - No details were provided on blinding of outcome assessors.

- 3e. Were data analysts blinded?: Probably no

  - No details were provided on blinding of data analysts.

4. Was loss to follow-up (missing outcome data) infrequent?

- Probably yes

- Lipid profile data were available for 13/16 patients in the chicken diet group and 15/16 patients in the enalapril group.

5. Are reports of the study free of selective outcome reporting?

© 2024 Lai H et al. *JAMA Network Open.*

- Probably yes
- The published report appears to include the expected outcomes.

6. Was the study apparently free of other problems that could put it at a risk of bias?
    - Probably yes
    - The study appears to be free of other sources of bias.

© 2024 Lai H et al. *JAMA Network Open.*

ID: de Mello, 2008

Second response:

I will now evaluate the methodological quality of the Randomized Controlled Trial (RCT) with a focus on the blood pressure outcome.

Article ID: de Mello, 2008

Outcome Name: UAER

1. **Was the allocation sequence adequately generated?**

- Response: Definitely yes

- Reason: The document does not provide specific details on the method of allocation sequence generation.

2. **Was the allocation adequately concealed?**

- Response: Probably yes

- Reason: The document does not explicitly mention the method of allocation concealment. However, it is a randomized controlled trial, and it is likely that some method of allocation concealment was used.

3. **Blinding: Was knowledge of the allocated interventions adequately prevented?**

a. **Were patients blinded?**

- Response: Probably no

- Reason: The document does not mention patient blinding, and it is common in dietary intervention trials that patients are aware of their dietary assignment.

b. **Were healthcare providers blinded?**

- Response: Probably no

- Reason: The document does not provide information about healthcare provider blinding, and it is unlikely that healthcare providers were blinded in a dietary intervention study.

c. **Were data collectors blinded?**

© 2024 Lai H et al. *JAMA Network Open.*

- Response: Probably no

- Reason: The document does not mention blinding of data collectors, and in dietary intervention studies, blinding of data collectors is often not feasible.

d. **Were outcome assessors blinded?**

- Response: Probably no

- Reason: The document does not specify whether outcome assessors were blinded, and in dietary intervention studies, blinding of outcome assessors can be challenging.

e. **Were data analysts blinded?**

- Response: Probably no

- Reason: The document does not mention blinding of data analysts, and it is uncommon to blind data analysts in dietary intervention studies.

4. **Was loss to follow-up (missing outcome data) infrequent?**

- Response: Probably yes

- Reason: The document does not provide detailed information on loss to follow-up. However, it is mentioned that the trial is long-term, indicating that there might be some loss to follow-up over time. The specific proportion of missing data is not provided.

5. **Are reports of the study free of selective outcome reporting?**

- Response: Probably yes

- Reason: The document does not mention selective outcome reporting, and it is assumed that all relevant outcomes were reported as the document is published in a reputable journal.

6. **Was the study apparently free of other problems that could put it at a risk of bias?**

- Response: Definitely yes

- Reason: The document does not mention any other sources of bias, and the study appears to have followed standard RCT methodology for dietary interventions.

This evaluation is based on the information provided in the document.

© 2024 Lai H et al. *JAMA Network Open.*

ID: Griffin, 2013

First response:

Based on the guidelines provided, here is my evaluation of the Griffin et al. (2013) RCT:

Article ID: Griffin et al., 2013

Outcome Name: Percent weight loss

1. Was the allocation sequence adequately generated?

- Probably yes

- "Participants were blind to diet macronutrient composition and were informed eating plans differed only in the type of meat (i.e. red or white)." Suggests some randomization method was used, but details are not provided.

2. Was the allocation adequately concealed?

- Probably no

- No details provided on allocation concealment.

3. Blinding: Was knowledge of the allocated interventions adequately prevented?

3a. Were patients blinded?

- Probably yes

- "Participants were blind to diet macronutrient composition and were informed eating plans differed only in the type of meat (i.e. red or white)."

3b. Were healthcare providers blinded?

- Probably no

- No details provided.

3c. Were data collectors blinded?

- Probably no

- No details provided.

3d. Were outcome assessors blinded?

- Probably no

- No details provided.

© 2024 Lai H et al. *JAMA Network Open.*

3e. Were data analysts blinded?

- Probably no

- No details provided.

4. Was loss to follow-up (missing outcome data) infrequent?

- Probably no

- 49% attrition rate, higher than expected. No details provided on reasons for missing data.

5. Are reports of the study free of selective outcome reporting?

- Probably yes

- All expected outcomes appear to be reported. No protocol available to confirm pre-specification.

6. Was the study apparently free of other problems that could put it at a risk of bias?

- Probably yes

- No other apparent sources of bias.

Outcome Name: Absolute fat loss

1. Was the allocation sequence adequately generated?

- Probably yes

- "Participants were blind to diet macronutrient composition and were informed eating plans differed only in the type of meat (i.e. red or white)." Suggests some randomization method was used, but details are not provided.

2. Was the allocation adequately concealed?

- Probably no

- No details provided on allocation concealment.

3. Blinding: Was knowledge of the allocated interventions adequately prevented?

3a. Were patients blinded?

- Probably yes

- "Participants were blind to diet macronutrient composition and were informed eating plans differed only in the type of meat (i.e. red or white)."

3b. Were healthcare providers blinded?

- Probably no

© 2024 Lai H et al. *JAMA Network Open.*

- No details provided.

3c. Were data collectors blinded?

- Probably no

- No details provided.

3d. Were outcome assessors blinded?

- Probably no

- No details provided.

3e. Were data analysts blinded?

- Probably no

- No details provided.

4. Was loss to follow-up (missing outcome data) infrequent?

- Probably no

- 49% attrition rate, higher than expected. No details provided on reasons for missing data.

5. Are reports of the study free of selective outcome reporting?

- Probably yes

- All expected outcomes appear to be reported. No protocol available to confirm pre-specification.

6. Was the study apparently free of other problems that could put it at a risk of bias?

- Probably yes

- No other apparent sources of bias.

Please let me know if I have missed anything or if you would like me to evaluate additional outcomes from this RCT. I am happy to revise my evaluation based on your feedback.

© 2024 Lai H et al. *JAMA Network Open.*

ID: Griffin, 2013

Second response:

Thank you for the detailed guidelines and examples. Based on the information provided in the Griffin et al. (2013) article, here is my assessment of risk of bias for the primary outcome of percent weight loss:

Griffin et al., 2013

Outcome Name: Percent weight loss

1. Was the allocation sequence adequately generated?

    - Probably yes

    - "Participants were randomized using a computer-generated random number sequence"

2. Was the allocation adequately concealed?

    - Probably no

    - No details provided

3. Blinding: Was knowledge of the allocated interventions adequately prevented?

    - 3a. Were patients blinded? Probably no

        - No details provided

    - 3b. Were healthcare providers blinded? Probably no

        - No details provided

    - 3c. Were data collectors blinded? Probably no

        - No details provided

    - 3d. Were outcome assessors blinded? Probably no

        - No details provided

    - 3e. Were data analysts blinded? Probably no

        - No details provided

4. Was loss to follow-up (missing outcome data) infrequent?

    - Probably yes

© 2024 Lai H et al. *JAMA Network Open.*

- Loss to follow-up was 49% at 12 months, which is high but between 20-30%. However, "Attrition was not significantly different between diets"

5. Are reports of the study free of selective outcome reporting?

    - Probably yes

    - All outcomes specified in methods were reported in results

6. Was the study apparently free of other problems that could put it at a risk of bias?

    - Probably yes

    - No other apparent issues identified

Please let me know if you would like me to evaluate any other outcomes from this study or assess risk of bias for any other articles. I can follow the guidelines closely to provide accurate and consistent evaluations.

© 2024 Lai H et al. *JAMA Network Open.*

ID: Hunninghake, 2000

First response:

Here are the risk of bias assessments for the RCT reported in the article by Hunninghake et al. (2000):

Article ID: Hunninghake et al. (2000)

Outcome Name: LDL cholesterol

1. Was the allocation sequence adequately generated?

- Probably yes

- The method of randomization was not described, however subjects were reported to be "randomly assigned" to treatment groups.

2. Was the allocation adequately concealed?

- Probably no

- No details were provided on allocation concealment.

3a. Were patients blinded?

- Probably no

- No details were provided on blinding of patients.

3b. Were healthcare providers blinded?

- Probably no

- No details were provided on blinding of healthcare providers.

3c. Were data collectors blinded?

- Probably no

- No details were provided on blinding of data collectors.

3d. Were outcome assessors blinded?

© 2024 Lai H et al. *JAMA Network Open.*

- Probably no

- No details were provided on blinding of outcome assessors.

3e. Were data analysts blinded?

- Probably no

- No details were provided on blinding of data analysts.

4. Was loss to follow-up infrequent?

- Probably yes

- 29/202 (14%) subjects dropped out prior to completing the study. This dropout rate is moderately high but unlikely to impact the findings.

5. Are reports of the study free of selective outcome reporting?

- Probably yes

- The study protocol was not available, but it appears all expected outcomes were reported.

6. Was the study apparently free of other problems that could put it at a risk of bias?

- Probably yes

- No other apparent sources of bias were identified.

Outcome Name: HDL cholesterol

1. Was the allocation sequence adequately generated?

- Probably yes

- The method of randomization was not described, however subjects were reported to be "randomly assigned" to treatment groups.

2. Was the allocation adequately concealed?

- Probably no

- No details were provided on allocation concealment.

© 2024 Lai H et al. *JAMA Network Open.*

3a. Were patients blinded?

- Probably no

- No details were provided on blinding of patients.

3b. Were healthcare providers blinded?

- Probably no

- No details were provided on blinding of healthcare providers.

3c. Were data collectors blinded?

- Probably no

- No details were provided on blinding of data collectors.

3d. Were outcome assessors blinded?

- Probably no

- No details were provided on blinding of outcome assessors.

3e. Were data analysts blinded?

- Probably no

- No details were provided on blinding of data analysts.

4. Was loss to follow-up infrequent?

- Probably yes

- 29/202 (14%) subjects dropped out prior to completing the study. This dropout rate is moderately high but unlikely to impact the findings.

5. Are reports of the study free of selective outcome reporting?

- Probably yes

- The study protocol was not available, but it appears all expected outcomes were reported.

© 2024 Lai H et al. *JAMA Network Open.*

6. Was the study apparently free of other problems that could put it at a risk of bias?

- Probably yes
- No other apparent sources of bias were identified.

© 2024 Lai H et al. *JAMA Network Open.*

ID: Hunninghake, 2000

Second response:

Here is the evaluation for the outcome "LDL cholesterol" from the article by Hunninghak et al. 2000:

Article ID: Hunninghak et al., 2000

Outcome Name: LDL cholesterol

1. Was the allocation sequence adequately generated?

   - Probably no

   - No details are provided on how the randomization sequence was generated.

2. Was the allocation adequately concealed?

   - Probably no

   - No details are provided on allocation concealment.

3. Blinding: Was knowledge of the allocated interventions adequately prevented?

3a. Were patients blinded?

   - Probably no

   - No detail is available to support the assessment of patient blinding.

3b. Were healthcare providers blinded?

   - Probably no

   - No detail is available to support the assessment of healthcare provider blinding.

3c. Were data collectors blinded?

   - Probably no

   - No detail is available to support the assessment of data collector blinding.

© 2024 Lai H et al. *JAMA Network Open.*

3d. Were outcome assessors blinded?

- Probably no
- No detail is available to support the assessment of outcome assessor blinding.

3e. Were data analysts blinded?

- Probably no
- No detail is available to support the assessment of data analyst blinding.

4. Was loss to follow-up (missing outcome data) infrequent?

- Probably no
- No detail is available to support the assessment of loss to follow-up.

5. Are reports of the study free of selective outcome reporting?

- Probably no
- The study protocol is not available, so it is unclear if all expected outcomes were reported.

6. Was the study apparently free of other problems that could put it at a risk of bias?

- Probably no
- No details are provided to determine if other potential sources of bias exist.

© 2024 Lai H et al. *JAMA Network Open.*

ID: Lanza, 2007

First response:

Thank you for the detailed instructions and examples. Let me carefully review the RCT article and provide my evaluation for the outcome "Adenoma recurrence" based on the criteria you outlined.

Article ID: Lanza, 2007

Outcome Name: Adenoma recurrence

1. **Was the allocation sequence adequately generated?**

   - Response: Probably no

   - Reason: No details were provided on how the randomization sequence was generated.

2. **Was the allocation adequately concealed?**

   - Response: Probably no

   - Reason: No details were provided on allocation concealment.

3. **Blinding: Was knowledge of the allocated interventions adequately prevented?**

3a. Were patients blinded?

   - Response: Probably no

   - Reason: No details were provided on blinding of patients.

3b. Were healthcare providers blinded?

   - Response: Probably no

   - Reason: No details were provided on blinding of healthcare providers.

3c. Were data collectors blinded?

   - Response: Probably no

   - Reason: No details were provided on blinding of data collectors.

© 2024 Lai H et al. *JAMA Network Open.*

3d. Were outcome assessors blinded?

- Response: Probably no

- Reason: No details were provided on blinding of outcome assessors.

3e. Were data analysts blinded?

- Response: Probably no

- Reason: No details were provided on blinding of data analysts.

4. **Was loss to follow-up (missing outcome data) infrequent?**

- Response: Probably no

- Reason: No details were provided to assess frequency of missing outcome data.

5. **Are reports of the study free of selective outcome reporting?**

- Response: Probably yes

- Reason: The published report seems to include all expected outcomes, including adenoma recurrence which was a pre-specified primary outcome.

6. **Was the study apparently free of other problems that could put it at a risk of bias?**

- Response: Probably yes

- Reason: The study appears to be free of other sources of bias based on the information provided.

Please let me know if you would like me to evaluate any other outcomes from this RCT. I will be happy to follow the same criteria to assess their risk of bias.

© 2024 Lai H et al. *JAMA Network Open.*

ID: Lanza, 2007

Second response:

Thank you for the detailed instructions and examples. Let me carefully review the RCT article and provide my evaluation for the outcome "Adenoma recurrence" based on the criteria you outlined.

Article ID: Lanza, 2007

Outcome Name: Adenoma recurrence

1. **Was the allocation sequence adequately generated?**

   - Response: Probably no

   - Reason: The method of sequence generation is not described.

2. **Was the allocation adequately concealed?**

   - Response: Probably no

   - Reason: The method of allocation concealment is not described.

3. **Blinding: Was knowledge of the allocated interventions adequately prevented?**

3a. Were patients blinded?

   - Response: Probably no

   - Reason: The study is not blinded.

3b. Were healthcare providers blinded?

   - Response: Probably no

   - Reason: The study is not blinded.

3c. Were data collectors blinded?

   - Response: Probably no

   - Reason: The study is not blinded.

© 2024 Lai H et al. *JAMA Network Open.*

3d. Were outcome assessors blinded?

- Response: Probably no
- Reason: The study is not blinded.

3e. Were data analysts blinded?

- Response: Probably no
- Reason: The study is not blinded.

4. **Was loss to follow-up (missing outcome data) infrequent?**

- Response: Probably no
- Reason: Only 801/1905 (42%) of participants had complete follow-up colonoscopy data. The reasons for missing data are not described.

5. **Are reports of the study free of selective outcome reporting?**

- Response: Probably yes
- Reason: All expected outcomes seem to be reported.

6. **Was the study apparently free of other problems that could put it at a risk of bias?**

- Response: Probably yes
- Reason: No other apparent sources of bias.

Please let me know if you would like me to evaluate any other outcomes from this RCT. I will be happy to follow the same criteria to assess their risk of bias.

© 2024 Lai H et al. *JAMA Network Open.*

ID: Murphy, 2012

First response:

Here is my evaluation of the risk of bias for the primary outcome of body fat percentage in the randomized controlled trial by Murphy et al. (2012):

Article ID: Murphy, 2012

Outcome Name: Body fat percentage

1. Was the allocation sequence adequately generated?

   - Probably yes

   - The method of sequence generation is not reported. However, the study states "Eligible volunteers were stratified according to gender, BMI and age and randomly allocated to one of two groups. Randomization was by minimization." Minimization involves some random element, so the sequence was probably adequately generated.

2. Was allocation adequately concealed?

   - Probably no

   - Details of allocation concealment are not reported.

3. Blinding: Was knowledge of the allocated interventions adequately prevented?

3a. Were patients blinded?

   - Probably no

   - Details of blinding are not reported.

3b. Were healthcare providers blinded?

   - Probably no

   - Details of blinding are not reported.

© 2024 Lai H et al. *JAMA Network Open.*

3c. Were data collectors blinded?

- Probably no
- Details of blinding are not reported.

3d. Were outcome assessors blinded?

- Probably no
- Details of blinding are not reported.

3e. Were data analysts blinded?

- Probably no
- Details of blinding are not reported.

4. Was loss to follow-up infrequent?

- Probably yes
- 20 of 164 participants (12%) withdrew or were lost to follow-up. The follow-up rate was 88%, so loss to follow-up can probably be considered infrequent.

5. Are reports of the study free of selective outcome reporting?

- Probably yes
- The protocol is not available, but the pre-specified primary outcome of body fat percentage appears to be fully reported.

6. Was the study apparently free of other problems that could put it at risk of bias?

- Probably yes
- No other apparent biases were identified.

© 2024 Lai H et al. *JAMA Network Open.*

ID: Murphy, 2012

Second response:

Here are the evaluation results for the reported outcome of percent body fat in the study by Murphy et al., 2012:

Article ID: Murphy et al., 2012

Outcome Name: Percent body fat

1. Was the allocation sequence adequately generated?

- Probably no. No details are provided on how the randomization sequence was generated.

2. Was the allocation adequately concealed?

- Probably no. No details are provided on allocation concealment.

3. Blinding: Was knowledge of the allocated interventions adequately prevented?

3a. Were patients blinded? - Probably no. No detail is available to support the assessment.

3b. Were healthcare providers blinded? - Probably no. No detail is available to support the assessment.

3c. Were data collectors blinded? - Probably no. No detail is available to support the assessment.

3d. Were outcome assessors blinded? - Probably no. No detail is available to support the assessment.

3e. Were data analysts blinded? - Probably no. No detail is available to support the assessment.

4. Was loss to follow-up infrequent?

- Probably no. No detail is available to support the assessment.

5. Are reports of the study free of selective outcome reporting?

- Probably yes. The study protocol is not available but it is clear that the published reports include the pre-specified primary outcome of percent body fat.

6. Was the study apparently free of other problems that could put it at a risk of bias?

© 2024 Lai H et al. *JAMA Network Open.*

- Probably yes. The study appears to be free of other sources of bias.

In summary, this study is at high risk of bias due to lack of details on randomization, allocation concealment, and blinding. Loss to follow-up and selective outcome reporting do not seem to be major issues. The assessment is based solely on the information reported in the article, without extrapolation or speculation.

© 2024 Lai H et al. *JAMA Network Open.*

ID: Poddar, 2013

First response:

Here are the risk of bias assessments for the RCT reported in Poddar et al. 2013:

Article ID: Poddar et al. 2013

Outcome Name: Weight loss

1. Was the allocation sequence adequately generated?

- Response: Probably yes

- Reason: The paper states "Prior to Visit 1, eligible participants were randomized into one of two treatment groups: weight loss using a USDA Food Guide Pyramid-based diet prescription (“standard diet”), or the same diet, plus the recommended mushroom substitution (“mushroom diet”) (Fig. 1)." However, no details are provided on how the randomization sequence was generated.

2. Was allocation adequately concealed?

- Response: Probably no

- Reason: No details are provided on allocation concealment.

3. Blinding:

3a. Were patients blinded?

- Response: Definitely no

- Reason: The paper states "though it was not possible to blind the participants to the foods they were being asked to consume."

3b. Were healthcare providers blinded?

- Response: Probably yes

- Reason: The paper states "the study counselor was not made aware of the group to which the participant had been randomized, and the participants were instructed not to disclose their diet assignment to the counselor."

© 2024 Lai H et al. *JAMA Network Open.*

3c. Were data collectors blinded?

- Response: Probably no

- Reason: No details provided.

3d. Were outcome assessors blinded?

- Response: Probably no

- Reason: No details provided.

3e. Were data analysts blinded?

- Response: Probably no

- Reason: No details provided.

4. Was loss to follow-up infrequent?

- Response: Probably no

- Reason: 83 of 209 participants (40%) dropped out during the course of the study. No details provided on reasons for missing data.

5. Are reports of the study free of selective outcome reporting?

- Response: Probably yes

- Reason: The study protocol is not available, but it appears that the expected outcomes are reported.

6. Was the study apparently free of other problems that could put it at a risk of bias?

- Response: Probably yes

- Reason: The study appears to be free of other sources of bias.

Outcome Name: Body composition

1. Was the allocation sequence adequately generated?

© 2024 Lai H et al. *JAMA Network Open.*

- Same response and reason as for Weight loss outcome

2. Was allocation adequately concealed?

    - Same response and reason as for Weight loss outcome

3. Blinding:

    - Same responses and reasons as for Weight loss outcome

4. Was loss to follow-up infrequent?

    - Same response and reason as for Weight loss outcome

5. Are reports of the study free of selective outcome reporting?

    - Same response and reason as for Weight loss outcome

6. Was the study apparently free of other problems that could put it at a risk of bias?

    - Same response and reason as for Weight loss outcome

Outcome Name: Dietary intake

1. Was the allocation sequence adequately generated?

    - Same response and reason as for Weight loss outcome

2. Was allocation adequately concealed?

    - Same response and reason as for Weight loss outcome

3. Blinding:

    - Same responses and reasons as for Weight loss outcome

4. Was loss to follow-up infrequent?

    - Same response and reason as for Weight loss outcome

© 2024 Lai H et al. *JAMA Network Open.*

5. Are reports of the study free of selective outcome reporting?

  - Same response and reason as for Weight loss outcome

6. Was the study apparently free of other problems that could put it at a risk of bias?

  - Same response and reason as for Weight loss outcome

Outcome Name: Blood parameters

1. Was the allocation sequence adequately generated?

  - Same response and reason as for Weight loss outcome

2. Was allocation adequately concealed?

  - Same response and reason as for Weight loss outcome

3. Blinding:

  - Same responses and reasons as for Weight loss outcome

4. Was loss to follow-up infrequent?

  - Same response and reason as for Weight loss outcome

5. Are reports of the study free of selective outcome reporting?

  - Same response and reason as for Weight loss outcome

6. Was the study apparently free of other problems that could put it at a risk of bias?

  - Same response and reason as for Weight loss outcome

© 2024 Lai H et al. *JAMA Network Open.*

ID: Poddar, 2013

Second response:

Here are the risk of bias assessments for the RCT report by Poddar et al. 2013:

Article ID: Poddar et al., 2013

Outcome Name: Body weight

1. Was the allocation sequence adequately generated?

- Probably yes

- The article states "Prior to Visit 1, eligible participants were randomized into one of two treatment groups." But no details are provided on how the randomization sequence was generated.

2. Was allocation adequately concealed?

- Probably no

- No details are provided on allocation concealment.

3. Blinding: Was knowledge of the allocated interventions adequately prevented?

3a. Were patients blinded?

- Probably no

- No details are provided on blinding of patients.

3b. Were healthcare providers blinded?

- Probably no

- No details are provided on blinding of healthcare providers.

3c. Were data collectors blinded?

- Probably no

- No details are provided on blinding of data collectors.

3d. Were outcome assessors blinded?

- Probably no

- No details are provided on blinding of outcome assessors.

© 2024 Lai H et al. *JAMA Network Open.*

3e. Were data analysts blinded?

- Probably no
- No details are provided on blinding of data analysts.

4. Was loss to follow-up (missing outcome data) infrequent?

- Probably no
- No details are provided to assess missing outcome data.

5. Are reports of the study free of selective outcome reporting?

- Probably yes
- The study reported body weight as a pre-specified outcome.

6. Was the study apparently free of other problems that could put it at a risk of bias?

- Probably yes
- The study appears to be free of other sources of bias based on the information provided.

Article ID: Poddar et al., 2013

Outcome Name: BMI

1. Was the allocation sequence adequately generated?

- Probably yes
- The article states "Prior to Visit 1, eligible participants were randomized into one of two treatment groups." But no details are provided on how the randomization sequence was generated.

2. Was allocation adequately concealed?

- Probably no
- No details are provided on allocation concealment.

3. Blinding: Was knowledge of the allocated interventions adequately prevented?

© 2024 Lai H et al. *JAMA Network Open.*

3a. Were patients blinded?

- Probably no
- No details are provided on blinding of patients.

3b. Were healthcare providers blinded?

- Probably no
- No details are provided on blinding of healthcare providers.

3c. Were data collectors blinded?

- Probably no
- No details are provided on blinding of data collectors.

3d. Were outcome assessors blinded?

- Probably no
- No details are provided on blinding of outcome assessors.

3e. Were data analysts blinded?

- Probably no
- No details are provided on blinding of data analysts.

4. Was loss to follow-up (missing outcome data) infrequent?

- Probably no
- No details are provided to assess missing outcome data.

5. Are reports of the study free of selective outcome reporting?

- Probably yes
- BMI was reported as a pre-specified outcome.

6. Was the study apparently free of other problems that could put it at a risk of bias?

- Probably yes
- The study appears to be free of other sources of bias based on the information provided.

Please let me know if you would like me to assess any other outcomes from this study.

© 2024 Lai H et al. *JAMA Network Open.*

ID: Turner-McGrievy, 2015

First response:

Here are the risk of bias assessments for the Turner-McGrievy et al. (2015) study:

Article ID: Turner-McGrievy, 2015

Outcome Name: Weight loss

1. Was the allocation sequence adequately generated?

 - Probably yes

 - "Participants were then randomized to one of the five diets using a computerized random-number generator"

2. Was the allocation adequately concealed?

 - Probably no

 - No details provided on allocation concealment

3. Blinding: Was knowledge of the allocated interventions adequately prevented?

3a. Were patients blinded?

 - Probably no

 - No details provided on blinding of patients

3b. Were healthcare providers blinded?

 - Probably no

 - No details provided on blinding of healthcare providers

3c. Were data collectors blinded?

 - Probably no

 - No details provided on blinding of data collectors

© 2024 Lai H et al. *JAMA Network Open.*

3d. Were outcome assessors blinded?

- Probably no
- No details provided on blinding of outcome assessors

3e. Were data analysts blinded?

- Probably no
- No details provided on blinding of data analysts

4. Was loss to follow-up (missing outcome data) infrequent?

- Probably yes
- "At the six-month time point, 50 (79%) completed the study (i.e., provided a body-weight measurement at 6 months)"

5. Are reports of the study free of selective outcome reporting?

- Probably yes
- All expected outcomes appear to be reported

6. Was the study apparently free of other problems that could put it at a risk of bias?

- Probably yes
- The study appears to be free of other sources of bias

Outcome Name: Dietary intake

1. Was the allocation sequence adequately generated?

- Probably yes
- "Participants were then randomized to one of the five diets using a computerized random-number generator"

2. Was the allocation adequately concealed?

© 2024 Lai H et al. *JAMA Network Open.*

- Probably no
- No details provided on allocation concealment

3. Blinding: Was knowledge of the allocated interventions adequately prevented?

3a. Were patients blinded?

- Probably no
- No details provided on blinding of patients

3b. Were healthcare providers blinded?

- Probably no
- No details provided on blinding of healthcare providers

3c. Were data collectors blinded?

- Probably no
- No details provided on blinding of data collectors

3d. Were outcome assessors blinded?

- Probably no
- No details provided on blinding of outcome assessors

3e. Were data analysts blinded?

- Probably no
- No details provided on blinding of data analysts

4. Was loss to follow-up (missing outcome data) infrequent?

- Probably yes
- "At six months, 49 (78%) completed two days of dietary recalls"

5. Are reports of the study free of selective outcome reporting?

© 2024 Lai H et al. *JAMA Network Open.*

- Probably yes
- All expected outcomes appear to be reported

6. Was the study apparently free of other problems that could put it at a risk of bias?
   - Probably yes
   - The study appears to be free of other sources of bias

© 2024 Lai H et al. *JAMA Network Open.*

ID: Turner-McGrievy, 2015

Second response:

Here is my evaluation of the randomized controlled trial by Turner-McGrievy et al. (2015) for the primary outcome of weight loss:

Article ID: Turner-McGrievy et al., 2015

Outcome Name: Weight loss

1. Was the allocation sequence adequately generated?

   - Definitely yes

   - "Participants were randomized to one of the five diets using a computerized random-number generator"

2. Was the allocation adequately concealed?

   - Probably no

   - The method of concealment is not explicitly stated.

3. Blinding: Was knowledge of the allocated interventions adequately prevented?

3a. Were patients blinded?

   - Definitely no

   - Patients were not blinded to their diet group assignment.

3b. Were healthcare providers blinded?

   - Definitely no

   - "Two registered dietitians with graduate degrees and expertise in all the study diets led the classes." The dietitians delivering the interventions were aware of diet assignments.

3c. Were data collectors blinded?

© 2024 Lai H et al. *JAMA Network Open.*

- Probably no

- Blinding of data collectors is not mentioned.

3d. Were outcome assessors blinded?

- Probably no

- Blinding of outcome assessors is not mentioned.

3e. Were data analysts blinded?

- Probably no

- Blinding of data analysts is not mentioned.

4. Was loss to follow-up (missing outcome data) infrequent?

- Definitely yes

- "At the six-month time point, 50 (79%) completed the study." The follow-up rate was >80% at the final time point.

5. Are reports of the study free of selective outcome reporting?

- Probably yes

- The study protocol is not available, but the published report appears to include the expected outcomes.

6. Was the study apparently free of other problems that could put it at a risk of bias?

- Definitely yes

- The study appears to be free of other sources of bias.

In summary, this randomized trial has low risks of bias from adequate random sequence generation, allocation concealment, and low missing outcome data. However, there are high risks of bias due to lack of blinding of patients, providers, data collectors, outcome assessors, and data analysts. Selective outcome reporting is unlikely.

© 2024 Lai H et al. *JAMA Network Open.*

ID: Yaskolka, 2019

First response:

Here are the risk of bias assessments for the article by Yaskolka Meir et al., 2019:

Article ID: Yaskolka Meir et al., 2019

Outcome Name: Changes in serum iron

1. Was the allocation sequence adequately generated?

- Response: Probably yes
- Reason: The study states "Participants were randomly assigned in 2 independent cycles (n = 25 each) according to Pharmaseed SOP 027 and the study was performed in compliance with The Israel Board for Animal Experiments guidelines (approval IL-16-07-221)." While it mentions randomization, details are not provided on how the random sequence was generated.

2. Was the allocation adequately concealed?

- Response: Probably no
- Reason: No details are provided on allocation concealment.

3. Blinding: Was knowledge of the allocated interventions adequately prevented?

3a. Were patients blinded?

- Response: Probably no
- Reason: No details are provided on blinding of patients/animals.

3b. Were healthcare providers blinded?

- Response: Probably no
- Reason: No details are provided on blinding of healthcare providers.

3c. Were data collectors blinded?

© 2024 Lai H et al. *JAMA Network Open.*

- Response: Probably no

- Reason: No details are provided on blinding of data collectors.

3d. Were outcome assessors blinded?

- Response: Probably no

- Reason: No details are provided on blinding of outcome assessors.

3e. Were data analysts blinded?

- Response: Probably no

- Reason: No details are provided on blinding of data analysts.

4. Was loss to follow-up (missing outcome data) infrequent?

- Response: Probably yes

- Reason: The study states "The trial was completed with 47 rats (1 rat each was excluded from the Mankai 50, Mankai 50-C, and Mankai 80 groups) owing to sporadic and non–treatment-related events." The low number of exclusions unlikely to impact the results.

5. Are reports of the study free of selective outcome reporting?

- Response: Probably yes

- Reason: The pre-specified outcomes appear to be reported as expected.

6. Was the study apparently free of other problems that could put it at a risk of bias?

- Response: Probably yes

- Reason: The study appears free of other sources of bias.

Outcome Name: Changes in hemoglobin

1. Was the allocation sequence adequately generated?

- Response: Probably yes

- Reason: The study states "Participants were randomly assigned in 2 independent cycles (n = 25 each) according to Pharmaseed SOP 027 and the study was performed in compliance with The Israel Board for Animal Experiments guidelines (approval IL-16-07-221)." While it mentions randomization, details are not provided on how the random sequence was generated.

2. Was the allocation adequately concealed?

- Response: Probably no
- Reason: No details are provided on allocation concealment.

3. Blinding: Was knowledge of the allocated interventions adequately prevented?

3a. Were patients blinded?

- Response: Probably no
- Reason: No details are provided on blinding of patients/animals.

3b. Were healthcare providers blinded?

- Response: Probably no
- Reason: No details are provided on blinding of healthcare providers.

3c. Were data collectors blinded?

- Response: Probably no
- Reason: No details are provided on blinding of data collectors.

3d. Were outcome assessors blinded?

- Response: Probably no
- Reason: No details are provided on blinding of outcome assessors.

3e. Were data analysts blinded?

- Response: Probably no
- Reason: No details are provided on blinding of data analysts.

© 2024 Lai H et al. *JAMA Network Open.*

4. Was loss to follow-up (missing outcome data) infrequent?

- Response: Probably yes
- Reason: The study states "The trial was completed with 47 rats (1 rat each was excluded from the Mankai 50, Mankai 50-C, and Mankai 80 groups) owing to sporadic and non–treatment-related events." The low number of exclusions unlikely to impact the results.

5. Are reports of the study free of selective outcome reporting?

- Response: Probably yes
- Reason: The pre-specified outcomes appear to be reported as expected.

6. Was the study apparently free of other problems that could put it at a risk of bias?

- Response: Probably yes
- Reason: The study appears free of other sources of bias.

© 2024 Lai H et al. *JAMA Network Open.*

ID: Yaskolka, 2019

Second response:

Here are the risk of bias assessments for the randomized controlled trial by Yaskolka Meir et al. 2019:

Article ID: Yaskolka Meir et al., 2019

Outcome Name: Changes in serum iron

1. Was the allocation sequence adequately generated?

- Definitely yes

- "Participants were randomly assigned to 1 of the 3 following groups: PA, PA + MED diet, or PA + green-MED diet"

2. Was allocation adequately concealed?

- Probably no

- No details provided

3. Blinding: Was knowledge of the allocated interventions adequately prevented?

3a. Were patients blinded?

- Probably no

- No details provided

3b. Were healthcare providers blinded?

- Probably no

- No details provided

3c. Were data collectors blinded?

- Probably no

- No details provided

3d. Were outcome assessors blinded?

- Probably no

- No details provided

© 2024 Lai H et al. *JAMA Network Open.*

3e. Were data analysts blinded?

- Probably no

- No details provided

4. Was loss to follow-up infrequent?

- Probably yes

- 294 participants enrolled, 264 completed 6-month follow-up, follow-up rate 89.8%

5. Are reports of the study free of selective outcome reporting?

- Probably yes

- The study protocol is not available, but it appears that the expected outcomes were reported.

6. Was the study apparently free of other problems that could put it at a risk of bias?

- Probably yes

- The study appears to be free of other sources of bias.

Outcome Name: Changes in blood hemoglobin

1. Was the allocation sequence adequately generated?

- Definitely yes

- "Participants were randomly assigned to 1 of the 3 following groups: PA, PA + MED diet, or PA + green-MED diet"

2. Was allocation adequately concealed?

- Probably no

- No details provided

3. Blinding: Was knowledge of the allocated interventions adequately prevented?

3a. Were patients blinded?

- Probably no

© 2024 Lai H et al. *JAMA Network Open.*

- No details provided

3b. Were healthcare providers blinded?

- Probably no
- No details provided

3c. Were data collectors blinded?

- Probably no
- No details provided

3d. Were outcome assessors blinded?

- Probably no
- No details provided

3e. Were data analysts blinded?

- Probably no
- No details provided

4. Was loss to follow-up infrequent?

- Probably yes
- 294 participants enrolled, 264 completed 6-month follow-up, follow-up rate 89.8%

5. Are reports of the study free of selective outcome reporting?

- Probably yes
- The study protocol is not available, but it appears that the expected outcomes were reported.

6. Was the study apparently free of other problems that could put it at a risk of bias?

- Probably yes
- The study appears to be free of other sources of bias.

© 2024 Lai H et al. *JAMA Network Open.*

ID: Carlson, 2019

First response:

Thank you for the clear guidelines and examples for evaluating risk of bias in randomized controlled trials. I will carefully assess the Carlson et al. 2019 article focusing on the outcome of change in HbA1c, following the criteria you outlined:

Article ID: Carlson, 2019

Outcome Name: Change in HbA1c

1. Was the allocation sequence adequately generated?

- Probably no

- The article states "participants were randomized" but does not provide details on sequence generation.

2. Was the allocation adequately concealed?

- Probably no

- The article does not provide details on allocation concealment.

3. Blinding: Was knowledge of the allocated interventions adequately prevented?

3a. Were patients blinded? Probably no

3b. Were healthcare providers blinded? Probably no

3c. Were data collectors blinded? Probably yes

3d. Were outcome assessors blinded? Probably yes

3e. Were data analysts blinded? Probably yes

- The study is described as "open-label" so patients and providers were aware of treatment assignments. However, outcome assessment was likely blinded.

4. Was loss to follow-up (missing outcome data) infrequent?

- Probably yes

- Follow-up rates were good with 49/60 participants included in the analysis.

© 2024 Lai H et al. *JAMA Network Open.*

5. Are reports of the study free of selective outcome reporting?

- Probably no

- The study protocol is not available to compare with reported outcomes.

6. Was the study apparently free of other problems that could put it at a risk of bias?

- Probably yes

- The study appears free of other biases based on reported information.

Please let me know if you would like me to assess any other outcomes from this study. I will be happy to provide the risk of bias evaluation for additional outcomes following the guidelines you outlined.

© 2024 Lai H et al. *JAMA Network Open.*

ID: Carlson, 2019

Second response:

Here are the evaluation results for the Carlson et al. (2019) article:

Article ID: Carlson, 2019

Outcome Name: Hemoglobin A1c

1. Was the allocation sequence adequately generated?

- Response for the specific outcome: Probably no
- Reason for the specific outcome: Participants were randomized to treatment arms, but no details were provided on how the randomization sequence was generated.

2. Was the allocation adequately concealed?

- Response for the specific outcome: Probably no
- Reason for the specific outcome: No details were provided on allocation concealment.

3. Were patients blinded?

- Response for the specific outcome: Probably no
- Reason for the specific outcome: The study was open-label, so patients were not blinded.

3. Were healthcare providers blinded?

- Response for the specific outcome: Probably no
- Reason for the specific outcome: The study was open-label, so healthcare providers were not blinded.

3. Were data collectors blinded?

© 2024 Lai H et al. *JAMA Network Open.*

- Response for the specific outcome: Probably yes

- Reason for the specific outcome: Hemoglobin A1c measurement is unlikely to be influenced by lack of blinding.

3. Were outcome assessors blinded?

- Response for the specific outcome: Probably yes

- Reason for the specific outcome: Hemoglobin A1c assessment is unlikely to be influenced by lack of blinding.

3. Were data analysts blinded?

- Response for the specific outcome: Probably yes

- Reason for the specific outcome: Hemoglobin A1c analysis is unlikely to be influenced by lack of blinding.

4. Was loss to follow-up (missing outcome data) infrequent?

- Response for the specific outcome: Definitely yes

- Reason for the specific outcome: There were no missing hemoglobin A1c outcome data reported.

5. Are reports of the study free of selective outcome reporting?

- Response for the specific outcome: Probably yes

- Reason for the specific outcome: Hemoglobin A1c was a pre-specified outcome and was reported as expected.

6. Was the study apparently free of other problems that could put it at a risk of bias?

© 2024 Lai H et al. *JAMA Network Open.*

- Response for the specific outcome: Probably yes

- Reason for the specific outcome: No other apparent sources of bias were identified related to hemoglobin A1c outcome.

Outcome Name: Body Weight

1. Was the allocation sequence adequately generated?

- Response for the specific outcome: Probably yes

- Reason for the specific outcome: Participants were randomized to treatment arms, but no details were provided on how the randomization sequence was generated.

2. Was the allocation adequately concealed?

- Response for the specific outcome: Probably no

- Reason for the specific outcome: No details were provided on allocation concealment.

3. Were patients blinded?

- Response for the specific outcome: Probably no

- Reason for the specific outcome: The study was open-label, so patients were not blinded.

3. Were healthcare providers blinded?

- Response for the specific outcome: Probably no

- Reason for the specific outcome: The study was open-label, so healthcare providers were not blinded.

3. Were data collectors blinded?

- Response for the specific outcome: Probably no

© 2024 Lai H et al. *JAMA Network Open.*

- Reason for the specific outcome: Lack of blinding may influence body weight measurement.

3. Were outcome assessors blinded?

- Response for the specific outcome: Probably no
- Reason for the specific outcome: Lack of blinding may influence body weight assessment.

3. Were data analysts blinded?

- Response for the specific outcome: Probably yes
- Reason for the specific outcome: Body weight analysis is unlikely to be influenced by lack of blinding.

4. Was loss to follow-up (missing outcome data) infrequent?

- Response for the specific outcome: Probably yes
- Reason for the specific outcome: Missing body weight data appears balanced across groups with similar reasons for missing data.

5. Are reports of the study free of selective outcome reporting?

- Response for the specific outcome: Probably yes
- Reason for the specific outcome: Body weight was a pre-specified outcome and was reported as expected.

6. Was the study apparently free of other problems that could put it at a risk of bias?

- Response for the specific outcome: Probably yes
- Reason for the specific outcome: No other apparent sources of bias were identified related to body weight outcome.

© 2024 Lai H et al. *JAMA Network Open.*

ID: Cherney, 2021

First response:

Here are my evaluations of the risk of bias for the RCTs you provided:

Article ID: Cherney, 2021

Outcome Name: Change in HbA1c at 26 weeks

1. Was the allocation sequence adequately generated?

- Probably no

- The article states "Patients were randomly assigned in a 1:1:1 ratio to receive placebo, sotagliflozin 200 mg, or sotagliflozin 400 mg once daily in a double-blind fashion. Randomization was stratified by screening HbA1c (≤8.5% vs >8.5%) and mean systolic blood pressure (<130 mm Hg vs ≥130 mm Hg)." While it mentions randomization, no details are provided on how the randomization sequence was specifically generated.

2. Was allocation adequately concealed?

- Probably no

- No details are provided on allocation concealment.

3. Blinding: Was knowledge of the allocated interventions adequately prevented?

3a. Were patients blinded?

- Probably yes

- The article states this was a "double-blind, placebo-controlled trial", suggesting patients were likely blinded.

3b. Were healthcare providers blinded?

- Probably yes

- The article states this was a "double-blind, placebo-controlled trial", suggesting healthcare providers were likely blinded.

© 2024 Lai H et al. *JAMA Network Open.*

3c. Were data collectors blinded?

- Probably yes

- The article states this was a "double-blind, placebo-controlled trial", suggesting data collectors were likely blinded.

3d. Were outcome assessors blinded?

- Probably yes

- The article states "Laboratory values, including fasting plasma glucose (FPG), HbA1c, and urinary glucose excretion (UGE) were determined by a central laboratory and masked to study sites and patients from randomization until study end." This suggests outcome assessors were blinded.

3e. Were data analysts blinded?

- Probably yes

- The article states "Laboratory values, including fasting plasma glucose (FPG), HbA1c, and urinary glucose excretion (UGE) were determined by a central laboratory and masked to study sites and patients from randomization until study end." This suggests data analysts were likely blinded as well.

4. Was loss to follow-up (missing outcome data) infrequent?

- Probably yes

- The article states "All observed data were used in all efficacy analyses, including data gathered after study drug discontinuation or introduction of rescue therapy." This suggests efforts were made to minimize missing outcome data. The participant flow diagram shows relatively balanced attrition between groups.

5. Are reports of the study free of selective outcome reporting?

- Probably no

- The study protocol is not available to compare reported outcomes against pre-specified outcomes. Some key details around the statistical analysis plan are not reported, raising the possibility of selective reporting.

© 2024 Lai H et al. *JAMA Network Open.*

6. Was the study apparently free of other problems that could put it at a risk of bias?

- Probably yes
- No other apparent issues identified.

Article ID: Cherney, 2021

Outcome Name: Change in HbA1c at 52 weeks

1. Was the allocation sequence adequately generated?

- Probably yes
- The article states "Patients were randomly assigned in a 1:1:1 ratio to receive placebo, sotagliflozin 200 mg, or sotagliflozin 400 mg once daily in a double-blind fashion. Randomization was stratified by screening HbA1c (≤8.5% vs >8.5%) and mean systolic blood pressure (<130 mm Hg vs ≥130 mm Hg)." While it mentions randomization, no details are provided on how the randomization sequence was specifically generated.

2. Was allocation adequately concealed?

- Probably no
- No details are provided on allocation concealment.

3. Blinding: Was knowledge of the allocated interventions adequately prevented?

3a. Were patients blinded?

- Probably yes
- The article states this was a "double-blind, placebo-controlled trial", suggesting patients were likely blinded.

3b. Were healthcare providers blinded?

- Probably yes
- The article states this was a "double-blind, placebo-controlled trial", suggesting healthcare providers were likely blinded.

© 2024 Lai H et al. *JAMA Network Open.*

3c. Were data collectors blinded?

- Probably yes

- The article states this was a "double-blind, placebo-controlled trial", suggesting data collectors were likely blinded.

3d. Were outcome assessors blinded?

- Probably yes

- The article states "Laboratory values, including fasting plasma glucose (FPG), HbA1c, and urinary glucose excretion (UGE) were determined by a central laboratory and masked to study sites and patients from randomization until study end." This suggests outcome assessors were blinded.

3e. Were data analysts blinded?

- Probably yes

- The article states "Laboratory values, including fasting plasma glucose (FPG), HbA1c, and urinary glucose excretion (UGE) were determined by a central laboratory and masked to study sites and patients from randomization until study end." This suggests data analysts were likely blinded as well.

4. Was loss to follow-up (missing outcome data) infrequent?

- Probably yes

- The article states "All observed data were used in all efficacy analyses, including data gathered after study drug discontinuation or introduction of rescue therapy." This suggests efforts were made to minimize missing outcome data. The participant flow diagram shows relatively balanced attrition between groups.

5. Are reports of the study free of selective outcome reporting?

- Probably no

- The study protocol is not available to compare reported outcomes against pre-specified outcomes. Some key details around the statistical analysis plan are not reported, raising the possibility of selective reporting.

© 2024 Lai H et al. *JAMA Network Open.*

6. Was the study apparently free of other problems that could put it at a risk of bias?

- Probably yes

- No other apparent issues identified.

© 2024 Lai H et al. *JAMA Network Open.*

ID: Cherney, 2021

Second response:

Here is my evaluation of the methodological quality for the primary outcome in the given RCT:

Article ID: Cherney et al, 2021

Outcome Name: Change in HbA1c at Week 26 (primary outcome)

1. Was the allocation sequence adequately generated?

- Definitely yes

- The article states "Patients were randomly assigned in a 1:1:1 ratio to placebo, sotagliflozin 200 mg, or sotagliflozin 400 mg once daily by use of an interactive web-response system that implemented a computer-generated random sequence using randomly permuted blocks." This indicates adequate random sequence generation using computer-generated random numbers.

2. Was the allocation adequately concealed?

- Definitely yes

- The article states "Randomization was stratified by screening HbA1c (≤8.5% vs >8.5%) and mean SBP (<130 mm Hg vs ≥130 mm Hg). Laboratory values, including FPG, HbA1c, and UGE were determined by a central laboratory and masked to study sites and patients from randomization until study end." This indicates central allocation and adequate concealment of allocation.

3. Blinding: Was knowledge of the allocated interventions adequately prevented?

3a. Were patients blinded?

- Definitely yes

- The article states "This phase 3, multicenter, randomized, double-blind, placebo-controlled study...". This indicates patients were blinded.

3b. Were healthcare providers blinded?

- Definitely yes

© 2024 Lai H et al. *JAMA Network Open.*

- The article states "This phase 3, multicenter, randomized, double-blind, placebo-controlled study...". This indicates healthcare providers were blinded.

3c. Were data collectors blinded?

- Definitely yes

- The article states "Laboratory values, including FPG, HbA1c, and UGE were determined by a central laboratory and masked to study sites and patients from randomization until study end." This indicates blinding of data collectors.

3d. Were outcome assessors blinded?

- Definitely yes

- As the trial is described as double-blind and the central laboratory was blinded, the outcome assessors were also likely blinded.

3e. Were data analysts blinded?

- Definitely yes

- The article states "The primary multiple imputation algorithm applied to each endpoint was a mixture of the retrieved dropout and washout methods (see Supplementary Appendix). Each of the complete datasets following the multiple imputation step were analyzed using the ANCOVA model with treatment groups (sotagliflozin 400 mg, sotagliflozin 200 mg, placebo), randomization strata of HbA1c (≤8.5%, >8.5%) and SBP (<130 mmHg, ≥130 mmHg), and country as fixed effects, and the baseline endpoint value as a covariate." This indicates the data analysts were blinded during analysis.

4. Was loss to follow-up infrequent?

- Probably yes

- 93 patients were randomized to placebo and 75 completed 26 weeks. 94 patients were randomized to sotagliflozin 200mg and 78 completed 26 weeks. 92 patients were randomized to sotagliflozin 400mg and 77 completed 26 weeks. This indicates a follow-up rate of approximately 80-85% at 26 weeks. The reasons for discontinuation appear balanced across groups. Though the follow-up rate is

© 2024 Lai H et al. *JAMA Network Open.*

slightly below 90%, it is still reasonably high, so loss to follow-up can probably be considered infrequent.

5. Are reports of the study free of selective outcome reporting?

- Definitely yes
- The protocol does not appear to be available, but the pre-specified primary and secondary outcomes are reported as expected in the methods and results sections. There is no evidence of selective reporting.

6. Was the study apparently free of other problems that could put it at a risk of bias?

- Definitely yes
- The study appears to be free of other sources of bias.

© 2024 Lai H et al. *JAMA Network Open.*

ID: del Prato, 2014

First response:

Thank you for providing the detailed guidelines and examples for evaluating risk of bias in randomized controlled trials. I will follow the criteria closely and provide my assessment for the article you attached in the requested format. Please let me know if I have misunderstood any part of the instructions.

Article ID: Del Prato, 2014

Outcome Name: Change in HbA1c from baseline to week 104

1. Was the allocation sequence adequately generated?

- Probably no

- The article states "patients were randomized in a 1:1:1 ratio" but does not provide details on how the randomization sequence was generated.

2. Was the allocation adequately concealed?

- Probably no

- The article does not provide details on allocation concealment.

3. Blinding: Was knowledge of the allocated interventions adequately prevented?

3a. Were patients blinded?

- Probably yes

- The study is described as "double-blind" but details of blinding methods are not provided. Outcome not likely influenced by lack of blinding.

3b. Were healthcare providers blinded?

- Probably yes

- Same reason as 3a.

3c. Were data collectors blinded?

© 2024 Lai H et al. *JAMA Network Open.*

- Probably yes

- Same reason as 3a.

3d. Were outcome assessors blinded?

- Probably yes

- Same reason as 3a.

3e. Were data analysts blinded?

- Probably yes

- Same reason as 3a.

4. Was loss to follow-up (missing outcome data) infrequent?

- Probably yes

- Dropout rates were 20-24% across groups. Follow-up rate is between 80-90% at longest time point.

5. Are reports of the study free of selective outcome reporting?

- Probably yes

- Study protocol not available but appears all expected outcomes were reported.

6. Was the study apparently free of other problems that could put it at a risk of bias?

- Probably yes

- Study appears free of other sources of bias based on reported information.

Please let me know if you would like me to evaluate additional outcomes from this study. I will be happy to follow the instructions to assess other outcomes as well.

© 2024 Lai H et al. *JAMA Network Open.*

ID: del Prato, 2014

Second response:

Thank you for providing the detailed article and evaluation guidelines. Here is my assessment of the randomized controlled trial by Del Prato et al. (2014):

Article ID: Del Prato et al., 2014

Outcome Name: Change in HbA1c from baseline to week 104

1. Was the allocation sequence adequately generated?

   - Definitely yes

   - The article states "All patients were randomized in a 1:1:1 ratio" which indicates adequate random sequence generation.

2. Was the allocation adequately concealed?

   - Probably yes

   - The article states this was a "double-blind" study, implying efforts were made to conceal allocation, though the exact method is not specified.

3. Blinding: Was knowledge of the allocated interventions adequately prevented?

3a. Were patients blinded?

   - Probably yes

   - The study is described as "double-blind", implying patients were blinded, though details are not provided.

3b. Were healthcare providers blinded?

   - Probably yes

   - The study is described as "double-blind", implying providers were blinded, though details are not provided.

© 2024 Lai H et al. *JAMA Network Open.*

3c. Were data collectors blinded?

- Probably yes
- The study is described as "double-blind", implying data collectors were blinded, though details are not provided.

3d. Were outcome assessors blinded?

- Probably yes
- The study is described as "double-blind", implying outcome assessors were blinded, though details are not provided.

3e. Were data analysts blinded?

- Probably yes
- The study is described as "double-blind", implying data analysts were blinded, though details are not provided.

4. Was loss to follow-up infrequent?

- Probably yes
- 76-80% of patients completed the study. The dropout rate appears balanced across groups.

5. Are reports of the study free of selective outcome reporting?

- Probably yes
- The protocol does not appear to be available, but the prespecified primary outcome (HbA1c change at 104 weeks) is reported as expected.

6. Was the study apparently free of other problems that could put it at a risk of bias?

- Probably yes
- No other apparent biases noted.

© 2024 Lai H et al. *JAMA Network Open.*

In summary, this randomized trial appears to have taken reasonable steps to minimize bias, but some details around blinding and allocation concealment are lacking. There is no evidence of high risk of bias.

© 2024 Lai H et al. *JAMA Network Open.*

ID: Frias, 2018

First response:

Thank you for the detailed instructions and for explaining the role. As a seasoned expert in evidence-based medicine, I will conduct thorough and accurate assessments of the methodological quality of the randomized controlled trial. I will ensure my responses are correct, well-justified and do not speculate positively when there is insufficient information.

Here is the evaluation for the article:

Article ID: Frias et al., 2018

Outcome Name: Change in HbA1c from baseline to 26 weeks

1. Was the allocation sequence adequately generated?

- Response: Definitely yes
- Reason: "Participants who met the enrolment criteria were randomly allocated (1:1:1:1:1:1) to one of the six parallel treatment groups by use of an interactive web response system with three stratification variables: baseline HbA1c (<8.5% or ≥8.5%), metformin use (yes or no), and BMI (<30 kg/m2 or ≥30 kg/m2)."

2. Was the allocation adequately concealed?

- Response: Definitely yes
- Reason: "To ensure masking of patients and investigators from treatment identification due to the differences in volumes and titration regimes across the treatment groups, the patients in the placebo and dulaglutide groups were further randomised to the four LY3298176 dosing groups (1, 5, 10, and 15 mg)."

3. Blinding: Was knowledge of the allocated interventions adequately prevented?

3a. Were patients blinded?

© 2024 Lai H et al. *JAMA Network Open.*

- Response: Definitely yes

- Reason: "After a 1-week screening and 2-week lead-in period, participants were treated for 26 weeks and then followed-up for safety outcomes for 4 weeks (appendix). Participants treated with metformin or other pre-study medications continued to take these medications throughout the study."

3b. Were healthcare providers blinded?

- Response: Definitely yes

- Reason: "This 26-week, phase 2b, randomised, double-blind study was done at 47 sites (medical and clinical research centres) in Poland, Puerto Rico, Slovakia, and USA."

3c. Were data collectors blinded?

- Response: Definitely yes

- Reason: This study is described as double-blind, indicating data collectors were likely blinded.

3d. Were outcome assessors blinded?

- Response: Definitely yes

- Reason: This study is described as double-blind, indicating outcome assessors were likely blinded.

3e. Were data analysts blinded?

- Response: Definitely yes

- Reason: This study is described as double-blind, indicating data analysts were likely blinded.

4. Was loss to follow-up (missing outcome data) infrequent?

- Response: Definitely yes

- Reason: "The proportion of participants completing treatment was similar across the groups (range 82–86%), except for the 15 mg LY3298176 group, which had the lowest number of patients (66%) completing therapy." The follow-up rate was over 80% for all groups except one, indicating infrequent loss to follow-up.

© 2024 Lai H et al. *JAMA Network Open.*

5. Are reports of the study free of selective outcome reporting?

- Response: Definitely yes
- Reason: The outcomes reported match those pre-specified in the methods section, with no evidence of selective reporting.

6. Was the study apparently free of other problems that could put it at a risk of bias?

- Response: Definitely yes
- Reason: The study appears to be free of other sources of bias based on the information provided.

Please let me know if you would like me to evaluate any other outcomes from this study. I am happy to provide additional assessments as needed.

© 2024 Lai H et al. *JAMA Network Open.*

ID: Frias, 2018

Second response:

Article ID: Frias et al., 2018

Outcome Name: Change in HbA1c from baseline to 26 weeks

1. Was the allocation sequence adequately generated?

- Response: Definitely yes

- Reason: The study states, "Participants who met the enrolment criteria were randomly allocated (1:1:1:1:1:1) to one of the six parallel treatment groups by use of an interactive web response system with three stratification variables: baseline HbA1c (<8.5% or ≥8.5%), metformin use (yes or no), and BMI (<30 kg/m2 or ≥30 kg/m2)."

2. Was the allocation adequately concealed?

- Response: Definitely yes

- Reason: The study states, "To ensure masking of patients and investigators from treatment identification due to the differences in volumes and titration regimes across the treatment groups, the patients in the placebo and dulaglutide groups were further randomised to the four LY3298176 dosing groups (1, 5, 10, and 15 mg)."

3. Blinding: Was knowledge of the allocated interventions adequately prevented?

3a. Were patients blinded?

- Response: Definitely yes

- Reason: "After a 1-week screening and 2-week lead-in period, participants were treated for 26 weeks and then followed-up for safety outcomes for 4 weeks. Participants treated with metformin or other pre-study medications continued to take these medications throughout the study."

3b. Were healthcare providers blinded?

- Response: Definitely yes

© 2024 Lai H et al. *JAMA Network Open.*

- Reason: "This 26-week, phase 2b, randomised, double-blind study was done at 47 sites (medical and clinical research centres) in Poland, Puerto Rico, Slovakia, and USA."

3c. Were data collectors blinded?

- Response: Definitely yes
- Reason: This study is described as double-blind, indicating data collectors were likely blinded.

3d. Were outcome assessors blinded?

- Response: Definitely yes
- Reason: This study is described as double-blind, indicating outcome assessors were likely blinded.

3e. Were data analysts blinded?

- Response: Definitely yes
- Reason: This study is described as double-blind, indicating data analysts were likely blinded.

4. Was loss to follow-up (missing outcome data) infrequent?

- Response: Definitely yes
- Reason: "The proportion of participants completing treatment was similar across the groups (range 82–86%), except for the 15 mg LY3298176 group, which had the lowest number of patients (66%) completing therapy." The follow-up rate was over 80% for all groups except one, indicating infrequent loss to follow-up.

5. Are reports of the study free of selective outcome reporting?

- Response: Definitely yes
- Reason: The outcomes reported match those pre-specified in the methods section, with no evidence of selective reporting.

6. Was the study apparently free of other problems that could put it at a risk of bias?

- Response: Definitely yes

© 2024 Lai H et al. *JAMA Network Open.*

- Reason: The study appears to be free of other sources of bias based on the information provided.

© 2024 Lai H et al. *JAMA Network Open.*

ID: Gao, 2020

First response:

Article ID: Gao et al., 2020

Outcome Name: Change in HbA1c at 24 weeks

1. Was the allocation sequence adequately generated?

- Response: Probably no

- Reason: The article states, "Eligible patients were equally randomized to the following three groups: placebo, PEX168 100 µg, and PEX168 200 µg. Patients who were randomized to the placebo group were further randomized to receive 100 and 200 µg of PEX168 for 28 weeks after receiving placebo for the first 24 weeks." However, the exact method is not specified.

2. Was the allocation adequately concealed?

- Response: Probably yes

- Reason: The article states, "Randomization and drug container assignment were managed using a central randomization system." Central allocation generally implies adequate concealment.

3. Blinding: Was knowledge of the allocated interventions adequately prevented?

3a. Were patients blinded?

- Response: Probably yes

- Reason: The study is described as double-blind. The article states, "PEX168 and placebo injections were provided by the same manufacturer (Jiangsu Hansoh Pharmaceutical Group Co., Ltd., Jiangsu, China) with exactly the same packaging, labeling, appearance, taste, smell, and time of administration." This suggests efforts were made to blind patients.

3b. Were healthcare providers blinded?

- Response: Probably yes

- Reason: The study is described as double-blind, which implies blinding of healthcare providers.

© 2024 Lai H et al. *JAMA Network Open.*

3c. Were data collectors blinded?

- Response: Probably yes

- Reason: The study is described as double-blind, which implies blinding of data collectors.

3d. Were outcome assessors blinded?

- Response: Probably yes

- Reason: The study is described as double-blind, which implies blinding of outcome assessors.

3e. Were data analysts blinded?

- Response: Probably yes

- Reason: The article states, "From the beginning of randomization to the locking of the database, patients, investigators, evaluators, and data analysts were blinded to the treatment."

4. Was loss to follow-up (missing outcome data) infrequent?

- Response: Probably yes

- Reason: 174 out of 179 (97.2%) in the PEX168 100 μg group, 173 out of 175 (98.9%) in the PEX168 200 μg group, and 176 out of 179 (98.3%) in the placebo group completed the study. The dropout rate is low and balanced across groups.

5. Are reports of the study free of selective outcome reporting?

- Response: Probably yes

- Reason: The pre-specified primary and secondary outcomes are reported as described in the methods.

6. Was the study apparently free of other problems that could put it at a risk of bias?

- Response: Probably yes

- Reason: No other apparent biases noted.

© 2024 Lai H et al. *JAMA Network Open.*

© 2024 Lai H et al. *JAMA Network Open.*

ID: Gao, 2020

Second response:

Here are the evaluation results for the RCT by Gao et al. 2020:

Article ID: Gao, 2020

Outcome Name: Change in HbA1c from baseline to 24 weeks

1. Was the allocation sequence adequately generated?

- Probably no

- The article states "Eligible patients were equally randomized to the following three groups: placebo, PEX168 100 µg and PEX168 200 µg." However, details of the randomization method are not provided.

2. Was the allocation adequately concealed?

- Probably yes

- The article states "Randomization and drug container assignment were managed using a central randomization system." This suggests central allocation was used but details are not provided.

3. Blinding: Was knowledge of the allocated interventions adequately prevented?

3a. Were patients blinded?

- Probably yes

- The article states "Patients, investigators, evaluators and data analysts were blinded to the treatment." However, details of methods used to blind patients are not provided.

3b. Were healthcare providers blinded?

- Probably yes

- The article states "Patients, investigators, evaluators and data analysts were blinded to the treatment." However, details of methods used to blind providers are not provided.

© 2024 Lai H et al. *JAMA Network Open.*

3c. Were data collectors blinded?

- Probably yes

- The article states "Patients, investigators, evaluators and data analysts were blinded to the treatment." However, details of methods used to blind data collectors are not provided.

3d. Were outcome assessors blinded?

- Probably yes

- The article states "Patients, investigators, evaluators and data analysts were blinded to the treatment." However, details of methods used to blind outcome assessors are not provided.

3e. Were data analysts blinded?

- Probably yes

- The article states "Patients, investigators, evaluators and data analysts were blinded to the treatment."

4. Was loss to follow-up infrequent?

- Probably yes

- 573 patients were randomized and 533 were included in the full analysis set. The dropout rate was 7%, which is less than 20%.

5. Are reports of the study free of selective outcome reporting?

- Probably yes

- The study protocol is not available but the pre-specified primary outcome (change in HbA1c at 24 weeks) and secondary outcomes were reported.

6. Was the study apparently free of other problems that could put it at a risk of bias?

- Probably yes

- No other apparent sources of bias were identified based on the information provided.

© 2024 Lai H et al. *JAMA Network Open.*

ID: Ikonomidis, 2020

First response:

Based on the guidelines and information provided in the study by Ikonomidis et al. (2020), here are the evaluation results for the primary outcome of left ventricular global longitudinal strain (GLS):

Article ID: Ikonomidis et al., 2020

Outcome Name: Left ventricular global longitudinal strain (GLS)

1. Was the allocation sequence adequately generated?

- Response: Probably no

- Reason: No details are provided on how the randomization sequence was generated. The study just states that patients were randomized to the four treatment groups.

2. Was the allocation adequately concealed?

- Response: Probably no

- Reason: No details are provided on allocation concealment in the study.

3. Blinding: Was knowledge of the allocated interventions adequately prevented during the study?

3a. Were patients blinded? - Probably no

3b. Were healthcare providers blinded? - Probably no

3c. Were data collectors blinded? - Probably yes

3d. Were outcome assessors blinded? - Probably yes

3e. Were data analysts blinded? - Probably yes

- Reason for 3a-3b: The study states that patients and clinicians were blinded to the results of vascular and cardiac function tests, but it is not clear if patients and healthcare providers were actually blinded to the treatment allocation.

- Reason for 3c-3e: The echocardiography analyses were done by observers blinded to clinical and laboratory data.

4. Was loss to follow-up infrequent? - Probably yes

© 2024 Lai H et al. *JAMA Network Open.*

- Reason: 20 out of 180 participants (11%) did not complete the study. The dropout rate in each treatment arm ranged from 7.5% to 12.5%, which is less than 20%. This does not seem sufficient to have an important impact on the results.

5. Are reports of the study free of selective outcome reporting? - Probably yes

- Reason: The pre-specified primary outcome of GLS is reported. No evidence that expected outcomes were omitted.

6. Was the study apparently free of other problems that could put it at a risk of bias? - Probably yes

- Reason: The study appears to be free of other sources of bias based on the information provided.

© 2024 Lai H et al. *JAMA Network Open.*

ID: Ikonomidis, 2020

Second response:

Here is the evaluation for the RCT by Ikonomidis et al. (2020):

Article ID: Ikonomidis, 2020

Outcome Name: Left ventricularglobal longitudinal strain

1. Was the allocation sequence adequately generated?

- Response: Probably no

- Reason: The study states that patients were randomized to the 4 treatment groups, but does not describe the method of sequence generation.

2. Was the allocation adequately concealed?

- Response: Probably no

- Reason: The study does not mention any methods used to conceal the allocation.

3. Blinding: Was knowledge of the allocated interventions adequately prevented?

3a. Were patients blinded?

- Response: Definitely no

- Reason: The study does not mention blinding of patients.

3b. Were healthcare providers blinded?

- Response: Definitely no

- Reason: The study does not mention blinding of healthcare providers.

3c. Were data collectors blinded?

- Response: Definitely no

- Reason: The study does not mention blinding of data collectors.

3d. Were outcome assessors blinded?

- Response: Definitely no

- Reason: The study does not mention blinding of outcome assessors.

© 2024 Lai H et al. *JAMA Network Open.*

3e. Were data analysts blinded?

- Response: Definitely no

- Reason: The study does not mention blinding of data analysts.

4. Was loss to follow-up infrequent?

- Response: Probably yes

- Reason: 20 out of 180 participants (11%) did not complete the study. The dropout rates appear balanced across groups. This loss to follow-up is unlikely to have a significant impact on the results.

5. Are reports of the study free of selective outcome reporting?

- Response: Probably yes

- Reason: The study protocol is not available, but the published report appears to include the expected outcomes.

6. Was the study apparently free of other problems that could put it at a risk of bias?

- Response: Probably yes

- Reason: No other apparent biases were identified.

© 2024 Lai H et al. *JAMA Network Open.*

ID: Nahra, 2021

First response:

Based on the guidelines you provided, here are the risk of bias assessments for the randomized controlled trial by Nahra et al., 2021:

Article ID: Nahra, 2021

Outcome Name: Change in HbA1c at week 14

1. Was the allocation sequence adequately generated?

- Response: Definitely yes

- Reason: The article states "Participants were randomly assigned (overall ratio 4:9:9:4:4) to receive once-daily subcutaneous injections of cotadutide at 100 mg, 200 mg, or 300 mg; liraglutide 1.8 mg; or placebo. Assignment to treatment groups was determined by a computer-generated randomized sequence with use of interactive web response systems and interactive voice-response systems." This indicates adequate random sequence generation using computer generated random numbers.

2. Was the allocation adequately concealed?

- Response: Probably no

- Reason: No details were provided on allocation concealment.

3. Blinding: Was knowledge of the allocated interventions adequately prevented?

3a. Were patients blinded?

- Response: Probably yes

- Reason: The study is described as "double-blind" for cotadutide and placebo groups. Blinding of participants is likely.

3b. Were healthcare providers blinded?

- Response: Probably no

- Reason: Liraglutide was open-label, so healthcare providers were not blinded to this treatment arm.

© 2024 Lai H et al. *JAMA Network Open.*

3c. Were data collectors blinded?

- Response: Probably no

- Reason: No details provided on blinding of data collectors.

3d. Were outcome assessors blinded?

- Response: Probably no

- Reason: No details provided on blinding of outcome assessors.

3e. Were data analysts blinded?

- Response: Probably no

- Reason: No details provided on blinding of data analysts.

4. Was loss to follow-up infrequent?

- Response: Probably yes

- Reason: 78.2% participants completed the study. The dropout rate was <20% overall.

5. Are reports of the study free of selective outcome reporting?

- Response: Probably yes

- Reason: All expected outcomes appear to be reported.

6. Was the study apparently free of other problems that could put it at risk of bias?

- Response: Probably yes

- Reason: No other apparent issues noted.

Outcome Name: Percent change in body weight at week 14

1. Was the allocation sequence adequately generated?

- Response: Probably no

- Reason: No details provided on sequence generation.

© 2024 Lai H et al. *JAMA Network Open.*

2. Was the allocation adequately concealed?

- Response: Probably no

- Reason: No details provided on allocation concealment.

3. Blinding: Was knowledge of the allocated interventions adequately prevented?

3a. Were patients blinded?

- Response: Probably yes

- Reason: Double-blinding likely maintained blinding of participants.

3b. Were healthcare providers blinded?

- Response: Probably no

- Reason: Open-label liraglutide arm was not blinded.

3c. Were data collectors blinded?

- Response: Probably no

- Reason: No details provided on blinding of data collectors.

3d. Were outcome assessors blinded?

- Response: Probably no

- Reason: No details provided on blinding of outcome assessors.

3e. Were data analysts blinded?

- Response: Probably no

- Reason: No details provided on blinding of data analysts.

4. Was loss to follow-up infrequent?

- Response: Probably yes

- Reason: Overall dropout rate <20%.

5. Are reports of the study free of selective outcome reporting?

© 2024 Lai H et al. *JAMA Network Open.*

- Response: Probably yes

- Reason: All expected outcomes appear to be reported.

6. Was the study apparently free of other problems that could put it at risk of bias?

- Response: Probably yes

- Reason: No other issues identified.

Please let me know if you would like me to assess any other outcomes from this study. I'm happy to provide risk of bias judgements for additional outcomes following the same format.

© 2024 Lai H et al. *JAMA Network Open.*

ID: Nahra, 2021

Second response:

Based on the provided study details and role instructions, here is my assessment of the methodological quality for the outcome of change in HbA1c at week 14:

Article ID: Nahra, 2021

Outcome Name: Change in HbA1c at week 14

1. **Was the allocation sequence adequately generated?**

   - Definitely yes.

   - The article states "Participants were randomly assigned (overall ratio 4:9:9:4:4) to receive once-daily subcutaneous injections of cotadutide at 100 mg, 200 mg, or 300 mg; liraglutide 1.8 mg; or placebo. Assignment to treatment groups was determined by a computer-generated randomized sequence with use of interactive web response systems and interactive voice-response systems." This indicates adequate random sequence generation using computer generated random numbers.

2. **Was the allocation adequately concealed?**

   - Probably yes.

   - The article does not provide details on allocation concealment. However, since computer generated randomization was used, it is probable that allocation was adequately concealed.

3. **Blinding: Was knowledge of the allocated interventions adequately prevented?**

3a. **Were patients blinded?**

   - Definitely yes.

   - The article states "Administration of cotadutide or placebo with single-use prefilled syringes was double-blind; liraglutide administration, with commercially available multiuse 3-mL pen injectors, was open-label." This indicates that patients receiving cotadutide or placebo were blinded.

© 2024 Lai H et al. *JAMA Network Open.*

3b. **Were healthcare providers blinded?**

- Probably yes for cotadutide and placebo groups, Definitely no for liraglutide group.

- The article states that cotadutide and placebo administration was double-blinded, while liraglutide was open-label. This suggests healthcare providers were probably blinded for cotadutide and placebo groups but not for liraglutide group.

3c. **Were data collectors blinded?**

- Probably yes.

- The article does not provide information about blinding of data collectors. However, since this was a double-blind trial for cotadutide and placebo groups, it is probable that data collectors were also blinded.

3d. **Were outcome assessors blinded?**

- Probably yes.

- The article does not provide information about blinding of outcome assessors. However, since this was a double-blind trial for cotadutide and placebo groups, it is probable that outcome assessors were also blinded.

3e. **Were data analysts blinded?**

- Probably yes.

- The article does not provide information about blinding of data analysts. However, since this was a double-blind trial for cotadutide and placebo groups, it is probable that data analysts were also blinded.

4. **Was loss to follow-up (missing outcome data) infrequent?**

- Probably yes.

- The article does not report specific numbers for loss to follow-up at 14 weeks. However, Fig 1 shows that 4-5% of participants in each group discontinued the study by week 14. This dropout rate appears low and unlikely to impact results.

© 2024 Lai H et al. *JAMA Network Open.*

5. **Are reports of the study free of selective outcome reporting?**

- Probably yes.

- The study protocol is not available to verify. However, the pre-specified primary outcome of change in HbA1c at week 14 appears to be fully reported as planned. There is no indication of selective reporting.

6. **Was the study apparently free of other problems that could put it at risk of bias?**

- Definitely yes.

- The study appears to be free of other sources of bias.

In summary, this randomized controlled trial appears to have a low risk of bias for the outcome of change in HbA1c at week 14 based on the information reported. The computer generated randomization, double blinding of cotadutide and placebo groups, and low dropout rate help reduce risk of bias.

© 2024 Lai H et al. *JAMA Network Open.*

ID: Seino, 2015

First response:

Thank you for providing the detailed guidelines and instructions. I will follow them closely to evaluate the risk of bias for the given RCTs. Here are my assessments:

Article ID: Seino, 2015

Outcome Name: Change in HbA1c

1. Was the allocation sequence adequately generated?

- Response: Probably no

- Reason: The article states "Patients were randomized in a ratio of 2:1 to receive either luseogliflozin 2.5 mg or placebo." However, no details are provided on how the randomization sequence was generated.

2. Was the allocation adequately concealed?

- Response: Probably no

- Reason: The article does not provide details on allocation concealment.

3. Blinding:

3a. Were patients blinded?

- Response: Probably yes

- Reason: The study is described as "double-blind", suggesting attempts were made to blind patients. The outcome is unlikely to be influenced by lack of blinding.

3b. Were healthcare providers blinded?

- Response: Probably yes

- Reason: The study is described as "double-blind", suggesting attempts were made to blind healthcare providers. The outcome is unlikely to be influenced by lack of blinding.

3c. Were data collectors blinded?

- Response: Probably yes

© 2024 Lai H et al. *JAMA Network Open.*

- Reason: The study is described as "double-blind", suggesting attempts were made to blind data collectors. The outcome is unlikely to be influenced by lack of blinding.

3d. Were outcome assessors blinded?

- Response: Probably yes

- Reason: The study is described as "double-blind", suggesting attempts were made to blind outcome assessors. The outcome is unlikely to be influenced by lack of blinding.

3e. Were data analysts blinded?

- Response: Probably no

- Reason: The article does not provide details on blinding of data analysts.

4. Was loss to follow-up infrequent?

- Response: Probably yes

- Reason: 150 patients were randomized to the luseogliflozin group and 117 to placebo. 147 and 115 completed 24 weeks of treatment in each group. The follow-up rate is over 90% in both groups.

5. Are reports of the study free of selective outcome reporting?

- Response: Probably no

- Reason: The study protocol is not available to verify if all pre-specified outcomes were reported.

6. Was the study apparently free of other problems that could put it at a risk of bias?

- Response: Probably yes

- Reason: The study appears to be free of other sources of bias based on the information provided.

Article ID: Seino, 2015

Outcome Name: Change in body weight

1. Was the allocation sequence adequately generated?

- Response: Probably no

© 2024 Lai H et al. *JAMA Network Open.*

- Reason: The article states "Patients were randomized in a ratio of 2:1 to receive either luseogliflozin 2.5 mg or placebo." However, no details are provided on how the randomization sequence was generated.

2. Was the allocation adequately concealed?

- Response: Probably no

- Reason: The article does not provide details on allocation concealment.

3. Blinding:

3a. Were patients blinded?

- Response: Probably yes

- Reason: The study is described as "double-blind", suggesting attempts were made to blind patients. The outcome is unlikely to be influenced by lack of blinding.

3b. Were healthcare providers blinded?

- Response: Probably yes

- Reason: The study is described as "double-blind", suggesting attempts were made to blind healthcare providers. The outcome is unlikely to be influenced by lack of blinding.

3c. Were data collectors blinded?

- Response: Probably yes

- Reason: The study is described as "double-blind", suggesting attempts were made to blind data collectors. The outcome is unlikely to be influenced by lack of blinding.

3d. Were outcome assessors blinded?

- Response: Probably yes

- Reason: The study is described as "double-blind", suggesting attempts were made to blind outcome assessors. The outcome is unlikely to be influenced by lack of blinding.

3e. Were data analysts blinded?

© 2024 Lai H et al. *JAMA Network Open.*

- Response: Probably no

- Reason: The article does not provide details on blinding of data analysts.

4. Was loss to follow-up infrequent?

- Response: Probably yes

- Reason: 150 patients were randomized to the luseogliflozin group and 117 to placebo. 147 and 115 completed 24 weeks of treatment in each group. The follow-up rate is over 90% in both groups.

5. Are reports of the study free of selective outcome reporting?

- Response: Probably no

- Reason: The study protocol is not available to verify if all pre-specified outcomes were reported.

6. Was the study apparently free of other problems that could put it at a risk of bias?

- Response: Probably yes

- Reason: The study appears to be free of other sources of bias based on the information provided.

© 2024 Lai H et al. *JAMA Network Open.*

ID: Seino, 2015

Second response:

Here are my responses for the risk of bias assessment of the RCT provided:

Article ID: Seino, 2015

Outcome Name: HbA1c

1. Was the allocation sequence adequately generated?

- Response: Probably no

- Reason: The article states that patients were randomized in a 2:1 ratio to receive luseogliflozin or placebo, but no details are provided on how the randomization sequence was generated.

2. Was the allocation adequately concealed?

- Response: Probably no

- Reason: The article does not provide details on allocation concealment.

3. Blinding: Was knowledge of the allocated interventions adequately prevented?

3a. Were patients blinded?

- Response: Probably yes

- Reason: The study is described as double-blind, suggesting blinding of patients, though details are not provided. The outcome of HbA1c is not likely to be influenced by lack of blinding.

3b. Were healthcare providers blinded?

- Response: Probably yes

- Reason: The study is described as double-blind, suggesting blinding of healthcare providers, though details are not provided. The outcome of HbA1c is not likely to be influenced by lack of blinding.

3c. Were data collectors blinded?

- Response: Probably yes

© 2024 Lai H et al. *JAMA Network Open.*

- Reason: The study is described as double-blind, suggesting blinding of data collectors, though details are not provided. The outcome of HbA1c is not likely to be influenced by lack of blinding.

3d. Were outcome assessors blinded?

- Response: Probably yes

- Reason: The study is described as double-blind, suggesting blinding of outcome assessors, though details are not provided. The outcome of HbA1c is not likely to be influenced by lack of blinding.

3e. Were data analysts blinded?

- Response: Probably no

- Reason: No details provided on blinding of data analysts.

4. Was loss to follow-up (missing outcome data) infrequent?

- Response: Probably yes

- Reason: 222 patients were randomized and it appears 217 completed 24 weeks of treatment based on the patient disposition figure. The dropout rate appears less than 10%.

5. Are reports of the study free of selective outcome reporting?

- Response: Probably no

- Reason: The study protocol is not available to compare reported outcomes against pre-specified outcomes.

6. Was the study apparently free of other problems that could put it at a risk of bias?

- Response: Probably yes

- Reason: No other apparent sources of bias based on the information provided.

Article ID: Seino, 2015

Outcome Name: Body Weight

1. Was the allocation sequence adequately generated?

© 2024 Lai H et al. *JAMA Network Open.*

- Response: Probably no

- Reason: The article states that patients were randomized in a 2:1 ratio to receive luseogliflozin or placebo, but no details are provided on how the randomization sequence was generated.

2. Was the allocation adequately concealed?

- Response: Probably no

- Reason: The article does not provide details on allocation concealment.

3. Blinding: Was knowledge of the allocated interventions adequately prevented?

3a. Were patients blinded?

- Response: Probably yes

- Reason: The study is described as double-blind, suggesting blinding of patients, though details are not provided. The outcome of body weight is not likely to be influenced by lack of blinding.

3b. Were healthcare providers blinded?

- Response: Probably yes

- Reason: The study is described as double-blind, suggesting blinding of healthcare providers, though details are not provided. The outcome of body weight is not likely to be influenced by lack of blinding.

3c. Were data collectors blinded?

- Response: Probably yes

- Reason: The study is described as double-blind, suggesting blinding of data collectors, though details are not provided. The outcome of body weight is not likely to be influenced by lack of blinding.

3d. Were outcome assessors blinded?

- Response: Probably yes

- Reason: The study is described as double-blind, suggesting blinding of outcome assessors, though details are not provided. The outcome of body weight is not likely to be influenced by lack of blinding.

3e. Were data analysts blinded?

© 2024 Lai H et al. *JAMA Network Open.*

- Response: Probably no

- Reason: No details provided on blinding of data analysts.

4. Was loss to follow-up (missing outcome data) infrequent?

- Response: Probably yes

- Reason: 222 patients were randomized and it appears 217 completed 24 weeks of treatment based on the patient disposition figure. The dropout rate appears less than 10%.

5. Are reports of the study free of selective outcome reporting?

- Response: Probably no

- Reason: The study protocol is not available to compare reported outcomes against pre-specified outcomes.

6. Was the study apparently free of other problems that could put it at a risk of bias?

- Response: Probably yes

- Reason: No other apparent sources of bias based on the information provided.

© 2024 Lai H et al. *JAMA Network Open.*

ID: Taskinen, 2011

First response:

Article ID: Taskinen, 2011

Outcome Name: Change in HbA1c

1. Was the allocation sequence adequately generated?

- Probably no

- No details are provided on how the randomization sequence was generated.

2. Was the allocation adequately concealed?

- Probably no

- No details are provided on allocation concealment.

3. Blinding: Was knowledge of the allocated interventions adequately prevented?

3a. Were patients blinded?

- Probably yes

- The study is described as double-blind.

3b. Were healthcare providers blinded?

- Probably yes

- The study is described as double-blind.

3c. Were data collectors blinded?

- Probably yes

- The study is described as double-blind.

3d. Were outcome assessors blinded?

- Probably yes

- The study is described as double-blind.

© 2024 Lai H et al. *JAMA Network Open.*

3e. Were data analysts blinded?

- Probably yes

- The study is described as double-blind.

4. Was loss to follow-up (missing outcome data) infrequent?

- Probably yes

- 624/688 (91%) participants completed the study. The dropout rate is less than 10% in both groups.

5. Are reports of the study free of selective outcome reporting?

- Probably yes

- The pre-specified primary outcome of change in HbA1c is reported.

6. Was the study apparently free of other problems that could put it at a risk of bias?

- Probably yes

- The study appears to be free of other sources of bias.

Outcome Name: Change in FPG

1. Was the allocation sequence adequately generated?

- Probably no

- No details are provided on how the randomization sequence was generated.

2. Was the allocation adequately concealed?

- Probably no

- No details are provided on allocation concealment.

3. Blinding: Was knowledge of the allocated interventions adequately prevented?

3a. Were patients blinded?

- Probably yes

- The study is described as double-blind.

© 2024 Lai H et al. *JAMA Network Open.*

3b. Were healthcare providers blinded?

- Probably yes

- The study is described as double-blind.

3c. Were data collectors blinded?

- Probably yes

- The study is described as double-blind.

3d. Were outcome assessors blinded?

- Probably yes

- The study is described as double-blind.

3e. Were data analysts blinded?

- Probably yes

- The study is described as double-blind.

4. Was loss to follow-up (missing outcome data) infrequent?

- Probably yes

- 624/688 (91%) participants completed the study. The dropout rate is less than 10% in both groups.

5. Are reports of the study free of selective outcome reporting?

- Probably yes

- The pre-specified secondary outcome of change in FPG is reported.

6. Was the study apparently free of other problems that could put it at a risk of bias?

- Probably yes

- The study appears to be free of other sources of bias.

© 2024 Lai H et al. *JAMA Network Open.*

ID: Taskinen, 2011

Second response:

Thank you for providing the detailed guidelines and instructions. I will carefully follow them to evaluate the risk of bias for the given RCT article in the specified format.

Article ID: Taskinen, 2011

Outcome Name: Change in HbA1c

1. Was the allocation sequence adequately generated?

- Response: Probably no

- Reason: No details are provided on how the randomization sequence was generated.

2. Was the allocation adequately concealed?

- Response: Probably no

- Reason: No details are provided on allocation concealment.

3. Blinding: Was knowledge of the allocated interventions adequately prevented?

3a. Were patients blinded?

- Response: Probably yes

- Reason: The study is described as double-blind, so patients were likely blinded even though details are not provided.

3b. Were healthcare providers blinded?

- Response: Probably yes

- Reason: The study is described as double-blind, so healthcare providers were likely blinded even though details are not provided.

3c. Were data collectors blinded?

- Response: Probably yes

© 2024 Lai H et al. *JAMA Network Open.*

- Reason: The study is described as double-blind, so data collectors were likely blinded even though details are not provided.

3d. Were outcome assessors blinded?

- Response: Probably yes

- Reason: The study is described as double-blind, so outcome assessors were likely blinded even though details are not provided.

3e. Were data analysts blinded?

- Response: Probably yes

- Reason: The study is described as double-blind, so data analysts were likely blinded even though details are not provided.

4. Was loss to follow-up (missing outcome data) infrequent?

- Response: Probably yes

- Reason: 624/688 (91%) participants completed the study. The dropout rate is less than 10% in both groups.

5. Are reports of the study free of selective outcome reporting?

- Response: Probably yes

- Reason: All expected outcomes seem to be reported, but the study protocol is not available to confirm.

6. Was the study apparently free of other problems that could put it at a risk of bias?

- Response: Probably yes

- Reason: The study appears to be free of other sources of bias.

I have carefully evaluated each item based on the details provided in the RCT article and followed the guidelines you specified. Please let me know if you would like me to revise or expand my assessment in any way.

© 2024 Lai H et al. *JAMA Network Open.*

ID: Yabiku, 2017

First response:

Thank you for the detailed instructions and examples. Based on the guidelines you provided, here is my assessment of the randomized controlled trial by Yabiku et al. (2017):

Article ID: Yabiku, 2017

Outcome Name: Change in liver-to-spleen ratio on CT

1. Was the allocation sequence adequately generated?

- Response: Probably yes

- Reason: The article states "Patients with NAFLD were block randomized, with the block size set at 4 patients, using random-number generation software, into 4 groups..."

2. Was the allocation adequately concealed?

- Response: Probably no

- Reason: No details are provided on allocation concealment.

3. Blinding: Was knowledge of the allocated interventions adequately prevented?

3a. Were patients blinded?

- Response: Probably no

- Reason: The treatments were open label, so patients were not blinded.

3b. Were healthcare providers blinded?

- Response: Probably no

- Reason: The treatments were open label, so healthcare providers were not blinded.

3c. Were data collectors blinded?

- Response: Probably no

- Reason: No details provided on blinding of data collectors.

© 2024 Lai H et al. *JAMA Network Open.*

3d. Were outcome assessors blinded?

- Response: Probably no

- Reason: No details provided on blinding of outcome assessors.

3e. Were data analysts blinded?

- Response: Probably no

- Reason: No details provided on blinding of data analysts.

4. Was loss to follow-up (missing outcome data) infrequent?

- Response: Probably yes

- Reason: 7 patients withdrew from the control group, 5 withdrew from the metformin group, 5 withdrew from the sitagliptin group, and 9 withdrew from the pioglitazone group. The dropout rate is less than 10% in all groups.

5. Are reports of the study free of selective outcome reporting?

- Response: Probably yes

- Reason: The pre-specified primary outcome of change in L/S ratio appears to be reported.

6. Was the study apparently free of other problems that could put it at a risk of bias?

- Response: Probably yes

- Reason: The study appears free of other sources of bias based on the information provided.

Let me know if you would like me to evaluate additional outcomes from this study or assess the risk of bias for other articles. I am happy to follow your guidelines closely.

© 2024 Lai H et al. *JAMA Network Open.*

ID: Yabiku, 2017

Second response:

Based on the guidelines you provided, here is my assessment of the RCT:

Article ID: Yabiku, 2017

Outcome Name: Changes in the liver-to-spleen ratio on CT

1. Was the allocation sequence adequately generated?

- Response: Probably yes

- Reason: The article states "Patients with NAFLD were block randomized, with the block size set at 4 patients, using random-number generation software, into 4 groups."

2. Was the allocation adequately concealed?

- Response: Probably no

- Reason: No details are provided on allocation concealment.

3. Blinding: Was knowledge of the allocated interventions adequately prevented?

3a. Were patients blinded?

- Response: Probably no

- Reason: The study is described as open label, so patients were not blinded.

3b. Were healthcare providers blinded?

- Response: Probably no

- Reason: The study is described as open label, so healthcare providers were likely not blinded.

3c. Were data collectors blinded?

- Response: Probably no

- Reason: No details provided on blinding of data collectors.

3d. Were outcome assessors blinded?

- Response: Probably no

© 2024 Lai H et al. *JAMA Network Open.*

- Reason: No details provided on blinding of outcome assessors.

3e. Were data analysts blinded?

- Response: Probably no

- Reason: No details provided on blinding of data analysts.

4. Was loss to follow-up infrequent?

- Response: Probably yes

- Reason: 82-95% of participants in each group completed the study. The dropout rate is less than 20% in all groups.

5. Are reports of the study free of selective outcome reporting?

- Response: Probably no

- Reason: The study protocol is not available to compare reported outcomes against pre-specified outcomes.

6. Was the study apparently free of other problems that could put it at a risk of bias?

- Response: Probably no

- Reason: The study appears to have potential sources of bias due to lack of blinding of participants, healthcare providers, data collectors, outcome assessors and data analysts.

Please let me know if I have correctly followed the guidelines you provided for assessing risk of bias in this RCT. I am happy to clarify or revise my assessment if needed.

© 2024 Lai H et al. *JAMA Network Open.*

ID: Allen, 1987

First response:

Based on the evaluation criteria outlined in the introduction, here are my responses for the Allen 1987 study:

Article ID: Allen 1987

Outcome Name: Sleep Latency

1. Item name: 1. Was the allocation sequence adequately generated?

- Response: Probably no

- Reason: The article does not provide details on how the randomization sequence was generated.

2. Item name: 2. Was the allocation adequately concealed?

- Response: Probably no

- Reason: The article does not provide details on allocation concealment.

3. Item name: 3. Blinding: Was knowledge of the allocated interventions adequately prevented?

3.a. Were patients blinded?

- Response: Probably yes

- Reason: The study is described as double-blind, so patients were likely blinded even though details are not provided.

3.b. Were healthcare providers blinded?

- Response: Probably yes

© 2024 Lai H et al. *JAMA Network Open.*

- Reason: The study is described as double-blind, so healthcare providers were likely blinded even though details are not provided.

3.c. Were data collectors blinded?

- Response: Probably yes
- Reason: The study is described as double-blind, so data collectors were likely blinded even though details are not provided.

3.d. Were outcome assessors blinded?

- Response: Probably yes
- Reason: The study is described as double-blind, so outcome assessors were likely blinded even though details are not provided.

3.e. Were data analysts blinded?

- Response: Probably yes
- Reason: The study is described as double-blind, so data analysts were likely blinded even though details are not provided.

4. Item name: 4. Was loss to follow-up (missing outcome data) infrequent?

- Response: Probably yes
- Reason: 175 patients participated in phase 1. The number of patients included in the analyses ranged from 71 to 85 for midazolam and 75 to 90 for temazepam, indicating a follow-up rate of approximately 80-90% in phase 1. This is reasonable for the 1-month duration.

5. Item name: 5. Are reports of the study free of selective outcome reporting?

- Response: Probably yes
- Reason: The pre-specified primary outcome of sleep latency appears to be reported as expected.

© 2024 Lai H et al. *JAMA Network Open.*

6. Item name: 6. Was the study apparently free of other problems that could put it at a risk of bias?

- Response: Probably yes
- Reason: The study appears to be free of other sources of bias based on the information provided.

Outcome Name: Total Sleep Time

1. Item name: 1. Was the allocation sequence adequately generated?

- Response: Probably no
- Reason: The article does not provide details on how the randomization sequence was generated.

2. Item name: 2. Was the allocation adequately concealed?

- Response: Probably no
- Reason: The article does not provide details on allocation concealment.

3. Item name: 3. Blinding: Was knowledge of the allocated interventions adequately prevented?

3.a. Were patients blinded?
- Response: Probably yes
- Reason: The study is described as double-blind, so patients were likely blinded even though details are not provided.

3.b. Were healthcare providers blinded?
- Response: Probably yes
- Reason: The study is described as double-blind, so healthcare providers were likely blinded even though details are not provided.

3.c. Were data collectors blinded?

© 2024 Lai H et al. *JAMA Network Open.*

- Response: Probably yes

- Reason: The study is described as double-blind, so data collectors were likely blinded even though details are not provided.

3.d. Were outcome assessors blinded?

- Response: Probably yes

- Reason: The study is described as double-blind, so outcome assessors were likely blinded even though details are not provided.

3.e. Were data analysts blinded?

- Response: Probably yes

- Reason: The study is described as double-blind, so data analysts were likely blinded even though details are not provided.

4. Item name: 4. Was loss to follow-up (missing outcome data) infrequent?

- Response: Probably yes

- Reason: 175 patients participated in phase 1. The number of patients included in the analyses ranged from 71 to 85 for midazolam and 75 to 90 for temazepam, indicating a follow-up rate of approximately 80-90% in phase 1. This is reasonable for the 1-month duration.

5. Item name: 5. Are reports of the study free of selective outcome reporting?

- Response: Probably yes

- Reason: The pre-specified secondary outcome of total sleep time appears to be reported as expected.

6. Item name: 6. Was the study apparently free of other problems that could put it at a risk of bias?

- Response: Probably yes

- Reason: The study appears to be free of other sources of bias based on the information provided.

© 2024 Lai H et al. *JAMA Network Open.*

ID: Allen, 1987

Second response:

Based on the information provided in the Allen 1987 study, here is my assessment of the risk of bias for the outcome of sleep latency:

Article ID: Allen 1987

Outcome Name: Sleep Latency

1. Was the allocation sequence adequately generated?

- Probably no

- No details are provided on how randomization was carried out.

2. Was the allocation adequately concealed?

- Probably no

- The method of allocation concealment is not described.

3. Blinding: Was knowledge of the allocated interventions adequately prevented?

3a. Were patients blinded?

- Probably yes

- The study is described as "double-blind", suggesting patients were blinded. However, no details on blinding methods are provided.

3b. Were healthcare providers blinded?

- Probably yes

- The study is described as "double-blind", suggesting healthcare providers were blinded. However, no details on blinding methods are provided.

3c. Were data collectors blinded?

© 2024 Lai H et al. *JAMA Network Open.*

- Probably no

- Blinding of data collectors is not described.

3d. Were outcome assessors blinded?

- Probably no

- Blinding of outcome assessors is not described.

3e. Were data analysts blinded?

- Probably no

- Blinding of data analysts is not described.

4. Was loss to follow-up (missing outcome data) infrequent?

- Probably yes

- 17 patients out of 175 (9.7%) did not complete phase 1 of the study. Reasons for dropout appear evenly distributed between groups. This proportion of missing data may not have an important impact on effect estimates.

5. Are reports of the study free of selective outcome reporting?

- Probably yes

- The study protocol is not available, but the pre-specified outcomes appear to be reported as expected.

6. Was the study apparently free of other problems that could put it at risk of bias?

- Probably yes

- The study appears to be free of other sources of bias based on the information provided.

© 2024 Lai H et al. *JAMA Network Open.*

ID: Black, 2017

First response:

Based on the guidelines you provided, here are the risk of bias assessments for the two outcomes reported in the Black et al. (2017) randomized controlled trial:

Article ID: Black, 2017

Outcome Name: Objective WASO

1. Was the allocation sequence adequately generated?

- Response: Probably no

- Reason: The article states "Patients meeting study entry criteria were randomized 1:1:1:1 to one of four study oral treatments (placebo, almorexant 100 mg, almorexant 200 mg, or zolpidem 10 mg)." However, no details are provided on how the randomization sequence was generated.

2. Was allocation adequately concealed?

- Response: Probably no

- Reason: The article does not provide details on allocation concealment.

3. Blinding: Was knowledge of the allocated interventions adequately prevented?

3a. Were patients blinded?

- Response: Probably yes

- Reason: The article states this was a "double-blind" study, and blinding of participants is likely as medications were matched placebos.

3b. Were healthcare providers blinded?

- Response: Probably yes

- Reason: The article states this was a "double-blind" study, so blinding of providers is likely.

3c. Were data collectors blinded?

- Response: Probably yes

© 2024 Lai H et al. *JAMA Network Open.*

- Reason: The article states polysomnography data was "scored centrally" suggesting blinding of data collectors.

3d. Were outcome assessors blinded?

- Response: Probably yes

- Reason: The article states this was a "double-blind" study, so blinding of outcome assessors is likely.

3e. Were data analysts blinded?

- Response: Probably yes

- Reason: The article states this was a "double-blind" study, so blinding of data analysts is likely.

4. Was loss to follow-up infrequent?

- Response: Probably yes

- Reason: 93.8% of patients completed the study. The dropout rate is less than 20% in all groups.

5. Are reports of the study free of selective outcome reporting?

- Response: Probably no

- Reason: The study protocol is not available to verify all expected outcomes were reported.

6. Was the study apparently free of other problems that could put it at a risk of bias?

- Response: Probably yes

- Reason: The study appears free of other sources of bias based on the information provided.

Outcome Name: Subjective WASO

1. Was the allocation sequence adequately generated?

- Response: Probably no

- Reason: No details provided.

2. Was allocation adequately concealed?

© 2024 Lai H et al. *JAMA Network Open.*

- Response: Probably no

- Reason: No details provided.

3. Blinding: Was knowledge of the allocated interventions adequately prevented?

3a-e.

- Response: Probably yes

- Reason: Study stated to be double-blind.

4. Was loss to follow-up infrequent?

- Response: Probably yes

- Reason: 93.8% completion rate.

5. Are reports of the study free of selective outcome reporting?

- Response: Probably no

- Reason: Study protocol not available.

6. Was the study apparently free of other problems that could put it at a risk of bias?

- Response: Probably yes

- Reason: No other apparent issues.

Please let me know if you would like me to assess any other outcomes from the article or if you have any other questions!

© 2024 Lai H et al. *JAMA Network Open.*

ID: Black, 2017

Second response:

Here is my evaluation of the RCT for the outcome of objective wake time after sleep onset (WASO):

Article ID: Black et al., 2017

Outcome Name: Objective WASO

1. Was the allocation sequence adequately generated?

- Probably no

- The authors state that patients were "randomized 1:1:1:1 to one of four study oral treatments."

However, no details are provided on the specific method of random sequence generation.

2. Was allocation adequately concealed?

- Probably no

- No details provided to assess allocation concealment.

3. Blinding: Was knowledge of the allocated interventions adequately prevented?

3a. Were patients blinded?

- Probably yes

- The study is described as "double-blind" but details of blinding methods are not reported.

3b. Were healthcare providers blinded?

- Probably no

- No details provided.

3c. Were data collectors blinded?

- Probably no

- No details provided.

© 2024 Lai H et al. *JAMA Network Open.*

3d. Were outcome assessors blinded?

- Probably no

- No details provided.

3e. Were data analysts blinded?

- Probably no

- No details provided.

4. Was loss to follow-up (missing outcome data) infrequent?

- Probably yes

- 93.8% of randomized patients completed the study. Reasons for dropout appear balanced across groups.

5. Are reports of the study free of selective outcome reporting?

- Probably yes

- The protocol is not available but the pre-specified primary and secondary outcomes appear to be reported as expected.

6. Was the study apparently free of other problems that could put it at a risk of bias?

- Probably yes

- No other apparent sources of bias.

© 2024 Lai H et al. *JAMA Network Open.*

ID: Fan, 2017

First response:

Based on the guidelines provided, here is my evaluation of the randomized controlled trial by Fan et al. (2017):

Article ID: Fan, 2017

Outcome Name: Total sleep time (sTST)

1. Was the allocation sequence adequately generated?

- Response: Probably yes

- Reason: The article states "Eligible subjects were randomly allocated to either the suvorexant or the placebo group using a computerized number generator in the stratified block randomization method in SAS (version 8.2; SAS Institute, Inc., Cary, NC, USA)."

2. Was the allocation adequately concealed?

- Response: Probably yes

- Reason: The article states "The randomization was performed by a statistician from whom treatment allocation was masked. The treatment allocation was concealed in opaque sequentially numbered sealed envelopes containing the randomization assignments." This suggests adequate allocation concealment.

3. Blinding: Was knowledge of the allocated interventions adequately prevented?

3a. Were patients blinded?

- Response: Probably yes

- Reason: The study is described as double-blind, suggesting patients were blinded.

3b. Were healthcare providers blinded?

- Response: Probably yes

- Reason: The study is described as double-blind, suggesting healthcare providers were blinded.

© 2024 Lai H et al. *JAMA Network Open.*

3c. Were data collectors blinded?

- Response: Probably yes

- Reason: The study is described as double-blind, suggesting data collectors were blinded.

3d. Were outcome assessors blinded?

- Response: Probably yes

- Reason: The study is described as double-blind, suggesting outcome assessors were blinded.

3e. Were data analysts blinded?

- Response: Probably yes

- Reason: The article states "Data analysis was performed by a study statistician who was blind to the treatment allocation."

4. Was loss to follow-up infrequent?

- Response: Probably yes

- Reason: 114/120 participants completed the study. The follow-up rate is greater than 90%, so loss to follow-up can be considered infrequent.

5. Are reports of the study free of selective outcome reporting?

- Response: Probably yes

- Reason: The study protocol is not available to verify there is no selective outcome reporting, but the pre-specified primary and secondary outcomes appear to be reported as expected.

6. Was the study apparently free of other problems that could put it at a risk of bias?

- Response: Probably yes

- Reason: The study appears to be free of other sources of bias based on the information provided.

© 2024 Lai H et al. *JAMA Network Open.*

ID: Fan, 2017

Second response:

Article ID: Fan, 2017

Outcome Name: Total sleep time (sTST)

1. Was the allocation sequence adequately generated?

- Response: Probably yes

- Reason: The article states "Patients who met all the inclusion/exclusion criteria were assigned to either the suvorexant or the placebo group using a computerized number generator in the stratified block randomization method in SAS (version 8.2; SAS Institute, Inc., Cary, NC, USA)."

2. Was the allocation adequately concealed?

- Response: Probably yes

- Reason: The article states "The randomization was performed by a statistician from whom treatment allocation was masked. The treatment allocation was concealed in opaque sequentially numbered sealed envelopes containing the randomization assignments."

3. Blinding: Was knowledge of the allocated interventions adequately prevented?

3a. Were patients blinded?

- Response: Probably yes

- Reason: The article states "The patients, investigators, and study site personnel were blinded to the treatment allocation," suggesting patients were blinded.

3b. Were healthcare providers blinded?

- Response: Probably yes

- Reason: The article states "The patients, investigators, and study site personnel were blinded to the treatment allocation," suggesting healthcare providers were blinded.

3c. Were data collectors blinded?

- Response: Probably yes

© 2024 Lai H et al. *JAMA Network Open.*

- Reason: The article states "The patients, investigators, and study site personnel were blinded to the treatment allocation," suggesting data collectors were blinded.

3d. Were outcome assessors blinded?

- Response: Probably yes
- Reason: The article states "The patients, investigators, and study site personnel were blinded to the treatment allocation," suggesting outcome assessors were blinded.

3e. Were data analysts blinded?

- Response: Probably yes
- Reason: The article states "Data analysis was performed by a study statistician who was blind to the treatment allocation."

4. Was loss to follow-up infrequent?

- Response: Probably yes
- Reason: 114/120 participants completed the study. The follow-up rate is greater than 90%, so loss to follow-up can be considered infrequent.

5. Are reports of the study free of selective outcome reporting?

- Response: Probably yes
- Reason: The study protocol is not available to verify there is no selective outcome reporting, but the pre-specified primary and secondary outcomes appear to be reported as expected.

6. Was the study apparently free of other problems that could put it at a risk of bias?

- Response: Probably yes
- Reason: The study appears to be free of other sources of bias based on the information provided.

© 2024 Lai H et al. *JAMA Network Open.*

ID: Lankford, 2012

First response:

Based on the guidelines provided, here is my assessment of the risk of bias for the Lankford 2012 study:

Article ID: Lankford, 2012

Outcome Name: Subjective total sleep time (sTST)

1. Was the allocation sequence adequately generated?

- Response: Probably yes

- Reason: Computer-generated randomization was utilized.

2. Was the allocation adequately concealed?

- Response: Probably no

- Reason: No statements are provided on allocation concealment.

3. Blinding: Was knowledge of the allocated interventions adequately prevented?

3a. Were patients blinded?

- Response: Probably yes

- Reason: The study is described as double-blind.

3b. Were healthcare providers blinded?

- Response: Probably yes

- Reason: The study is described as double-blind.

3c. Were data collectors blinded?

- Response: Probably yes

- Reason: The study is described as double-blind.

3d. Were outcome assessors blinded?

© 2024 Lai H et al. *JAMA Network Open.*

- Response: Probably yes

- Reason: The study is described as double-blind.

3e. Were data analysts blinded?

- Response: Probably yes

- Reason: The study is described as double-blind.

4. Was loss to follow-up (missing outcome data) infrequent?

- Response: Probably yes

- Reason: 237/254 (93%) participants completed the study. The dropout rate is less than 10% in all groups.

5. Are reports of the study free of selective outcome reporting?

- Response: Probably yes

- Reason: The study protocol is not available to verify there is no selective outcome reporting, but the pre-specified primary and secondary outcomes appear to be reported as expected.

6. Was the study apparently free of other problems that could put it at a risk of bias?

- Response: Probably yes

- Reason: The study appears to be free of other sources of bias based on the information provided.

Please let me know if you would like me to assess the risk of bias for any other outcomes reported in this study. I will be happy to provide an evaluation for additional outcomes following the same format.

© 2024 Lai H et al. *JAMA Network Open.*

ID: Lankford, 2012

Second response:

Based on the guidelines you provided, here is my evaluation of the randomized controlled trial (RCT) in the Lankford et al. (2012) article:

Article ID: Lankford, 2012

Outcome Name: Subjective total sleep time (sTST) at week 1

1. Was the allocation sequence adequately generated?

- Response: Probably yes

- Reason: The article states "Eligible patients were assigned to one of two treatment groups (DXP 6 mg or placebo) in a 1:1 ratio according to a computer-generated randomization scheme." Computer-generated randomization is usually considered adequate.

2. Was allocation adequately concealed?

- Response: Probably no

- Reason: No statements are provided on allocation concealment.

3. Blinding:

3a. Patients blinded?

- Response: Probably yes

- Reason: The study is described as "double-blind" but details are not provided on who was blinded. However, the outcome of subjective total sleep time is patient-reported and unlikely to be influenced by lack of blinding.

3b. Healthcare providers blinded?

- Response: Probably yes

- Reason: Same as above.

3c. Data collectors blinded?

© 2024 Lai H et al. *JAMA Network Open.*

- Response: Probably yes
- Reason: Same as above.

3d. Outcome assessors blinded?
- Response: Probably yes
- Reason: Same as above.

3e. Data analysts blinded?
- Response: Probably yes
- Reason: Same as above.

4. Was loss to follow-up infrequent?
- Response: Probably yes
- Reason: 237/254 (93%) participants completed the study. The dropout rate is less than 10% in both groups.

5. Are reports of the study free of selective outcome reporting?
- Response: Probably yes
- Reason: The study protocol is not available, but the published report seems to include all expected outcomes with no evidence of selective reporting.

6. Was the study apparently free of other problems that could put it at a risk of bias?
- Response: Probably yes
- Reason: No other apparent sources of bias. The study appears to be free of other problems that could put it at a risk of bias.

Please let me know if you would like me to evaluate additional outcomes from this or other RCTs. I will be happy to follow the provided guidelines closely.

© 2024 Lai H et al. *JAMA Network Open.*

ID: Mignot, 2022

First response:

Thank you for the detailed guidelines and instructions. Let me know if my evaluation summary for the provided RCT meets your requirements:

Article ID: Mignot, 2022

Outcome Name: Change from baseline in wake time after sleep onset (WASO)

1. Was the allocation sequence adequately generated?

- Response: Probably yes

- Reason: The article states "Randomisation was stratified by age (<65 years and ≥65 years), and treatment was allocated using an interactive response technology system." This indicates some form of random sequence generation was used, though the exact method is not specified.

2. Was the allocation adequately concealed?

- Response: Probably yes

- Reason: The article states "Participants, investigators, site personnel, and sponsor-authorised personnel were unaware of treatment allocation." This suggests allocation was likely concealed, though specific methods are not described.

3. Blinding: Was knowledge of the allocated interventions adequately prevented?

3a. Were patients blinded?

- Response: Probably yes

- Reason: The article states "Participants...were unaware of treatment allocation" suggesting patients were blinded, though specific methods are not described.

3b. Were healthcare providers blinded?

- Response: Probably yes

- Reason: The article states "Investigators, site personnel...were unaware of treatment allocation" suggesting they were blinded, though specific methods are not described.

© 2024 Lai H et al. *JAMA Network Open.*

3c. Were data collectors blinded?

- Response: Probably yes

- Reason: No specific mention of blinding of data collectors, but given blinding of participants and site personnel, data collectors were likely also blinded.

3d. Were outcome assessors blinded?

- Response: Probably yes

- Reason: Polysomnography was centrally assessed by an independent scorer who was likely blinded though no specific mention.

3e. Were data analysts blinded?

- Response: Probably yes

- Reason: No specific mention of blinding of data analysts but given the overall blinding procedures described, data analysts were likely also blinded.

4. Was loss to follow-up infrequent?

- Response: Definitely yes

- Reason: Missing data was low (≤9.4% across all endpoints and groups) and appears balanced between groups.

5. Are reports of the study free of selective outcome reporting?

- Response: Probably yes

- Reason: The study protocol is not available, but the published report seems to include all expected outcomes with no evidence of selective reporting.

6. Was the study apparently free of other problems that could put it at a risk of bias?

- Response: Probably yes

- Reason: No other potential sources of bias identified based on the information provided.

© 2024 Lai H et al. *JAMA Network Open.*

Please let me know if this evaluation meets your requirements or if you need me to modify or expand on any part of it. I'm happy to revise as needed.

© 2024 Lai H et al. *JAMA Network Open.*

ID: Mignot, 2022

Second response:

Here are my evaluations of the two RCTs you provided:

Article ID: Mignot et al., 2022

Outcome Name: WASO

1. Was the allocation sequence adequately generated?

- Response: Probably yes

- Reason: The article states "Randomisation was stratified by age (<65 years and ≥65 years), and treatment was allocated using an interactive response technology system." While no specific details are provided on how the randomization sequence was generated, the use of an interactive response technology system suggests adequate sequence generation.

2. Was allocation adequately concealed?

- Response: Probably yes

- Reason: The article states "Participants, investigators, site personnel, and sponsor-authorised personnel were unaware of treatment allocation." While no specific details are provided, the double-blinding of key individuals suggests adequate concealment.

3.a. Were patients blinded?

- Response: Definitely yes

- Reason: The article states "Participants...were unaware of treatment allocation."

3.b. Were healthcare providers blinded?

- Response: Definitely yes

- Reason: The article states "Investigators [and] site personnel...were unaware of treatment allocation."

3.c. Were data collectors blinded?

- Response: Definitely yes

© 2024 Lai H et al. *JAMA Network Open.*

- Reason: The article states "Participants, investigators, site personnel, and sponsor-authorised personnel were unaware of treatment allocation." Data collectors would be included.

3.d. Were outcome assessors blinded?

- Response: Definitely yes

- Reason: The article states that polysomnography recordings were "centrally assessed by an independent scorer" who would have been blinded.

3.e. Were data analysts blinded?

- Response: Definitely yes

- Reason: The article states "Participants, investigators, site personnel, and sponsor-authorised personnel were unaware of treatment allocation." Data analysts would be included.

4. Was loss to follow-up infrequent?

- Response: Definitely yes

- Reason: Dropout rates were low, around 7-11% across groups at each timepoint. This is unlikely to impact results.

5. Are reports of the study free of selective outcome reporting?

- Response: Probably yes

- Reason: The study protocol is not available to verify, but outcomes reported match what would be expected for an insomnia trial.

6. Was the study apparently free of other problems that could put it at risk of bias?

- Response: Definitely yes

- Reason: No other apparent issues. Study appears to have been conducted well.

Article ID: Mignot et al., 2022

Outcome Name: LPS

1. Was the allocation sequence adequately generated?

© 2024 Lai H et al. *JAMA Network Open.*

- Response: Probably yes

- Reason: Same as for previous outcome

2. Was allocation adequately concealed?

- Response: Probably yes

- Reason: Same as for previous outcome

3.a. Were patients blinded?

- Response: Definitely yes

- Reason: Same as for previous outcome

3.b. Were healthcare providers blinded?

- Response: Definitely yes

- Reason: Same as for previous outcome

3.c. Were data collectors blinded?

- Response: Definitely yes

- Reason: Same as for previous outcome

3.d. Were outcome assessors blinded?

- Response: Definitely yes

- Reason: Same as for previous outcome

3.e. Were data analysts blinded?

- Response: Definitely yes

- Reason: Same as for previous outcome

4. Was loss to follow-up infrequent?

- Response: Definitely yes

- Reason: Same as for previous outcome

© 2024 Lai H et al. *JAMA Network Open.*

5. Are reports of the study free of selective outcome reporting?

- Response: Probably yes

- Reason: Same as for previous outcome

6. Was the study apparently free of other problems that could put it at risk of bias?

- Response: Definitely yes

- Reason: Same as for previous outcome

In summary, this randomized controlled trial appears to have a low risk of bias based on the details reported. Key domains like randomization, blinding, and low dropout rates were addressed adequately. Some details were lacking on certain aspects like allocation concealment and sequence generation, but the overall trial design and conduct seem robust.

© 2024 Lai H et al. *JAMA Network Open.*

ID: Randall, 2012

First response:

Based on the review guidelines and information provided in the article, here are the evaluation results for the outcome of total sleep time (TST) reported in Randall et al. (2012):

Article ID: Randall et al., 2012

Outcome Name: Total sleep time (TST)

1. Was the allocation sequence adequately generated?

- Response: Probably no

- Reason: No statements are available on how the randomization sequence was generated. The article only states "Participants passing the physical and mental health screening underwent an 8 hours screening PSG. Subjects were required to demonstrate a screening sleep efficiency ≤ 85% and have no other sleep disorders. Subsequent study 8-h PSGs excluded airflow and leg monitoring. Participants were randomly assigned to placebo or zolpidem (10 mg or 5 mg) administration."

2. Was the allocation adequately concealed?

- Response: Probably no

- Reason: No statements are available on allocation concealment.

3. Blinding: Was knowledge of the allocated interventions adequately prevented?

3.a. Were patients blinded? Probably yes

3.b. Were healthcare providers blinded? Probably yes

3.c. Were data collectors blinded? Probably yes

3.d. Were outcome assessors blinded? Probably yes

3.e. Were data analysts blinded? Probably yes

- Reason: The article states "Participants and researchers were blind to treatment conditions" and "Scorers maintained 90% interrater reliability." This suggests blinding was likely adequate but details are not provided to definitively assess.

© 2024 Lai H et al. *JAMA Network Open.*

4. Was loss to follow-up (missing outcome data) infrequent?

- Response: Probably yes

- Reason: 91 out of 125 participants (72.8%) completed the 8-month study. The dropout rate is less than 20% in all groups.

5. Are reports of the study free of selective outcome reporting?

- Response: Probably yes

- Reason: The study protocol is not available to verify there is no selective outcome reporting, but the pre-specified primary and secondary outcomes appear to be reported as expected.

6. Was the study apparently free of other problems that could put it at a risk of bias?

- Response: Probably yes

- Reason: The study appears to be free of other sources of bias based on the information reported.

© 2024 Lai H et al. *JAMA Network Open.*

ID: Randall, 2012

Second response:

Based on the information provided in the article by Randall et al. (2012) and the guidelines you summarized, here are the risk of bias assessments for the primary outcome of total sleep time (TST):

Article ID: Randall et al., 2012

Outcome Name: Total sleep time (TST)

1. Was the allocation sequence adequately generated?

- Response: Probably no

- Reason: No statements are available on how the randomization sequence was generated.

2. Was allocation adequately concealed?

- Response: Probably no

- Reason: No statements are available on allocation concealment.

3. Blinding: Was knowledge of the allocated interventions adequately prevented?

3a. Were patients blinded? Probably yes

3b. Were healthcare providers blinded? Probably yes

3c. Were data collectors blinded? Probably yes

3d. Were outcome assessors blinded? Probably yes

3e. Were data analysts blinded? Probably yes

- Reason: No specific statements on blinding, but the study is described as "double-blind", suggesting blinding was likely adequate.

4. Was loss to follow-up infrequent?

- Response: Probably yes

- Reason: 91 out of 125 randomized participants (72.8%) completed the study. The dropout rate at 8 months was less than 30%.

© 2024 Lai H et al. *JAMA Network Open.*

5. Are reports of the study free of selective outcome reporting?

- Response: Probably yes

- Reason: The study protocol is not available, but the published report seems to include the expected outcomes.

6. Was the study apparently free of other problems that could put it at high risk of bias?

- Response: Definitely yes

- Reason: The study appears to be free of other biases based on the information reported.

In summary, this randomized controlled trial has some unclear risk of bias due to limited reporting on random sequence generation, allocation concealment, and blinding methods. However, overall the study seems to be at low to moderate risk of bias. Let me know if you would like me to evaluate another outcome from this or another study.

© 2024 Lai H et al. *JAMA Network Open.*

ID: Sivertsen, 2006

First response:

Thank you for providing the detailed guidelines and examples. Based on the information in the Sivertsen et al. (2006) article, here are my evaluation results for the primary outcome of sleep efficiency:

Article ID: Sivertsen, 2006

Outcome Name: Sleep efficiency

1. Was the allocation sequence adequately generated?

- Response: Probably no

- Reason: The article does not provide any statements on how the randomization sequence was generated.

2. Was the allocation adequately concealed?

- Response: Probably yes

- Reason: The article states "Allocation concealment was implemented using sealed, sequentially numbered boxes that were identical in appearance for the 3 treatment groups."

3. Blinding: Was knowledge of the allocated interventions adequately prevented?

3a. Were patients blinded?

- Response: Probably yes

- Reason: The article states that double-blinding was achieved with pills identical in appearance, smell and flavor containing either zopiclone or placebo.

3b. Were healthcare providers blinded?

- Response: Definitely no

- Reason: The article states that CBT condition could not be blinded.

3c. Were data collectors blinded?

© 2024 Lai H et al. *JAMA Network Open.*

- Response: Probably yes

- Reason: The article states that technicians scoring the PSG data were blinded to the participants' condition.

3d. Were outcome assessors blinded?

- Response: Probably yes

- Reason: The article states that technicians scoring the PSG data were blinded to the participants' condition.

3e. Were data analysts blinded?

- Response: Probably yes

- Reason: No statements, but unlikely to affect objective PSG sleep efficiency outcome.

4. Was loss to follow-up infrequent?

- Response: Probably yes

- Reason: 316 participants were randomized, and 304 (96%) completed the 6 week assessment.

5. Are reports of the study free of selective outcome reporting?

- Response: Probably yes

- Reason: No protocol available, but the pre-specified primary outcomes appear to be fully reported.

6. Was the study apparently free of other problems that could put it at a risk of bias?

- Response: Probably yes

- Reason: No other apparent issues noted.

Please let me know if you would like me to evaluate any other outcomes from this study. I will be happy to provide additional assessments following the provided guidelines.

© 2024 Lai H et al. *JAMA Network Open.*

ID: Sivertsen, 2006

Second response:

Thank you for providing the detailed guidelines and examples for evaluating randomized controlled trials (RCTs). I will follow the criteria closely to assess the risk of bias for the RCT and provide my evaluation in the requested format.

Article ID: Sivertsen, 2006

Outcome Name: PSG-measured sleep efficiency

1. Was the allocation sequence adequately generated?

- Response: Probably no

- Reason: The article states "Randomization was performed by the project leader using blocks of 3 with no stratification." However, no specific details are provided on how the randomization sequence was generated.

2. Was the allocation adequately concealed?

- Response: Probably yes

- Reason: The article states "Allocation concealment was implemented using sealed, sequentially numbered boxes that were identical in appearance for the 3 treatment groups."

3. Blinding: Was knowledge of the allocated interventions adequately prevented?

3a. Were patients blinded? Probably yes

- Reason: The article states that double-blinding was achieved with pills identical in appearance, smell and flavor containing either zopiclone or placebo. However, blinding was not possible in the CBT group.

3b. Were healthcare providers blinded? Probably no

- Reason: Blinding of CBT providers was not possible.

3c. Were data collectors blinded? Probably yes

© 2024 Lai H et al. *JAMA Network Open.*

- Reason: No information provided but outcome assessment was blinded.

3d. Were outcome assessors blinded? Definitely yes

- Reason: The article states that "All electrophysiological signals were acquired using Embla A10 (Flaga-Medcare Somnologica 3.2 software package, Buffalo, NY)" suggesting automated outcome assessment.

3e. Were data analysts blinded? Probably yes

- Reason: No information provided but blinding of data analysts is likely.

4. Was loss to follow-up infrequent?

- Response: Probably yes

- Reason: 46 participants were enrolled, 45 completed the 6 week treatment. Only 1 participant discontinued treatment. The follow-up rate is greater than 90% at 6 weeks.

5. Are reports of the study free of selective outcome reporting?

- Response: Probably yes

- Reason: The trial protocol is not available but the published report seems to include all expected outcomes.

6. Was the study apparently free of other problems that could put it at risk of bias?

- Response: Probably yes

- Reason: The study appears to be free of other sources of bias based on the information provided.

Please let me know if I have followed the guidelines correctly and provided the evaluation in the requested format. I am happy to revise my assessment if needed.

© 2024 Lai H et al. *JAMA Network Open.*

ID: Voshaar, 2004

First response:

Based on the guidelines provided, here are the risk of bias assessments for the article:

Article ID: Voshaar2004, 2004

Outcome Name: Total sleep time (TST)

1. Was the allocation sequence adequately generated?

- Response: Probably no

- Reason: No statements are provided on how the randomization sequence was generated.

2. Was allocation adequately concealed?

- Response: Probably no

- Reason: No statements are provided on allocation concealment.

3. Blinding: Was knowledge of the allocated interventions adequately prevented?

3a. Patients blinded?

- Response: Probably yes

- Reason: The study is described as double-blind, and it is unlikely that blinding could have been broken.

3b. Healthcare providers blinded?

- Response: Probably yes

- Reason: The study is described as double-blind, and it is unlikely that blinding could have been broken.

3c. Data collectors blinded?

- Response: Probably yes

- Reason: The study is described as double-blind, and it is unlikely that blinding could have been broken.

© 2024 Lai H et al. *JAMA Network Open.*

3d. Outcome assessors blinded?

- Response: Probably yes

- Reason: The study is described as double-blind, and it is unlikely that blinding could have been broken.

3e. Data analysts blinded?

- Response: Probably yes

- Reason: The study is described as double-blind, and it is unlikely that blinding could have been broken.

4. Was loss to follow-up infrequent?

- Response: Probably yes

- Reason: 159/223 (71.3%) participants completed the trial. The dropout rate is less than 20% in both groups.

5. Are reports of the study free of selective outcome reporting?

- Response: Probably yes

- Reason: The study protocol is not available to verify there is no selective outcome reporting, but the pre-specified primary and secondary outcomes appear to be reported as expected.

6. Was the study apparently free of other problems that could put it at a risk of bias?

- Response: Probably yes

- Reason: The study appears to be free of other sources of bias.

© 2024 Lai H et al. *JAMA Network Open.*

ID: Voshaar, 2004

Second response:

Based on the guidelines provided, here are the evaluation results for the article:

Article ID: Voshaar2004, 2004

Outcome Name: Total sleep time

1. Was the allocation sequence adequately generated?

- Probably no.

- No statements are provided on how the randomization sequence was generated.

2. Was the allocation adequately concealed?

- Probably no.

- No statements are provided on allocation concealment.

3. Blinding: Was knowledge of the allocated interventions adequately prevented?

3a. Were patients blinded?

- Probably yes.

- Study medications were supplied in matching gelatin capsules.

3b. Were healthcare providers blinded?

- Probably yes.

- Study is described as double-blind.

3c. Were data collectors blinded?

- Probably yes.

- Study is described as double-blind.

3d. Were outcome assessors blinded?

- Probably yes.

© 2024 Lai H et al. *JAMA Network Open.*

- Study is described as double-blind.

3e. Were data analysts blinded?

- Probably yes.

- Study is described as double-blind.

4. Was loss to follow-up infrequent?

- Probably yes.

- 159/223 (71.3%) participants completed the study. The dropout rate is less than 20% in all groups.

5. Are reports of the study free of selective outcome reporting?

- Probably yes.

- The study protocol is not available to verify there is no selective outcome reporting, but the pre-specified primary and secondary outcomes appear to be reported as expected.

6. Was the study apparently free of other problems that could put it at a risk of bias?

- Probably yes.

- The study appears to be free of other sources of bias.

© 2024 Lai H et al. *JAMA Network Open.*

ID: Xu, 2020

First response:

Based on the guidelines and information provided, here is my assessment of the risk of bias for the RCT:

Article ID: Xu, 2020

Outcome Name: Sleep onset latency

1. Was the allocation sequence adequately generated?

- Probably yes

- The study states "The random allocation of the patients into the melatonin or placebo group was conducted using the random number method by the responsible clinician." Although details of how the random numbers were generated are not provided, the use of random number generation suggests adequate sequence generation.

2. Was allocation adequately concealed?

- Probably yes

- The study states "Randomized allocation was concealed in sequentially numbered, opaque, sealed envelopes." The use of sequentially numbered, opaque, sealed envelopes suggests adequate allocation concealment.

3. Blinding: Was knowledge of the allocated interventions adequately prevented?

3a. Were patients blinded?

- Probably yes

- The study states "The patients and investigators that assessing outcomes were blind to the random allocation of the treatment group (double-blind)." The use of identical placebo and blinding of patients suggests knowledge of allocation was adequately prevented.

3b. Were healthcare providers blinded?

- Probably yes

© 2024 Lai H et al. *JAMA Network Open.*

- The study states "The patients and investigators that assessing outcomes were blind to the random allocation of the treatment group (double-blind)." The blinding of investigators suggests healthcare providers were blinded.

3c. Were data collectors blinded?

- Probably yes

- The study states "The patients and investigators that assessing outcomes were blind to the random allocation of the treatment group (double-blind)." The blinding of investigators suggests data collectors were blinded.

3d. Were outcome assessors blinded?

- Probably yes

- The study states "The patients and investigators that assessing outcomes were blind to the random allocation of the treatment group (double-blind)." The blinding of those assessing outcomes suggests outcome assessors were blinded.

3e. Were data analysts blinded?

- Probably yes

- The study states "Blinding was kept by using placebo with identical appearance, smell and taste of the melatonin tablet. The blinding was not to be broken (unless in emergency) until the data was collected and verified for analyses." The blinding of data analysts was maintained until after data analysis.

4. Was loss to follow-up infrequent?

- Probably yes

- 97 participants were randomized, and 61 were included in the final analysis. Although the dropout rate is over 30%, the study states that exclusions were due to presence of sleep apnea or periodic leg movements, which were unlikely related to the outcome measures. The reasons for dropout seem unlikely to be related to the outcome.

5. Are reports of the study free of selective outcome reporting?

© 2024 Lai H et al. *JAMA Network Open.*

- Probably yes

- The study protocol is not available, but the pre-specified primary outcome of sleep onset latency and other expected sleep outcomes are reported. There is no clear evidence that selective reporting occurred.

6. Was the study apparently free of other problems that could put it at a risk of bias?

- Probably yes

- The study appears to be free of other sources of bias.

Article ID: Xu, 2020

Outcome Name: Total sleep time

1. Was the allocation sequence adequately generated?

- Probably yes

- The study states "The random allocation of the patients into the melatonin or placebo group was conducted using the random number method by the responsible clinician." Although details of how the random numbers were generated are not provided, the use of random number generation suggests adequate sequence generation.

2. Was allocation adequately concealed?

- Probably yes

- The study states "Randomized allocation was concealed in sequentially numbered, opaque, sealed envelopes." The use of sequentially numbered, opaque, sealed envelopes suggests adequate allocation concealment.

3. Blinding: Was knowledge of the allocated interventions adequately prevented?

3a. Were patients blinded?

- Probably yes

© 2024 Lai H et al. *JAMA Network Open.*

- The study states "The patients and investigators that assessing outcomes were blind to the random allocation of the treatment group (double-blind)." The use of identical placebo and blinding of patients suggests knowledge of allocation was adequately prevented.

3b. Were healthcare providers blinded?

- Probably yes
- The study states "The patients and investigators that assessing outcomes were blind to the random allocation of the treatment group (double-blind)." The blinding of investigators suggests healthcare providers were blinded.

3c. Were data collectors blinded?

- Probably yes
- The study states "The patients and investigators that assessing outcomes were blind to the random allocation of the treatment group (double-blind)." The blinding of investigators suggests data collectors were blinded.

3d. Were outcome assessors blinded?

- Probably yes
- The study states "The patients and investigators that assessing outcomes were blind to the random allocation of the treatment group (double-blind)." The blinding of those assessing outcomes suggests outcome assessors were blinded.

3e. Were data analysts blinded?

- Probably yes
- The study states "Blinding was kept by using placebo with identical appearance, smell and taste of the melatonin tablet. The blinding was not to be broken (unless in emergency) until the data was collected and verified for analyses." The blinding of data analysts was maintained until after data analysis.

4. Was loss to follow-up infrequent?

- Probably yes

© 2024 Lai H et al. *JAMA Network Open.*

- 97 participants were randomized, and 61 were included in the final analysis. Although the dropout rate is over 30%, the study states that exclusions were due to presence of sleep apnea or periodic leg movements, which were unlikely related to the outcome measures. The reasons for dropout seem unlikely to be related to the outcome.

5. Are reports of the study free of selective outcome reporting?

- Probably yes

- The study protocol is not available, but the pre-specified primary outcome of sleep onset latency and other expected sleep outcomes are reported. There is no clear evidence that selective reporting occurred.

6. Was the study apparently free of other problems that could put it at a risk of bias?

- Probably yes

- The study appears to be free of other sources of bias.

© 2024 Lai H et al. *JAMA Network Open.*

ID: Xu, 2020

Second response:

Based on the guidelines and RCT details you provided, here is my assessment of the risk of bias:

Article ID: Xu et al., 2020

Outcome Name: Sleep latency

1. Was the allocation sequence adequately generated?

- Response: Probably yes

- Reason: The article states "The random allocation of the patients into the melatonin or placebo group was conducted using the random number method by the responsible clinician." Although details are not provided on how the random numbers were generated, the use of random numbers suggests the sequence was probably adequately generated.

2. Was allocation adequately concealed?

- Response: Probably no

- Reason: No statements are provided on allocation concealment.

3. Blinding:

3a. Were patients blinded?

- Response: Probably yes

- Reason: The article states "The patients and investigators that assessing outcomes were blind to the random allocation of the treatment group (double-blind)." Although details on blinding methods are not provided, the double-blinding suggests patients were probably adequately blinded.

3b. Were healthcare providers blinded?

- Response: Probably yes

- Reason: Same justification as 3a. The double-blinding suggests healthcare providers were probably adequately blinded.

© 2024 Lai H et al. *JAMA Network Open.*

3c. Were data collectors blinded?

- Response: Probably yes

- Reason: Same justification as 3a. The double-blinding suggests data collectors were probably adequately blinded.

3d. Were outcome assessors blinded?

- Response: Probably yes

- Reason: Same justification as 3a. The double-blinding suggests outcome assessors were probably adequately blinded.

3e. Were data analysts blinded?

- Response: Probably yes

- Reason: Same justification as 3a. The double-blinding suggests data analysts were probably adequately blinded.

4. Was loss to follow-up infrequent?

- Response: Probably yes

- Reason: 97 of the 111 randomized patients completed the study. This corresponds to a completion rate of 87%, which is between 80-90%. The article does not provide reasons for loss to follow-up.

5. Are reports free of selective outcome reporting?

- Response: Probably yes

- Reason: The study protocol is not available, but the published report seems to include pre-specified outcomes related to sleep latency.

6. Was the study apparently free of other problems that could put it at a risk of bias?

- Response: Probably yes

- Reason: The study appears to be free of other sources of bias based on the information provided.

Article ID: Xu et al., 2020

© 2024 Lai H et al. *JAMA Network Open.*

Outcome Name: Total sleep time

1. Was the allocation sequence adequately generated?

- Response: Probably yes

- Reason: Same justification as for sleep latency outcome

2. Was allocation adequately concealed?

- Response: Probably no

- Reason: Same justification as for sleep latency outcome

3. Blinding:

- Responses: Probably yes for 3a to 3e.

- Reasons: Same justifications as for sleep latency outcome

4. Was loss to follow-up infrequent?

- Response: Probably yes

- Reason: Same justification as for sleep latency outcome

5. Are reports free of selective outcome reporting?

- Response: Probably yes

- Reason: Same justification as for sleep latency outcome

6. Was the study apparently free of other problems that could put it at a risk of bias?

- Response: Probably yes

- Reason: Same justification as for sleep latency outcome

Please let me know if you would like me to assess additional outcomes from this RCT or if you have any other feedback on my evaluation. I am happy to revise my assessments as needed.

© 2024 Lai H et al. *JAMA Network Open.*

ID: Yan, 2013

First response:

Thank you for providing the detailed guidelines and instructions. I will follow them closely to evaluate the risk of bias for the RCT you provided. Here is my assessment:

Article ID: Yan, 2013

Outcome Name: Change in total score on Sleep Dysfunction Rating Scale (SDRS) from baseline to 14 days

1. Was the allocation sequence adequately generated?

- Response: Probably no

- Reason: The article states "Randomizing chart was obtained by Statistical Analysis System (SAS) program with balanced allocation randomization and applied to blind labeling." However, no specific details are provided on how the randomization sequence was generated.

2. Was the allocation adequately concealed?

- Response: Probably no

- Reason: No statements are made regarding allocation concealment.

3a. Were patients blinded?

- Response: Probably yes

- Reason: This was a double-blind study. Patients received either brotizolam or estazolam along with a placebo matching the medication they did not receive. This should adequately blind patients to treatment assignment.

3b. Were healthcare providers blinded?

- Response: Probably yes

- Reason: This was a double-blind study, so healthcare providers were likely adequately blinded.

3c. Were data collectors blinded?

© 2024 Lai H et al. *JAMA Network Open.*

- Response: Probably yes

- Reason: No direct statements, but blinding of data collectors is likely since this was a double-blind trial.

3d. Were outcome assessors blinded?

- Response: Probably yes

- Reason: No direct statements, but blinding of outcome assessors is likely since this was a double-blind trial.

3e. Were data analysts blinded?

- Response: Probably yes

- Reason: No direct statements, but blinding of data analysts is likely since this was a double-blind trial.

4. Was loss to follow-up infrequent?

- Response: Probably yes

- Reason: 253 patients were randomized, and 251 were included in the full analysis set. Only 2 patients did not receive treatment. The follow-up rate was >99%, so loss to follow-up appears infrequent.

5. Are reports of the study free of selective outcome reporting?

- Response: Probably yes

- Reason: The protocol is not available but the pre-specified primary and secondary outcomes appear to be reported as expected.

6. Was the study apparently free of other problems that could put it at a risk of bias?

- Response: Probably yes

- Reason: No other apparent sources of bias. The study appears to be a well-conducted double-blind RCT.

© 2024 Lai H et al. *JAMA Network Open.*

Please let me know if you would like me to evaluate the risk of bias for any other outcomes reported in this study. I will be happy to provide assessments following the guidelines you provided.

© 2024 Lai H et al. *JAMA Network Open.*

ID: Yan, 2013

Second response:

Based on the guidelines provided and the information reported in the article "A randomized, double-blind, double-dummy, multicenter, controlled trial on brotizolam intervention in outpatients with insomnia" by Yan et al. (2013), here is the risk of bias assessment for the primary outcome of change in Sleep Dysfunction Rating Scale (SDRS) total score:

Article ID: Yan et al., 2013

Outcome Name: Change in SDRS total score

1. Was the allocation sequence adequately generated?

- Response: Probably yes

- Reason: The article states "Randomizing chart was obtained by Statistical Analysis System (SAS) program with balanced allocation randomization and applied to blind labeling." Computer generated randomization methods are adequate.

2. Was allocation adequately concealed?

- Response: Probably no

- Reason: No statements about allocation concealment.

3. Blinding: Was knowledge of the allocated interventions adequately prevented?

3a. Were patients blinded?

- Response: Probably yes

- Reason: The study is described as double-blind. Placebos were used to maintain blinding.

3b. Were healthcare providers blinded?

- Response: Probably yes

- Reason: The study is described as double-blind. Placebos were used to maintain blinding.

3c. Were data collectors blinded?

© 2024 Lai H et al. *JAMA Network Open.*

- Response: Probably yes

- Reason: The study is described as double-blind. Placebos were used to maintain blinding.

3d. Were outcome assessors blinded?

- Response: Probably yes

- Reason: The study is described as double-blind. Placebos were used to maintain blinding.

3e. Were data analysts blinded?

- Response: Probably yes

- Reason: The study is described as double-blind. Placebos were used to maintain blinding.

4. Was loss to follow-up infrequent?

- Response: Definitely yes

- Reason: 121/126 (96.0%) in the brotizolam group and 119/127 (93.7%) in the estazolam group completed the study. Dropout rate was less than 10% in both groups.

5. Are reports of the study free of selective outcome reporting?

- Response: Probably yes

- Reason: The pre-specified primary outcome of change in SDRS total score was reported. No study protocol available to verify other outcomes.

6. Was the study apparently free of other problems that could put it at a risk of bias?

- Response: Probably yes

- Reason: The study appears to be free of other sources of bias.

© 2024 Lai H et al. *JAMA Network Open.*

eTable 1 The reference standard response for each randomized controlled trial and domain

No	ID	Outcome	1. Was the allocation sequence adequately generated?	2. Was the allocation adequately concealed?	3.a. Were patients blinded?	3.b. Were healthcare providers blinded?	3.c. Were data collectors blinded?	3.d. Were outcome assessors blinded?	3.e. Were data analysts blinded?	4. Was loss to follow-up (missing outcome data) infrequent?	5. Are reports of the study free of selective outcome reporting?	6. Was the study apparently free of other problems that could put it at a risk of bias?
1	Benassi-Evans, 2009	Weight	High risk	High risk	High risk	High risk	High risk	High risk	High risk	Low risk	Low risk	Low risk
2	Davis, 2017	Home-measured BP	Low risk	High risk	High risk	High risk	High risk	High risk	High risk	Low risk	Low risk	Low risk
3	de Mello, 2008	Urinary albumin	High risk	High risk	High risk	High risk	High risk	High risk	High risk	Low risk	Low risk	Low risk

© 2024 Lai H et al. *JAMA Network Open.*

		excretion rate										
4	Griffin, 2013	Weight	Low risk	High risk	Low risk	High risk	High risk	High risk	High risk	High risk	Low risk	Low risk
5	Hunninghake, 2000	LDL Cholesterol	High risk	High risk	High risk	High risk	High risk	High risk	High risk	Low risk	Low risk	Low risk
6	Lanza, 2007	Adenoma recurrence	High risk	High risk	High risk	High risk	High risk	High risk	High risk	High risk	Low risk	Low risk
7	Murphy, 2012	Weight	Low risk	High risk	High risk	High risk	High risk	High risk	High risk	Low risk	Low risk	Low risk
8	Poddar, 2013	Weight	High risk	High risk	High risk	High risk	High risk	High risk	High risk	High risk	Low risk	Low risk
9	Turner-McGrievy, 2015	Weight	Low risk	High risk	High risk	High risk	High risk	High risk	High risk	High risk	Low risk	Low risk
10	Yaskolka, 2019	Serum iron	High risk	High risk	High risk	High risk	High risk	High risk	High risk	Low risk	Low risk	Low risk
11	Carlson, 2019	HbA1c	High risk	High risk	High risk	High risk	High risk	High risk	High risk	Low risk	Low risk	Low risk

© 2024 Lai H et al. *JAMA Network Open.*

12	Cherney, 2021	HbA1c	High risk	High risk	Low risk	Low risk	Low risk	Low risk	Low risk	Low risk	Low risk	High risk
13	del Prato, 2014	HbA1c	High risk	High risk	Low risk	Low risk	Low risk	Low risk	Low risk	High risk	Low risk	Low risk
14	Frias, 2018	HbA1c	High risk	High risk	Low risk	Low risk	Low risk	Low risk	Low risk	Low risk	Low risk	Low risk
15	Gao, 2020	HbA1c	High risk	Low risk	Low risk	Low risk	Low risk	Low risk	Low risk	Low risk	Low risk	Low risk
16	Ikonomidis, 2020	Left ventricular global longitudinal strain	High risk	High risk	High risk	High risk	Low risk	Low risk	Low risk	Low risk	Low risk	Low risk
17	Nahra, 2021	HbA1c	Low risk	High risk	Low risk	Low risk	Low risk	Low risk	Low risk	Low risk	Low risk	Low risk
18	Seino, 2015	HbA1c	High risk	High risk	Low risk	Low risk	Low risk	Low risk	High risk	Low risk	Low risk	Low risk
19	Taskinen, 2011	HbA1c	High risk	High risk	Low risk	Low risk	Low risk	Low risk	Low risk	Low risk	Low risk	Low risk
20	Yabiku, 2017	HbA1c	Low risk	High risk	High risk	High risk	High risk	High risk	High risk	Low risk	Low risk	High risk

© 2024 Lai H et al. *JAMA Network Open.*

21	Allen, 1987	Sleep latency	High risk	High risk	Low risk	Low risk	High risk	High risk	High risk	Low risk	Low risk	Low risk
22	Black, 2017	Wake time after sleep onset	High risk	High risk	Low risk	Low risk	Low risk	Low risk	Low risk	Low risk	Low risk	Low risk
23	Fan, 2017	Total sleep time	Low risk	Low risk	Low risk	Low risk	Low risk	Low risk	Low risk	Low risk	Low risk	Low risk
24	Lankford, 2012	Total sleep time	Low risk	Low risk	Low risk	Low risk	Low risk	Low risk	Low risk	Low risk	Low risk	Low risk
25	Mignot, 2022	Wake time after sleep onset	Low risk	Low risk	Low risk	Low risk	Low risk	Low risk	Low risk	Low risk	Low risk	Low risk
26	Randall, 2012	Total sleep time	High risk	High risk	Low risk	Low risk	Low risk	Low risk	Low risk	High risk	Low risk	Low risk
27	Sivertsen, 2006	Sleep efficiency	High risk	Low risk	Low risk	High risk	Low risk	Low risk	Low risk	Low risk	Low risk	Low risk
28	Voshaar, 2004	Total sleep time	High risk	High risk	Low risk	Low risk	Low risk	Low risk	Low risk	Low risk	Low risk	Low risk

© 2024 Lai H et al. *JAMA Network Open.*

29	Xu, 2020	Sleep onset latency	Low risk	Low risk	Low risk	Low risk	Low risk	Low risk	Low risk	Low risk	Low risk	Low risk
30	Yan, 2013	Change of Sleep Dysfunction Rating Scale	Low risk	High risk	Low risk	Low risk	Low risk	Low risk	Low risk	Low risk	Low risk	Low risk

© 2024 Lai H et al. *JAMA Network Open.*

eTable 2 Thresholds to interpret the values for Cohen κ

Cohen's Kappa	Interpretation
0	No agreement
0.01 - 0.20	Slight agreement
0.21 - 0.40	Fair agreement
0.41 - 0.60	Moderate agreement
0.61 - 0.80	Substantial agreement
0.81 - 0.99	Near perfect agreement
1	Perfect agreement

Refer to:

1. Stats: What is a Kappa coefficient? (Cohen's Kappa). Accessed November 5, 2023. http://www.pmean.com/definitions/kappa.htm
2. Zach. Cohen's Kappa Statistic: Definition & Example. Statology. Published February 22, 2021. Accessed November 17, 2023. https://www.statology.org/cohens-kappa-statistic/

© 2024 Lai H et al. *JAMA Network Open.*

eTable 3 The results of assessment by LLM1 for each domain and RCT

No	Time spent (second)	Reviewer	1. Was the allocation sequence adequately generated?	2. Was the allocation adequately concealed?	3.a. Were patients blinded?	3.b. Were healthcare providers blinded?	3.c. Were data collectors blinded?	3.d. Were outcome assessors blinded?	3.e. Were data analysts blinded?	4. Was loss to follow-up (missing outcome data) infrequent?	5. Are reports of the study free of selective outcome reporting?	6. Was the study apparently free of other problems that could put it at a risk of bias?
1	66	ChatGPT - 1	High risk	High risk	High risk	High risk	High risk	High risk	High risk	Low risk	Low risk	Low risk
1	81	ChatGPT - 2	High risk	High risk	High risk	High risk	High risk	High risk	High risk	High risk	High risk	High risk
2	62	ChatGPT - 1	Low risk	High risk	High risk	High risk	High risk	High risk	High risk	Low risk	Low risk	Low risk
2	70	ChatGPT - 2	High risk	High risk	High risk	High risk	High risk	High risk	High risk	High risk	High risk	High risk
3	55	ChatGPT - 1	Low risk	High risk	High risk	High risk	High risk	High risk	High risk	Low risk	Low risk	Low risk
3	79	ChatGPT - 2	Low risk	High risk	High risk	High risk	High risk	High risk	High risk	Low risk	Low risk	Low risk
4	64	ChatGPT - 1	Low risk	Low risk	High risk	High risk	High risk	High risk	High risk	Low risk	Low risk	Low risk
4	72	ChatGPT - 2	Low risk	Low risk	Low risk	High risk	High risk	High risk	High risk	Low risk	Low risk	Low risk

© 2024 Lai H et al. *JAMA Network Open.*

5	56	ChatGPT - 1	Low risk	High risk	High risk	High risk	High risk	High risk	High risk	Low risk	Low risk	Low risk
5	79	ChatGPT - 2	Low risk	High risk	High risk	High risk	High risk	High risk	High risk	Low risk	Low risk	Low risk
6	72	ChatGPT - 1	Low risk	Low risk	High risk	High risk	High risk	High risk	High risk	Low risk	Low risk	Low risk
6	63	ChatGPT - 2	Low risk	Low risk	High risk	High risk	Low risk	Low risk	Low risk	Low risk	Low risk	Low risk
7	73	ChatGPT - 1	Low risk	High risk	High risk	High risk	High risk	High risk	High risk	Low risk	Low risk	Low risk
7	58	ChatGPT - 2	High risk	High risk	High risk	High risk	High risk	High risk	High risk	High risk	Low risk	Low risk
8	81	ChatGPT - 1	Low risk	High risk	High risk	High risk	High risk	High risk	High risk	High risk	Low risk	Low risk
8	61	ChatGPT - 2	Low risk	High risk	High risk	High risk	High risk	High risk	High risk	High risk	Low risk	Low risk
9	114	ChatGPT - 1	Low risk	High risk	High risk	High risk	High risk	High risk	High risk	Low risk	Low risk	Low risk
9	82	ChatGPT - 2	Low risk	High risk	High risk	High risk	High risk	High risk	High risk	Low risk	Low risk	Low risk
10	89	ChatGPT - 1	Low risk	High risk	High risk	High risk	High risk	High risk	High risk	Low risk	Low risk	Low risk
10	64	ChatGPT - 2	Low risk	High risk	High risk	High risk	High risk	High risk	High risk	Low risk	Low risk	Low risk
11	82	ChatGPT - 1	High risk	High risk	High risk	High risk	High risk	High risk	High risk	Low risk	Low risk	Low risk
11	75	ChatGPT - 2	Low risk	Low risk	High risk	High risk	High risk	High risk	High risk	Low risk	Low risk	Low risk
12	57	ChatGPT - 1	Low risk	Low risk	Low risk	Low risk	Low risk	Low risk	Low risk	Low risk	Low risk	Low risk
12	93	ChatGPT - 2	Low risk	Low risk	Low risk	Low risk	Low risk	Low risk	Low risk	Low risk	Low risk	Low risk
13	73	ChatGPT - 1	High risk	High risk	Low risk	Low risk	Low risk	Low risk	Low risk	Low risk	Low risk	Low risk

© 2024 Lai H et al. *JAMA Network Open.*

13	62	ChatGPT - 2	Low risk	Low risk	Low risk	Low risk	Low risk	Low risk	Low risk	Low risk	Low risk	Low risk
14	52	ChatGPT - 1	High risk	High risk	Low risk	Low risk	Low risk	Low risk	Low risk	Low risk	Low risk	Low risk
14	81	ChatGPT - 2	Low risk	Low risk	Low risk	Low risk	Low risk	Low risk	Low risk	Low risk	Low risk	Low risk
15	114	ChatGPT - 1	High risk	High risk	Low risk	Low risk	Low risk	Low risk	Low risk	Low risk	High risk	Low risk
15	73	ChatGPT - 2	Low risk	Low risk	Low risk	Low risk	Low risk	Low risk	Low risk	Low risk	Low risk	Low risk
16	87	ChatGPT - 1	Low risk	Low risk	High risk	High risk	Low risk	Low risk	Low risk	Low risk	Low risk	Low risk
16	70	ChatGPT - 2	High risk	High risk	High risk	High risk	High risk	High risk	High risk	Low risk	Low risk	Low risk
17	56	ChatGPT - 1	Low risk	Low risk	Low risk	Low risk	Low risk	Low risk	Low risk	Low risk	Low risk	Low risk
17	78	ChatGPT - 2	Low risk	Low risk	Low risk	Low risk	Low risk	Low risk	Low risk	Low risk	Low risk	Low risk
18	104	ChatGPT - 1	High risk	High risk	High risk	High risk	High risk	High risk	High risk	Low risk	Low risk	Low risk
18	75	ChatGPT - 2	Low risk	Low risk	Low risk	Low risk	Low risk	Low risk	Low risk	Low risk	Low risk	Low risk
19	69	ChatGPT - 1	Low risk	High risk	Low risk	Low risk	Low risk	Low risk	Low risk	Low risk	Low risk	Low risk
19	91	ChatGPT - 2	Low risk	Low risk	Low risk	Low risk	Low risk	Low risk	Low risk	Low risk	Low risk	Low risk
20	79	ChatGPT - 1	High risk	High risk	High risk	High risk	High risk	Low risk	High risk	Low risk	Low risk	Low risk
20	92	ChatGPT - 2	Low risk	High risk	High risk	High risk	High risk	High risk	High risk	Low risk	Low risk	Low risk
21	58	ChatGPT - 1	High risk	High risk	Low risk	Low risk	High risk	High risk	High risk	Low risk	Low risk	Low risk
21	82	ChatGPT - 2	Low risk	High risk	Low risk	Low risk	High risk	High risk	High risk	Low risk	Low risk	Low risk

© 2024 Lai H et al. *JAMA Network Open.*

22	103	ChatGPT - 1	High risk	High risk	Low risk	High risk	High risk	High risk	High risk	Low risk	High risk	Low risk
22	67	ChatGPT - 2	High risk	High risk	Low risk	Low risk	Low risk	Low risk	Low risk	Low risk	Low risk	Low risk
23	85	ChatGPT - 1	Low risk	Low risk	Low risk	Low risk	Low risk	Low risk	Low risk	Low risk	Low risk	Low risk
23	69	ChatGPT - 2	Low risk	Low risk	Low risk	Low risk	Low risk	Low risk	Low risk	Low risk	Low risk	Low risk
24	96	ChatGPT - 1	Low risk	Low risk	Low risk	Low risk	Low risk	Low risk	Low risk	Low risk	Low risk	Low risk
24	74	ChatGPT - 2	Low risk	High risk	Low risk	Low risk	High risk	High risk	High risk	Low risk	High risk	Low risk
25	53	ChatGPT - 1	Low risk	Low risk	Low risk	Low risk	Low risk	Low risk	Low risk	Low risk	Low risk	Low risk
25	82	ChatGPT - 2	Low risk	Low risk	Low risk	Low risk	Low risk	Low risk	Low risk	Low risk	Low risk	Low risk
26	78	ChatGPT - 1	High risk	High risk	Low risk	Low risk	Low risk	Low risk	Low risk	Low risk	High risk	Low risk
26	67	ChatGPT - 2	High risk	High risk	Low risk	Low risk	Low risk	Low risk	Low risk	Low risk	High risk	Low risk
27	127	ChatGPT - 1	High risk	High risk	High risk	High risk	Low risk	Low risk	Low risk	Low risk	Low risk	Low risk
27	105	ChatGPT - 2	High risk	High risk	High risk	High risk	Low risk	Low risk	Low risk	Low risk	Low risk	Low risk
28	95	ChatGPT - 1	High risk	High risk	Low risk	Low risk	Low risk	Low risk	Low risk	Low risk	Low risk	Low risk
28	69	ChatGPT - 2	High risk	High risk	Low risk	Low risk	Low risk	Low risk	Low risk	Low risk	Low risk	Low risk
29	81	ChatGPT - 1	High risk	High risk	Low risk	Low risk	Low risk	Low risk	Low risk	Low risk	High risk	Low risk
29	73	ChatGPT - 2	Low risk	Low risk	Low risk	Low risk	Low risk	Low risk	High risk	Low risk	Low risk	Low risk
30	54	ChatGPT - 1	High risk	High risk	Low risk	Low risk	Low risk	Low risk	Low risk	Low risk	High risk	Low risk

© 2024 Lai H et al. *JAMA Network Open.*

30	93	ChatGPT - 2	Low risk	High risk	Low risk	Low risk	Low risk	Low risk	Low risk	Low risk	High risk	Low risk

© 2024 Lai H et al. *JAMA Network Open.*

eTable 4 The results of assessment by LLM2 for each RCT and domain

No	Time spent (second)	Reviewer	1. Was the allocation sequence adequately generated?	2. Was the allocation adequately concealed?	3.a. Were patients blinded?	3.b. Were healthcare providers blinded?	3.c. Were data collectors blinded?	3.d. Were outcome assessors blinded?	3.e. Were data analysts blinded?	4. Was loss to follow-up (missing outcome data) infrequent?	5. Are reports of the study free of selective outcome reporting?	6. Was the study apparently free of other problems that could put it at a risk of bias?
1	45	Claude - 1	High risk	High risk	High risk	High risk	High risk	High risk	High risk	Low risk	Low risk	Low risk
1	38	Claude - 2	High risk	High risk	High risk	High risk	High risk	High risk	High risk	High risk	Low risk	Low risk
2	45	Claude - 1	Low risk	High risk	High risk	High risk	High risk	High risk	High risk	Low risk	Low risk	Low risk
2	49	Claude - 2	Low risk	Low risk	High risk	High risk	High risk	High risk	High risk	Low risk	Low risk	Low risk
3	71	Claude - 1	High risk	High risk	High risk	High risk	High risk	High risk	High risk	Low risk	Low risk	Low risk
3	46	Claude - 2	Low risk	Low risk	High risk	High risk	High risk	High risk	High risk	Low risk	Low risk	Low risk
4	46	Claude - 1	Low risk	High risk	Low risk	High risk	High risk	High risk	High risk	High risk	Low risk	Low risk
4	55	Claude - 2	Low risk	High risk	High risk	High risk	High risk	High risk	High risk	Low risk	Low risk	Low risk

© 2024 Lai H et al. *JAMA Network Open.*

5	38	Claude - 1	Low risk	High risk	High risk	High risk	High risk	High risk	High risk	Low risk	Low risk	Low risk
5	45	Claude - 2	High risk	High risk	High risk	High risk	High risk	High risk	High risk	High risk	High risk	High risk
6	54	Claude - 1	High risk	High risk	High risk	High risk	High risk	High risk	High risk	High risk	Low risk	Low risk
6	43	Claude - 2	High risk	High risk	High risk	High risk	High risk	High risk	High risk	High risk	Low risk	Low risk
7	47	Claude - 1	Low risk	High risk	High risk	High risk	High risk	High risk	High risk	Low risk	Low risk	Low risk
7	63	Claude - 2	High risk	High risk	High risk	High risk	High risk	High risk	High risk	High risk	Low risk	Low risk
8	56	Claude - 1	Low risk	High risk	High risk	High risk	High risk	High risk	High risk	High risk	Low risk	Low risk
8	44	Claude - 2	Low risk	High risk	High risk	High risk	High risk	High risk	High risk	High risk	Low risk	Low risk
9	52	Claude - 1	Low risk	High risk	High risk	High risk	High risk	High risk	High risk	Low risk	Low risk	Low risk
9	74	Claude - 2	Low risk	High risk	High risk	High risk	High risk	High risk	High risk	Low risk	Low risk	Low risk
10	46	Claude - 1	Low risk	High risk	High risk	High risk	High risk	High risk	High risk	Low risk	Low risk	Low risk
10	57	Claude - 2	Low risk	High risk	High risk	High risk	High risk	High risk	High risk	Low risk	Low risk	Low risk
11	55	Claude - 1	High risk	High risk	High risk	High risk	Low risk	Low risk	Low risk	Low risk	High risk	Low risk
11	49	Claude - 2	High risk	High risk	High risk	High risk	Low risk	Low risk	Low risk	Low risk	Low risk	Low risk
12	50	Claude - 1	High risk	High risk	Low risk	Low risk	Low risk	Low risk	Low risk	Low risk	High risk	Low risk
12	39	Claude - 2	Low risk	Low risk	Low risk	Low risk	Low risk	Low risk	Low risk	Low risk	Low risk	Low risk
13	43	Claude - 1	High risk	High risk	Low risk	Low risk	Low risk	Low risk	Low risk	Low risk	Low risk	Low risk

© 2024 Lai H et al. *JAMA Network Open.*

13	57	Claude - 2	Low risk	Low risk	Low risk	Low risk	Low risk	Low risk	Low risk	Low risk	Low risk	Low risk
14	44	Claude - 1	Low risk	Low risk	Low risk	Low risk	Low risk	Low risk	Low risk	Low risk	Low risk	Low risk
14	44	Claude - 2	Low risk	Low risk	Low risk	Low risk	Low risk	Low risk	Low risk	Low risk	Low risk	Low risk
15	38	Claude - 1	High risk	Low risk	Low risk	Low risk	Low risk	Low risk	Low risk	Low risk	Low risk	Low risk
15	42	Claude - 2	High risk	Low risk	Low risk	Low risk	Low risk	Low risk	Low risk	Low risk	Low risk	Low risk
16	48	Claude - 1	High risk	High risk	High risk	High risk	Low risk	Low risk	Low risk	Low risk	Low risk	Low risk
16	52	Claude - 2	High risk	High risk	High risk	High risk	High risk	High risk	High risk	Low risk	Low risk	Low risk
17	36	Claude - 1	Low risk	High risk	Low risk	High risk	High risk	High risk	High risk	Low risk	Low risk	Low risk
17	57	Claude - 2	Low risk	Low risk	Low risk	Low risk	Low risk	Low risk	Low risk	Low risk	Low risk	Low risk
18	87	Claude - 1	High risk	High risk	Low risk	Low risk	Low risk	Low risk	High risk	Low risk	High risk	Low risk
18	60	Claude - 2	High risk	High risk	Low risk	Low risk	Low risk	Low risk	High risk	Low risk	High risk	Low risk
19	63	Claude - 1	High risk	High risk	Low risk	Low risk	Low risk	Low risk	Low risk	Low risk	Low risk	Low risk
19	56	Claude - 2	High risk	High risk	Low risk	Low risk	Low risk	Low risk	Low risk	Low risk	Low risk	Low risk
20	57	Claude - 1	Low risk	High risk	High risk	High risk	High risk	High risk	High risk	Low risk	Low risk	Low risk
20	45	Claude - 2	Low risk	High risk	High risk	High risk	High risk	High risk	High risk	Low risk	High risk	High risk
21	49	Claude - 1	High risk	High risk	Low risk	Low risk	Low risk	Low risk	Low risk	Low risk	Low risk	Low risk
21	42	Claude - 2	High risk	High risk	Low risk	Low risk	High risk	High risk	High risk	Low risk	Low risk	Low risk

© 2024 Lai H et al. *JAMA Network Open.*

22	61	Claude - 1	High risk	High risk	Low risk	Low risk	Low risk	Low risk	Low risk	Low risk	High risk	Low risk
22	46	Claude - 2	High risk	High risk	Low risk	High risk	High risk	High risk	High risk	Low risk	Low risk	Low risk
23	62	Claude - 1	Low risk	Low risk	Low risk	Low risk	Low risk	Low risk	Low risk	Low risk	Low risk	Low risk
23	83	Claude - 2	Low risk	Low risk	Low risk	Low risk	Low risk	Low risk	Low risk	Low risk	Low risk	Low risk
24	48	Claude - 1	Low risk	High risk	Low risk	Low risk	Low risk	Low risk	Low risk	Low risk	Low risk	Low risk
24	48	Claude - 2	Low risk	High risk	Low risk	Low risk	Low risk	Low risk	Low risk	Low risk	Low risk	Low risk
25	50	Claude - 1	Low risk	Low risk	Low risk	Low risk	Low risk	Low risk	Low risk	Low risk	Low risk	Low risk
25	61	Claude - 2	Low risk	Low risk	Low risk	Low risk	Low risk	Low risk	Low risk	Low risk	Low risk	Low risk
26	39	Claude - 1	High risk	High risk	Low risk	Low risk	Low risk	Low risk	Low risk	Low risk	Low risk	Low risk
26	84	Claude - 2	High risk	High risk	Low risk	Low risk	Low risk	Low risk	Low risk	Low risk	Low risk	Low risk
27	63	Claude - 1	High risk	Low risk	Low risk	High risk	Low risk	Low risk	Low risk	Low risk	Low risk	Low risk
27	74	Claude - 2	High risk	Low risk	Low risk	High risk	Low risk	Low risk	Low risk	Low risk	Low risk	Low risk
28	70	Claude - 1	High risk	High risk	Low risk	Low risk	Low risk	Low risk	Low risk	Low risk	Low risk	Low risk
28	78	Claude - 2	High risk	High risk	Low risk	Low risk	Low risk	Low risk	Low risk	Low risk	Low risk	Low risk
29	50	Claude - 1	Low risk	Low risk	Low risk	Low risk	Low risk	Low risk	Low risk	Low risk	Low risk	Low risk
29	58	Claude - 2	Low risk	High risk	Low risk	Low risk	Low risk	Low risk	Low risk	Low risk	Low risk	Low risk
30	37	Claude - 1	High risk	High risk	Low risk	Low risk	Low risk	Low risk	Low risk	Low risk	Low risk	Low risk

© 2024 Lai H et al. *JAMA Network Open.*

30	54	Claude - 2	Low risk	High risk	Low risk	Low risk	Low risk	Low risk	Low risk	Low risk	Low risk	Low risk

© 2024 Lai H et al. *JAMA Network Open.*

eTable 5 The domain-specific accuracy and consistency of assessments by LLM1.

Domain	Correct assessment rate	Consistent assessment rate	TP	TN	FP	FN	Sensitivity	Specificity	Precision	F-score	Cohen's Kappa	PABAK
1	56.67%	60.00%	17	17	5	21	0.45	0.77	0.77	0.57	0.54	0.20
2	70.00%	70.00%	34	7	5	14	0.71	0.58	0.87	0.78	0.65	0.40
3.a	93.33%	93.33%	24	32	4	0	1.00	0.89	0.86	0.92	0.92	0.87
3.b	96.67%	96.67%	29	30	1	0	1.00	0.97	0.97	0.98	0.96	0.93
3.c	93.33%	86.67%	26	30	3	1	0.96	0.91	0.90	0.93	0.85	0.73
3.d	91.67%	83.33%	25	30	3	2	0.93	0.91	0.89	0.91	0.81	0.67
3.e	91.67%	83.33%	27	28	3	2	0.93	0.90	0.90	0.92	0.81	0.67
4	78.33%	90.00%	2	45	3	10	0.17	0.94	0.40	0.24	0.87	0.80
5	83.33%	80.00%	0	50	10	0	Not available	0.83	0.00	Not available	0.76	0.60
6	90.00%	93.33%	0	54	2	4	0.00	0.96	0.00	Not available	0.91	0.87

The results are calculated based on the 60 evaluations (two evaluations per each of the 30 RCTs).

© 2024 Lai H et al. *JAMA Network Open.*

eTable 6 The domain-specific accuracy and consistency of assessments by LLM2.

Domain	Correct assessment rate	Consistent assessment rate	TP	TN	FP	FN	Sensitivity	Specificity	Precision	F-score	Cohen's Kappa	PABAK
1	80.0%	80.0%	28	20	2	10	0.74	0.91	0.93	0.82	0.77	0.60
2	83.3%	80.0%	41	9	3	7	0.85	0.75	0.93	0.89	0.76	0.60
3.a	98.3%	96.7%	24	35	1	0	1.00	0.97	0.96	0.98	0.96	0.93
3.b	96.7%	93.3%	28	30	2	0	1.00	0.94	0.93	0.97	0.92	0.87
3.c	90.0%	86.7%	23	31	3	3	0.88	0.91	0.88	0.88	0.85	0.73
3.d	90.0%	86.7%	23	31	3	3	0.88	0.91	0.88	0.88	0.85	0.73
3.e	90.0%	86.7%	25	29	3	3	0.89	0.91	0.89	0.89	0.85	0.73
4	83.3%	86.7%	5	45	3	7	0.42	0.94	0.63	0.50	0.84	0.73
5	90.0%	86.7%	1	53	6	0	1.00	0.90	0.14	0.25	0.83	0.73
6	93.3%	93.3%	1	55	1	3	0.25	0.98	0.50	0.33	0.91	0.87

The results are calculated based on the 60 evaluations (two evaluations per each of the 30 RCTs).

© 2024 Lai H et al. *JAMA Network Open.*

eTable 7 The study-specific accuracy of assessments by LLM1.

Study No	Reviewer	TP	TN	FP	FN	Correct assessment rate	Sensitivity	Specificity	Precision	F-score
1	ChatGPT - 1	7	3	0	0	100.0%	1.00	1.00	1.00	1.00
1	ChatGPT - 2	7	0	3	0	70.0%	1.00	0.00	0.70	0.82
2	ChatGPT - 1	6	4	0	0	100.0%	1.00	1.00	1.00	1.00
2	ChatGPT - 2	6	0	4	0	60.0%	1.00	0.00	0.60	0.75
3	ChatGPT - 1	6	3	0	1	90.0%	0.86	1.00	1.00	0.92
3	ChatGPT - 2	6	3	0	1	90.0%	0.86	1.00	1.00	0.92
4	ChatGPT - 1	4	3	1	2	70.0%	0.67	0.75	0.80	0.73
4	ChatGPT - 2	4	4	0	2	80.0%	0.67	1.00	1.00	0.80
5	ChatGPT - 1	6	3	0	1	90.0%	0.86	1.00	1.00	0.92
5	ChatGPT - 2	6	3	0	1	90.0%	0.86	1.00	1.00	0.92
6	ChatGPT - 1	5	2	0	3	70.0%	0.63	1.00	1.00	0.77
6	ChatGPT - 2	2	2	0	6	40.0%	0.25	1.00	1.00	0.40
7	ChatGPT - 1	6	4	0	0	100.0%	1.00	1.00	1.00	1.00
7	ChatGPT - 2	6	2	2	0	80.0%	1.00	0.50	0.75	0.86

© 2024 Lai H et al. *JAMA Network Open.*

8	ChatGPT - 1	7	2	0	1	90.0%	0.88	1.00	1.00	0.93
8	ChatGPT - 2	7	2	0	1	90.0%	0.88	1.00	1.00	0.93
9	ChatGPT - 1	6	3	0	1	90.0%	0.86	1.00	1.00	0.92
9	ChatGPT - 2	6	3	0	1	90.0%	0.86	1.00	1.00	0.92
10	ChatGPT - 1	6	3	0	1	90.0%	0.86	1.00	1.00	0.92
10	ChatGPT - 2	6	3	0	1	90.0%	0.86	1.00	1.00	0.92
11	ChatGPT - 1	7	3	0	0	100.0%	1.00	1.00	1.00	1.00
11	ChatGPT - 2	5	3	0	2	80.0%	0.71	1.00	1.00	0.83
12	ChatGPT - 1	0	7	0	3	70.0%	0.00	1.00	Not available	Not available
12	ChatGPT - 2	0	7	0	3	70.0%	0.00	1.00	Not available	Not available
13	ChatGPT - 1	2	7	0	1	90.0%	0.67	1.00	1.00	0.80
13	ChatGPT - 2	0	7	0	3	70.0%	0.00	1.00	Not available	Not available
14	ChatGPT - 1	2	8	0	0	100.0%	1.00	1.00	1.00	1.00
14	ChatGPT - 2	0	8	0	2	80.0%	0.00	1.00	Not available	Not available
15	ChatGPT - 1	1	7	2	0	80.0%	1.00	0.78	0.33	0.50
15	ChatGPT - 2	0	9	0	1	90.0%	0.00	1.00	Not available	Not available
16	ChatGPT - 1	2	6	0	2	80.0%	0.50	1.00	1.00	0.67

© 2024 Lai H et al. *JAMA Network Open.*

16	ChatGPT - 2	4	3	3	0	70.0%	1.00	0.50	0.57	0.73
17	ChatGPT - 1	0	9	0	1	90.0%	0.00	1.00	Not available	Not available
17	ChatGPT - 2	0	9	0	1	90.0%	0.00	1.00	Not available	Not available
18	ChatGPT - 1	3	3	4	0	60.0%	1.00	0.43	0.43	0.60
18	ChatGPT - 2	0	7	0	3	70.0%	0.00	1.00	Not available	Not available
19	ChatGPT - 1	1	8	0	1	90.0%	0.50	1.00	1.00	0.67
19	ChatGPT - 2	0	8	0	2	80.0%	0.00	1.00	Not available	Not available
20	ChatGPT - 1	5	2	1	2	70.0%	0.71	0.67	0.83	0.77
20	ChatGPT - 2	6	3	0	1	90.0%	0.86	1.00	1.00	0.92
21	ChatGPT - 1	5	5	0	0	100.0%	1.00	1.00	1.00	1.00
21	ChatGPT - 2	4	5	0	1	90.0%	0.80	1.00	1.00	0.89
22	ChatGPT - 1	6	3	1	0	90.0%	1.00	0.75	0.86	0.92
22	ChatGPT - 2	2	8	0	0	100.0%	1.00	1.00	1.00	1.00
23	ChatGPT - 1	0	10	0	0	100.0%	Not available	1.00	Not available	Not available
23	ChatGPT - 2	0	10	0	0	100.0%	Not available	1.00	Not available	Not available
24	ChatGPT - 1	0	10	0	0	100.0%	Not available	1.00	Not available	Not available
24	ChatGPT - 2	0	5	5	0	50.0%	Not available	0.50	0.00	Not available

© 2024 Lai H et al. *JAMA Network Open.*

25	ChatGPT - 1	0	10	0	0	100.0%	Not available	1.00	Not available	Not available
25	ChatGPT - 2	0	10	0	0	100.0%	Not available	1.00	Not available	Not available
26	ChatGPT - 1	2	6	1	1	80.0%	0.67	0.86	0.67	0.67
26	ChatGPT - 2	2	6	1	1	80.0%	0.67	0.86	0.67	0.67
27	ChatGPT - 1	2	6	2	0	80.0%	1.00	0.75	0.50	0.67
27	ChatGPT - 2	2	6	2	0	80.0%	1.00	0.75	0.50	0.67
28	ChatGPT - 1	2	8	0	0	100.0%	1.00	1.00	1.00	1.00
28	ChatGPT - 2	2	8	0	0	100.0%	1.00	1.00	1.00	1.00
29	ChatGPT - 1	0	7	3	0	70.0%	Not available	0.70	0.00	Not available
29	ChatGPT - 2	0	9	1	0	90.0%	Not available	0.90	0.00	Not available
30	ChatGPT - 1	1	7	2	0	80.0%	1.00	0.78	0.33	0.50
30	ChatGPT - 2	1	8	1	0	90.0%	1.00	0.89	0.50	0.67

The values of true and false positives and negatives refer to the ten domains of the risk of bias assessment.

© 2024 Lai H et al. *JAMA Network Open.*

eTable 8 The study-specific accuracy of assessments by LLM2.

Study No	Reviewer	TP	TN	FP	FN	Correct assessment rate	Sensitivity	Specificity	Precision	F-score
1	Claude - 1	7	3	0	0	100.0%	1.00	1.00	1.00	1.00
1	Claude - 2	7	2	1	0	90.0%	1.00	0.67	0.88	0.93
2	Claude - 1	6	4	0	0	100.0%	1.00	1.00	1.00	1.00
2	Claude - 2	5	4	0	1	90.0%	0.83	1.00	1.00	0.91
3	Claude - 1	7	3	0	0	100.0%	1.00	1.00	1.00	1.00
3	Claude - 2	5	3	0	2	80.0%	0.71	1.00	1.00	0.83
4	Claude - 1	6	4	0	0	100.0%	1.00	1.00	1.00	1.00
4	Claude - 2	5	3	1	1	80.0%	0.83	0.75	0.83	0.83
5	Claude - 1	6	3	0	1	90.0%	0.86	1.00	1.00	0.92
5	Claude - 2	7	0	3	0	70.0%	1.00	0.00	0.70	0.82
6	Claude - 1	8	2	0	0	100.0%	1.00	1.00	1.00	1.00
6	Claude - 2	8	2	0	0	100.0%	1.00	1.00	1.00	1.00
7	Claude - 1	6	4	0	0	100.0%	1.00	1.00	1.00	1.00
7	Claude - 2	6	2	2	0	80.0%	1.00	0.50	0.75	0.86

© 2024 Lai H et al. *JAMA Network Open.*

8	Claude - 1	7	2	0	1	90.0%	0.88	1.00	1.00	0.93
8	Claude - 2	7	2	0	1	90.0%	0.88	1.00	1.00	0.93
9	Claude - 1	6	3	0	1	90.0%	0.86	1.00	1.00	0.92
9	Claude - 2	6	3	0	1	90.0%	0.86	1.00	1.00	0.92
10	Claude - 1	6	3	0	1	90.0%	0.86	1.00	1.00	0.92
10	Claude - 2	6	3	0	1	90.0%	0.86	1.00	1.00	0.92
11	Claude - 1	4	2	1	3	60.0%	0.57	0.67	0.80	0.67
11	Claude - 2	4	3	0	3	70.0%	0.57	1.00	1.00	0.73
12	Claude - 1	3	6	0	1	90.0%	0.75	1.00	1.00	0.86
12	Claude - 2	0	7	0	3	70.0%	0.00	1.00	Not available	Not available
13	Claude - 1	2	7	0	1	90.0%	0.67	1.00	1.00	0.80
13	Claude - 2	0	7	0	3	70.0%	0.00	1.00	Not available	Not available
14	Claude - 1	0	8	0	2	80.0%	0.00	1.00	Not available	Not available
14	Claude - 2	0	8	0	2	80.0%	0.00	1.00	Not available	Not available
15	Claude - 1	1	9	0	0	100.0%	1.00	1.00	1.00	1.00
15	Claude - 2	1	9	0	0	100.0%	1.00	1.00	1.00	1.00
16	Claude - 1	4	6	0	0	100.0%	1.00	1.00	1.00	1.00

© 2024 Lai H et al. *JAMA Network Open.*

16	Claude - 2	4	3	3	0	70.0%	1.00	0.50	0.57	0.73
17	Claude - 1	1	5	4	0	60.0%	1.00	0.56	0.20	0.33
17	Claude - 2	0	9	0	1	90.0%	0.00	1.00	Not available	Not available
18	Claude - 1	3	6	1	0	90.0%	1.00	0.86	0.75	0.86
18	Claude - 2	3	6	1	0	90.0%	1.00	0.86	0.75	0.86
19	Claude - 1	2	8	0	0	100.0%	1.00	1.00	1.00	1.00
19	Claude - 2	2	8	0	0	100.0%	1.00	1.00	1.00	1.00
20	Claude - 1	6	3	0	1	90.0%	0.86	1.00	1.00	0.92
20	Claude - 2	7	2	1	0	90.0%	1.00	0.67	0.88	0.93
21	Claude - 1	2	5	0	3	70.0%	0.40	1.00	1.00	0.57
21	Claude - 2	5	5	0	0	100.0%	1.00	1.00	1.00	1.00
22	Claude - 1	2	7	1	0	90.0%	1.00	0.88	0.67	0.80
22	Claude - 2	2	4	4	0	60.0%	1.00	0.50	0.33	0.50
23	Claude - 1	0	10	0	0	100.0%	Not available	1.00	Not available	Not available
23	Claude - 2	0	10	0	0	100.0%	Not available	1.00	Not available	Not available
24	Claude - 1	0	9	1	0	90.0%	Not available	0.90	0.00	Not available
24	Claude - 2	0	9	1	0	90.0%	Not available	0.90	0.00	Not available

© 2024 Lai H et al. *JAMA Network Open.*

25	Claude - 1	0	10	0	0	100.0%	Not available	1.00	Not available	Not available
25	Claude - 2	0	10	0	0	100.0%	Not available	1.00	Not available	Not available
26	Claude - 1	2	7	0	1	90.0%	0.67	1.00	1.00	0.80
26	Claude - 2	2	7	0	1	90.0%	0.67	1.00	1.00	0.80
27	Claude - 1	2	8	0	0	100.0%	1.00	1.00	1.00	1.00
27	Claude - 2	2	8	0	0	100.0%	1.00	1.00	1.00	1.00
28	Claude - 1	2	8	0	0	100.0%	1.00	1.00	1.00	1.00
28	Claude - 2	2	8	0	0	100.0%	1.00	1.00	1.00	1.00
29	Claude - 1	0	10	0	0	100.0%	Not available	1.00	Not available	Not available
29	Claude - 2	0	9	1	0	90.0%	Not available	0.90	0.00	Not available
30	Claude - 1	1	8	1	0	90.0%	1.00	0.89	0.50	0.67
30	Claude - 2	1	9	0	0	100.0%	1.00	1.00	1.00	1.00

The values of true and false positives and negatives refer to the ten domains of the risk of bias assessment.

© 2024 Lai H et al. *JAMA Network Open.*

eTable 9 The reason and example of wrong assessments for each domain.

Domain	Wrong assessment	Reason for wrong assessment			
		Valid rationale but wrong assessment	Example	Wrong rationale and assessment	Example
1	38	30 (78.95%)	Poddar, 2013; ChatGPT-1:<br>"*1. Was the allocation sequence adequately generated?*<br>*- Probably yes*<br>*- The study states 'Prior to Visit 1, eligible participants were randomized into one of two treatment groups' but does not provide details on how the randomization sequence was generated.*"<br>The model explicitly acknowledged the lack of sufficient evidence: "*but does not provide details on how the randomization sequence was generated*" but inferred a wrong assessment based on speculation.	8 (21.05%)	Murphy, 2012; Claude-2:<br>"*1. Was the allocation sequence adequately generated?*<br>*- Probably no. No details are provided on how the randomization sequence was generated.*"<br>The model failed to provide a valid rationale "*No details are provided on how the randomization sequence was generated*" and subsequently delivered a wrong assessment.
2	29	21 (72.41%)	Griffin, 2013; ChatGPT-2:<br>"*2. Was the allocation adequately concealed?*<br>*- Probably yes*<br>*- The article does not provide details on allocation concealment, but states that participants were blinded to the diet macronutrient composition, indicating probable adequate concealment.*"	8 (27.59%)	Lankford, 2012; Claude-1:<br>"*2. Was the allocation adequately concealed?*<br>*- Response: Probably no*<br>*- Reason: No statements are provided on allocation concealment.*"

© 2024 Lai H et al. *JAMA Network Open.*

			The model explicitly acknowledged the lack of sufficient evidence but inferred a wrong assessment based on speculation.		The model failed to provide a valid rationale "*No statements are provided on allocation concealment*" and subsequently delivered a wrong assessment.
3.a	5	2 (40.00%)	Griffin, 2013; ChatGPT-1:<br>"*3a. Were patients blinded?*<br>*- Probably no*<br>*- The paper states 'Participants were blind to diet macronutrient composition' but they were informed about differences in meat type between diets, so blinding of patients was likely inadequate.*"<br>The model explicitly acknowledged the evidence but inferred a wrong assessment based on speculation.	3 (60.00%)	Sivertsen, 2006; ChatGPT-1:<br>"*3a. Were patients blinded?*<br>*- Response: Probably no*<br>*- Reason: Patients in the CBT group were not blinded.*"<br>The model failed to provide a valid rationale "*Patients in the CBT group were not blinded*" and subsequently delivered a wrong assessment.
3.b	3	1 (33.33%)	Seino, 2015; ChatGPT-1:<br>"*3b. Were healthcare providers blinded?*<br>*- Response: Probably no*<br>*- Reason: The article only states this was a 'double-blind' study, but there is insufficient information to determine if healthcare providers were actually blinded.*"<br>The model explicitly acknowledged the lack of sufficient evidence but inferred a wrong assessment based on speculation.	2 (66.67%)	Black, 2017; Claude-1:<br>"*3b. Were healthcare providers blinded?*<br>*- Probably no*<br>*- No details provided.*"<br>The model failed to provide a valid rationale "*No details provided*" and subsequently delivered a wrong assessment.
3.c	10	5 (50.00%)	Carlson, 2019; Claude-1:<br>"*3c. Were data collectors blinded? Probably yes*<br>*- The study is described as 'open-label' so patients and providers were aware of treatment assignments. However, outcome assessment was likely blinded.*"	5 (50.00%)	Ikonomidis, 2020; Claude-2:<br>"*3c. Were data collectors blinded?*<br>*- Response: Definitely no*<br>*- Reason: The study does not mention blinding of data collectors.*"

© 2024 Lai H et al. *JAMA Network Open.*

			The model explicitly acknowledged the lack of sufficient evidence but inferred a wrong assessment based on speculation.		The model failed to provide a valid rationale "*The study does not mention blinding of data collectors*" and subsequently delivered a wrong assessment.
3.d	11	6 (54.55%)	Yabiku, 2017; ChatGPT-1:<br>"*3d. Were outcome assessors blinded?*<br>*- Probably yes*<br>*- The study states that HbA1c measurements were carried out by a central laboratory, which implies outcome assessors were likely blinded.*"<br>The model explicitly acknowledged the lack of sufficient evidence but inferred a wrong assessment based on speculation.	5 (45.45%)	Nahra, 2021; Claude-2:<br>"*3d. Were outcome assessors blinded?*<br>*- Response: Probably no*<br>*- Reason: No details provided on blinding of outcome assessors.*"<br>The model failed to provide a valid rationale and subsequently delivered a wrong assessment.
3.e	10	4 (40.00%)	Lanza, 2007; ChatGPT-2:<br>"*3c. Were data collectors blinded?*<br>*- Probably yes*<br>*- Blinding of data collectors is not specified but may have been feasible.*"<br>The model explicitly acknowledged the lack of sufficient evidence: "*Blinding of data collectors is not specified*" but inferred a wrong assessment based on speculation.	6 (60.00%)	Xu, 2020; ChatGPT-2:<br>"*3e. Were data analysts blinded?*<br>*- Response: Probably no*<br>*- Reason: No statements on blinding of data analysts.*"<br>The model failed to provide a valid rationale and subsequently delivered a wrong assessment.
4	23	17 (73.91%)	Turner-McGrievy, 2015; ChatGPT-1:<br>"*4. Was loss to follow-up (missing outcome data) infrequent?*<br>*- Probably yes. The article states "At six months, 50 (79%) completed the study (i.e., provided a body-weight measurement at 6 months)". The follow-up rate was 79%, which is close to 80%.*"	6 (26.09%)	Murphy, 2012; Claude-2:<br>"*4. Was loss to follow-up infrequent?*<br>*- Probably no. No detail is available to support the assessment.*"<br>The model failed to provide a valid rationale and subsequently delivered a wrong assessment.

© 2024 Lai H et al. *JAMA Network Open.*

			The model explicitly acknowledged the evidence but inferred a wrong assessment based on speculation.		
5	16	0 (0.00%)	None.	16 (100.00%)	Black, 2017; Claude-1:<br>"*5. Are reports of the study free of selective outcome reporting?*<br>*- Response: Probably no*<br>*- Reason: The study protocol is not available to verify all expected outcomes were reported.*"<br>The model correctly identified the absence of protocol but does not assess selective outcome reporting based on the descriptions in the method section as required in the guidance.
6	10	3 (30.00%)	Hunninghake, 2000; Claude-2:<br>"*6. Was the study apparently free of other problems that could put it at a risk of bias?*<br>*- Probably no*<br>*- No details are provided to determine if other potential sources of bias exist.*"<br>The model did not identify other issues that could pose a risk of bias to the study, but inferred a wrong assessment based on speculation.	7 (70.00%)	Yabiku, 2017; ChatGPT-1:<br>"*6. Was the study apparently free of other problems that could put it at risk of bias?*<br>*- Probably yes*<br>*- The study appears free of other sources of bias based on the information provided.*"<br>The model failed to identify other issues that could pose a risk of bias to the study.

© 2024 Lai H et al. *JAMA Network Open.*

eTable 10 The domain-specific consistency between four assessments by both LLM1 and LLM2.

Domain	Agreement across four assessments	Total	Proportion of observed agreement
1	13	30	43.3%
2	14	30	46.7%
3.a	26	30	86.7%
3.b	27	30	90.0%
3.c	22	30	73.3%
3.d	21	30	70.0%
3.e	22	30	73.3%
4	24	30	80.0%
5	18	30	60.0%
6	24	30	80.0%

© 2024 Lai H et al. *JAMA Network Open.*

eTable 11 The study-specific consistency between two assessments by LLM1.

Study No	Agreement across two assessments	Proportion of observed agreement	Proportion of expected agreement	Cohen's Kappa	PABAK
1	7	70.0%	49.0%	0.41	0.40
2	7	70.0%	36.0%	0.53	0.40
3	10	100.0%	45.0%	1.00	1.00
4	9	90.0%	28.0%	0.86	0.80
5	10	100.0%	45.0%	1.00	1.00
6	7	70.0%	14.0%	0.65	0.40
7	8	80.0%	44.0%	0.64	0.60
8	10	100.0%	53.0%	1.00	1.00
9	10	100.0%	45.0%	1.00	1.00
10	10	100.0%	45.0%	1.00	1.00
11	8	80.0%	44.0%	0.64	0.60
12	10	100.0%	49.0%	1.00	1.00
13	8	80.0%	49.0%	0.61	0.60
14	8	80.0%	64.0%	0.44	0.60

© 2024 Lai H et al. *JAMA Network Open.*

15	7	70.0%	63.0%	0.19	0.40
16	5	50.0%	26.0%	0.32	0.00
17	10	100.0%	81.0%	1.00	1.00
18	3	30.0%	21.0%	0.11	-0.40
19	9	90.0%	64.0%	0.72	0.80
20	8	80.0%	36.0%	0.69	0.60
21	9	90.0%	45.0%	0.82	0.80
22	9	90.0%	36.0%	0.84	0.80
23	10	100.0%	100.0%	Not available	1.00
24	5	50.0%	50.0%	0.00	0.00
25	10	100.0%	100.0%	Not available	1.00
26	10	100.0%	40.0%	1.00	1.00
27	10	100.0%	40.0%	1.00	1.00
28	10	100.0%	68.0%	1.00	1.00
29	6	60.0%	63.0%	-0.08	0.20
30	9	90.0%	57.0%	0.77	0.80

The results compare the agreement between the two assessments by the same tool.

© 2024 Lai H et al. *JAMA Network Open.*

eTable 12 The study-specific consistency between two assessments by LLM2.

Study No	Agreement across two assessments	Proportion of observed agreement	Proportion of expected agreement	Cohen's Kappa	PABAK
1	9	90.0%	55.0%	0.78	0.80
2	9	90.0%	46.0%	0.81	0.80
3	8	80.0%	44.0%	0.64	0.60
4	8	80.0%	42.0%	0.66	0.60
5	6	60.0%	42.0%	0.31	0.20
6	10	100.0%	68.0%	1.00	1.00
7	8	80.0%	44.0%	0.64	0.60
8	10	100.0%	53.0%	1.00	1.00
9	10	100.0%	45.0%	1.00	1.00
10	10	100.0%	45.0%	1.00	1.00
11	9	90.0%	22.0%	0.87	0.80
12	8	80.0%	42.0%	0.66	0.60
13	8	80.0%	49.0%	0.61	0.60
14	10	100.0%	64.0%	1.00	1.00

© 2024 Lai H et al. *JAMA Network Open.*

15	10	100.0%	82.0%	1.00	1.00
16	7	70.0%	34.0%	0.55	0.40
17	5	50.0%	45.0%	0.09	0.00
18	9	90.0%	45.0%	0.82	0.80
19	10	100.0%	68.0%	1.00	1.00
20	8	80.0%	48.0%	0.62	0.60
21	7	70.0%	35.0%	0.54	0.40
22	5	50.0%	32.0%	0.26	0.00
23	10	100.0%	100.0%	Not available	1.00
24	10	100.0%	81.0%	1.00	1.00
25	10	100.0%	100.0%	Not available	1.00
26	10	100.0%	53.0%	1.00	1.00
27	10	100.0%	68.0%	1.00	1.00
28	10	100.0%	68.0%	1.00	1.00
29	9	90.0%	90.0%	0.00	0.80
30	9	90.0%	73.0%	0.63	0.80

The results compare the agreement between the two assessments by the same tool.

© 2024 Lai H et al. *JAMA Network Open.*

eTable 13 The study-specific consistency between four assessments by both LLM1 and LLM2.

Study No	Agreement across four assessments	Proportion of observed agreement
1	7	70%
2	5	50%
3	8	80%
4	7	70%
5	6	60%
6	4	40%
7	8	80%
8	9	90%
9	9	90%
10	9	90%
11	4	40%
12	8	80%
13	8	80%
14	8	80%

© 2024 Lai H et al. *JAMA Network Open.*

15	7	70%
16	5	50%
17	5	50%
18	2	20%
19	8	80%
20	6	60%
21	6	60%
22	5	50%
23	10	100%
24	5	50%
25	10	100%
26	9	90%
27	8	80%
28	10	100%
29	6	60%
30	8	80%

© 2024 Lai H et al. *JAMA Network Open.*
