# Supplementary material for: Assessing the Risk of Bias in Randomized Clinical Trials With Large Language Models
Source: JAMA Netw Open. 2024 May 22;7(5):e2412687. doi: 10.1001/jamanetworkopen.2024.12687 (PMC11112444; doi:10.1001/jamanetworkopen.2024.12687)
Supplement: Supplement 2. — Data Sharing Statement [file jamanetwopen-e2412687-s002.pdf]

Data Sharing Statement

Lai. Assessing the Risk of Bias in Randomized Clinical Trials with Large Language Models. *JAMA Netw Open*. Published May 22, 2024. doi:10.1001/jamanetworkopen.2024.12687

Data
Data available: Yes
Data types: Data (not involving human participants)
How to access data: All the data and information of this study are provided in the attachment.
When available: beginning date: 08-10-2023, end date: 10-30-2023

Supporting Documents
Document types: None

Additional Information
Who can access the data: Not available.
Types of analyses: Not available.
Mechanisms of data availability: Not available.
Any additional restrictions: Not available.
